# CuAAC-Based Synthesis, Copper-Catalyzed Aldehyde-Forming Hydrolytic Fission and Antiproliferative Evaluation of Novel Ferrocenoylamino-Substituted Triazole-Tethered Quinine–Chalcone Hybrids

**DOI:** 10.3390/molecules29020375

**Published:** 2024-01-11

**Authors:** António Dembo, Etelka Ferenczi, Tamás Jernei, Andrea Bor, Zsuzsanna Schelz, István Zupkó, Szilárd Varga, Antal Csámpai

**Affiliations:** 1 Department of Organic Chemistry, Eötvös Loránd University (ELTE), Pázmány P. sétány 1/A, H-1117 Budapest, Hungary; diandjungudembo2@gmail.com (A.D.); etelkaferenczi@gmail.com (E.F.); tamas.jernei@ttk.elte.hu (T.J.); 2 Hevesy György PhD School of Chemistry, Pázmány P. sétány 1/A, H-1117 Budapest, Hungary; 3 Institute of Pharmacodynamics and Biopharmacy, University of Szeged, Eötvös u. 6., H-6720 Szeged, Hungary; bor.gytk@gmail.com (A.B.); schelz.zsuzsanna@szte.hu (Z.S.); zupko.istvan@szte.hu (I.Z.); 4 HUN-REN Research Centre for Natural Sciences, Institute of Organic Chemistry, Magyar Tudósok Krt 2., H-1117 Budapest, Hungary; varga.szilard@ttk.hu

**Keywords:** quinine, chalcone, 1,2,3-triazole, CuAAC, hybrid compounds, enone hydrolysis, DFT modeling, cytotoxicity, structure–activity relationships

## Abstract

A series of novel triazole-tethered ferrocenoylamino-substituted cinchona–chalcone hybrids along with two representative benzoylamino-substituted reference compounds were prepared by three methods of CuAAC chemistry. In line with the limited success or complete failure of attempted conversions with low catalyst loadings, by means of DFT modeling studies, we demonstrated that a substantial part of the Cu(I) ions can be chelated and thus trapped in the aroylamino-substituted cinchona fragment and all of the accessible coordinating sites of the chalcone residues. Accordingly, increased amounts of catalysts were used to achieve acceptable yields; however, the cycloadditions with para-azidochalcones were accompanied by partial or complete aldehyde-forming hydrolytic fission of the enone C=C bond in a substituent-, solvent- and copper load-dependent manner. The experienced hydrolytic stability of the hybrids obtained by cycloadditions with ortho-azidochalcones was interpreted in terms of relative energetics, DFT reactivity indices and MO analysis of simplified models of two isomer copper–enone complexes. The novel hybrids were evaluated on HeLa, MDA-MB-231 and A2780 cell lines and showed substantial activity at low-to-submicromolar concentrations. An organometallic model carrying 3,4,5-trimethoxyphenyl residue in the enone part with a para-disubstituted benzene ring in the central skeletal region was identified as the most potent antiproliferative lead, characterized by submicromolar IC_50_ values measured on the three investigated cells. The biological assays also disclosed that this ferrocenoylamino-containing lead compound displays a ca. two- to five-fold more substantial antiproliferative effect than its benzoylamino-substituted counterpart.

## 1. Introduction

Different types of cancer are continuously among the leading causes of death on a global scale, with poor prognosis and low survival rates [1,2]. In fighting these devastating diseases, chemotherapy is recognized as one of the most important tools for the treatment of malignancies, often in combination with other therapies, such as surgery, radiation or hormone therapy. However, the efficacy of most anticancer chemotherapies in clinical practice is decreased by various circumstances including multidrug resistance (MDR) [3,4] and severe adverse effects. It follows that there is a strong need for the development of more potent novel drugs featuring enhanced selectivity and activity, with preferable capability of overcoming MDR. One of the most promising new strategies in the elaboration of improved chemotherapy is the fragment-based drug design and synthesis of hybrid compounds by coupling a reasonable selection of pharmacophore fragments [5,6,7]. Such hybrid drugs capable of interacting with more than one cellular molecular target can be considered highly potent anticancer agents with enhanced efficiency in triggering cell death by multiplied mechanisms, thus having a real potential to overcome typical disadvantages of single anticancer agents such as resistance and adverse effects. For an expansion of novel potent therapeutic agents, the implication of compounds of natural origin and their chemically modified versions also seems an attractive strategy. In this regard, several representatives from alkaloid families are of pronounced interest [8,9,10,11]. 

Despite the fact that the easily available cinchona alkaloids are known not to possess any activity against *neoplasma malignum*, they have shown the ability to reduce the effect of multidrug resistance (MDR) in intense combined chemotherapy [12,13]. The inclusion of some of their representatives in combined therapy aims to increase the efficiency and lower the toxic effects of the actual anticancer agents [12]**.** However, it must be pointed out here that hybrid molecules comprising two quinine fragments attached to a central ferrocene-1,1′-dicarboxamide core in a complex scaffold of *C_2_*-symmetry displays strong cytotoxic and cytostatic effects in low- and submicromolar doses on human cancer cell lines [14]**.** Accordingly, from the aspect of fragment-based drug design, ferrocene carboxamide attached to a cinchona unit can be considered a potent building block in anticancer drug candidates. This view has also been supported by the successful development of aminosugar-based ferrocene carboxamides with substantial anticancer activity on human malignant cell lines [15] including HeLa and MDA-MB-231, which were also targeted by our novel hybrid compounds (cf. Section 2.3). On the other hand, due to its stability, super-aromaticity, substituent-dependent, fine-tunable, ROS-generating redox transformations and increased membrane-penetrating ability, ferrocene is the most prominent structural motif in the rapidly emerging group of anticancer organometallics with versatile mechanisms of action [16,17,18,19,20,21,22,23]. In this context, it has also been demonstrated that the replacement of an aromatic nucleus of certain organic compounds with a ferrocene unit can lead to products possessing antiproliferative activity, which is absent or less manifested in the parent molecule [24,25,26]. 

Chalcones also represent a highly potent class of small molecule anticancer drug candidates triggering manifold mechanisms of actions in the tumorous cells, including cell cycle arrest, inhibitory activity in tubulin polymerization, and enzyme dynamics [27,28,29,30,31,32]. In the context of molecular mechanisms, it is of particular significance that due to their Michael acceptor character featuring pronounced electrophilicity toward the cysteine sulfhydryl group, chalcones with appropriate substitution patterns can also be covalent inhibitors of nuclear factor κB [33] implicated in oncogenic signaling pathways that promote tumor cell proliferation and survival [34]. It has also been demonstrated that an array of structurally diverse chalcones are potential agents in therapies directed to overcome MDR with substantial in vitro and in vivo efficacy on both drug-susceptible and drug-resistant cancers. These chalcones are typically capable of targeting aromatase, vascular endothelial growth factor (VEGF) and breast cancer resistance protein (BCRP), as well as ATP binding cassette subfamily G member 2 (ABCG2) [35,36]. In addition to the induction of apoptosis in the tumorous cells, the anticancer mechanisms of chalcones typically involve invasion and metastasis, preventing an antiangiogenic effect, as well as the regulation of cancer epigenetics [37]. Finally, it is worth underlining that triazole-coupled chalcone motifs in diverse scaffolds have proven to be emerging pharmacophores in bioactive molecules, as extensively exemplified in a recent review [38].

**Figure 1:**
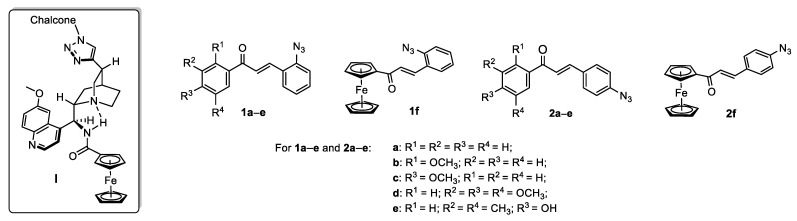
General type of the targeted hybrids (**I**) and the chalcone components used as azide partners in the CuAAc coupling reactions.

Supporting the view on the real potential of the strategy of molecular design based on the combination of the aforementioned pharmacophoric motifs in one single molecular architecture, we have previously developed a series of ferrocene-containing chalconyl–cinchona alkaloids hybrids with triazole-linkers that were found to display significant antiproliferative effects on human malignant cell lines including MDR variants [39,40]. As a continuation of this program, we envisaged the synthesis and in vitro antiproliferative evaluation of the first members of more complex triazole-tethered quinine–chalcone hybrids carrying the ferrocenoylamino group in position 9 (general type **I**, Figure 1) which were expected to allow for the disclosure of novel structure–activity relationships and the identification of potent lead compounds featuring outstanding antiproliferative activity due to their upgraded multitargeting character.

## 2. Results

### 2.1. Synthesis of the Targeted Hybrids

#### 2.1.1. Synthesis of the Azidochalcone and Aroylaminoquinine-Based Alkyne Components Used for the Click Reactions

**Scheme 1:**
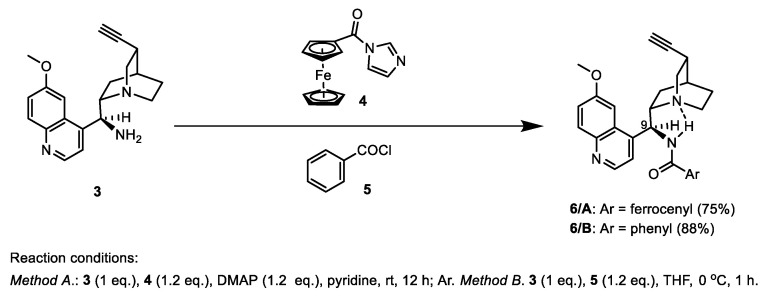
Synthesis of amides used as alkyne components in the CuAAc coupling reactions.

The azidochalcone components (**1a**–**f** and **2a**–**f**, Figure 1) intended to be used in the click reactions were obtained exploring NO_2_→N_3_ displacement in a nitrobenzaldehyde precursor, followed by the base-catalyzed enone-forming condensation of the resulting azidobenzaldehyde with the appropriate aryl-methyl ketone, as we have previously reported [32,39]. Alkyne components **6/A** and **6/B**, containing chelate-stabilized rigid amide moiety in position 9 on the quinine skeleton, were obtained in good yields by treatment of didehydroquinine-derived amine **3** [41] with activated carboxylates **4** [42] and **5** under conditions of methods A and B, respectively (Scheme 1).

#### 2.1.2. Attempted CuAAC Reactions to Construct the Targeted Hybrid Molecules

**Scheme 2:**
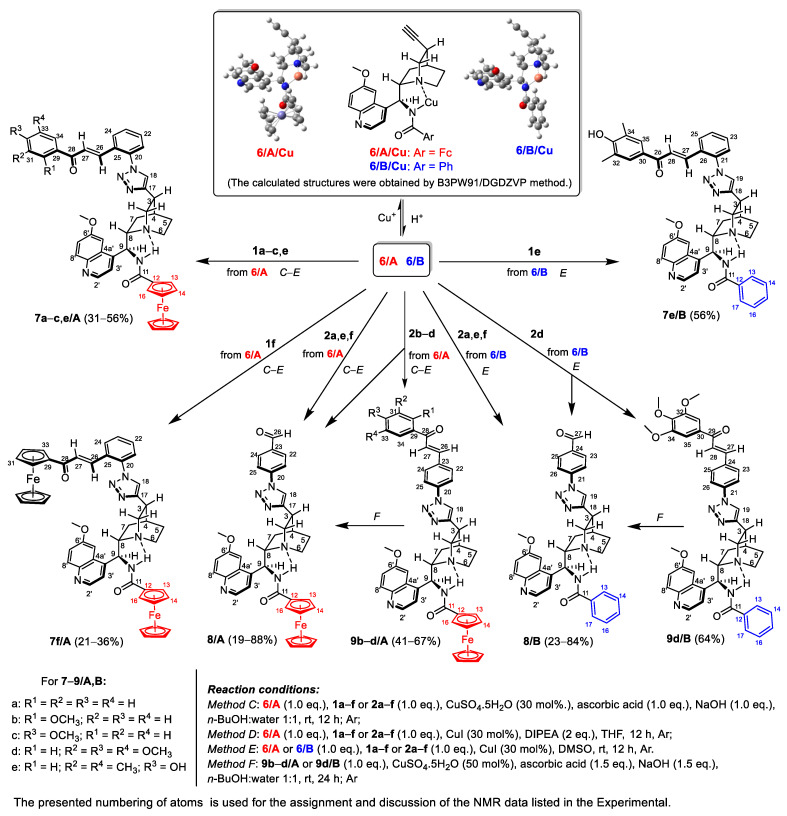
Synthesis of 9-aroylaminoquinine–chalcone hybrids tethered with 1,4-disubstituted 1,2,3-triazole linker.

The synthesis of primarily targeted 1,4-disubstituted 1,2,3-triazol-tethered hybrids carrying ferrocenoylamino units was attempted by using three protocols (methods C–E*)* of well-established copper(I)-catalyzed regioselective Sharpless [2 + 3] cycloaddition [43] involving quinine component **6/A** and the corresponding azidochalcone (**1a**–**f** and **2a**–**f**). Using high catalyst loading (0.3 eq.), irrespective of the employed conditions, the reactions with **1a**–**c**,**e**,**f** and **2b**–**d** led to the formation of the expected hybrids **7a**–**c**,**e**,**f/A** and **9b**–**d/A** (Scheme 2) in low-to-mediocre yields; however, employing **2a**,**e**,**f** as the azide component allowed for the isolation of aldehyde **8/A** as an exclusive product (Table 1).

At this stage, it is important to note that when we attempted to conduct the cycloaddition reactions with decreased loads of copper source (2–10 mol%), dramatic drops in the yields of the triazole-tethered hybrids (down to ca. 5–10%) or a practical failure of the reactions were observed, probably due to an equilibrium formation of chelate complexes **6/A/Cu** and **6/B**/**Cu** (Scheme 2) preventing a significant portion of the Cu(I) ions to be implicated as catalysts in CuAAC. Interestingly, the cycloadditions affording **9b**–**d/A** with the “para” substitution pattern in the chalcone moiety were also accompanied by the formation of **8/A** as a minor isolable product (Table 1). Azidochalcones **1e** and **2a**,**d**–**f** were also employed as coupling partners for the cyclization with benzoylamino-substituted quinine analogue **6/B** (Scheme 2) to produce benzoylamino-substituted analogues to serve as representative reference models in the cell viability assays. The reactions were exclusively conducted under the conditions of method E which—in most cases—proved to be the most efficient protocol in promoting the cycloadditions of organometallic counterpart **6/A**. Among the expected products, **7e/B** and **9d/B** were obtained in acceptable yields (56% and 64%, respectively), while the attempted reactions with **2a**,**e**,**f** resulted in aldehyde **8/B** as an exclusively isolable product in mediocre yields (Table 1). It is of note that the cycloaddition leading to triazole-linked hybrid **9d/B** was also accompanied by enone-hydrolysis, leading to **8/B** as minor product (Table 1). 

**Figure 2:**
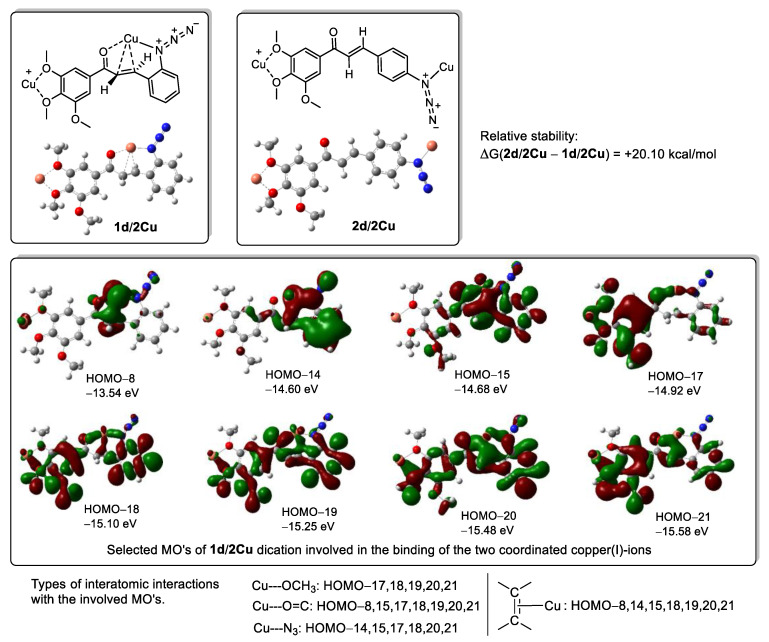
Selection of molecular orbitals involved in coordination of copper(I) ions in regioisomeric azidochalcones with chelating trimethoxyphenyl group.

It is of particular interest that under none of the applied conditions CuAAC could be achieved when ortho-azidochalcone **1d** with strongly coordinating 3,4,5-trimethoxyphenyl residue was used as coupling partner. This observation is in keeping with previous findings disclosing that strong coordinating sites can completely suppress the catalytic activity of the copper center [44]. Accordingly, as a plausible explanation of this dramatic drop in the reactivity of **1d,** it can be assumed that in addition to complexation by the methoxy groups, a substantial part of Cu(I) ions can also be removed from the catalytic process by being trapped by the enone residue and the proximal azido substituents in multi-coordinated mode to form a dicationic complex **1d**/**2Cu** with increased stability relative to its regioisomer **2d**/**2Cu** derived from the more reactive **2d** (Figure 2). This view gained support from the relative energetics of these complexes obtained from comparative DFT modeling studies carried out by the B3PW91 functional [45] using the DGDZVP basis set [46]. (We can justify our choice for the DFT functional on the basis of the fact that B3PW91 was found to be superior to B3LYP in providing a more reliable and realistic description and characterization of bonding parameters in metal-containing molecular fragments [47].) The frequency calculations performed on the optimized structures provided the free energy data unambiguously indicating that complex **1d**/**2Cu** is more stable by 20.10 kcal/mol than **2d**/**2Cu**. In line with the relative thermodynamics of these structural isomers, in addition to the MO set involved in the binding system around the Cu(I) ion chelated by two methoxy groups, MO analysis of the optimized structure of **1d**/**2Cu** identified further sets of MOs that contribute to the extra stability of this molecular architecture demonstrating significant electron delocalization over the Cu(I) center multi-coordinated by the internal azide nitrogen, the proximal carbonyl oxygen and the alkene moiety (Figure 2). It must also be pointed out that in **2d**/**2Cu** and the corresponding mono-metal complex without a methoxy-chelated Cu(I) ion, the azide-coordinated copper is not only available but necessary for the CuAAc process [48,49,50,51,52].

**Figure 3:**
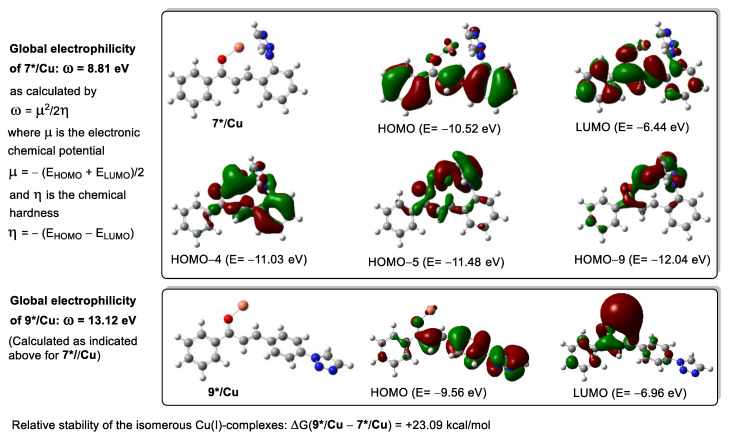
DFT reactivity indices and relevant MOs of the simplified models of enone-coordinated copper(I) complexes providing a rationale for the experimentally observed difference in the hydrolytic stability of the hybrids containing para- and ortho-disubstituted benzene ring in the central region.

Aldehydes **8/A** and **8/B** obviously resulted from the hydrolysis of the primarily formed chalcones. While examples for the aldehyde-generating hydrolytic cleavage of the C=C bond in chalcones simultaneously assisted by tetrabutylammonium hydroxide and microwave irradiation [53], promoted by secondary amines [54], immobilized Co(acac)_2_ [55] and H-phosphonates [56], have also been reported, this hydrolysis is assumed to be initiated by the copper-catalyzed conjugate addition of the water molecules present as solvent or contamination in the reaction mixtures. Accordingly, all of the isolated chalcones could be hydrolyzed into the corresponding aldehyde under the conditions of method F using a significantly increased load of copper source (50%) in the aqueous reaction mixture (Scheme 2, Table 1). The enhanced resistance of chalcone hybrids of type **7** to undergo copper-catalyzed hydrolytic fission of the C=C bond compared to hybrids of type **9** was rationalized on the basis of relative energetics, binding characteristics and reactivity indices obtained again by DFT studies on simplified models of their enone-coordinated Cu(I) complexes 7*/**Cu** and 9*/**Cu** carrying a triazolyl substituent in ortho- and para-position, respectively, of the disubstituted phenyl group (Figure 3). The substantially higher thermodynamic stability of 7*/**Cu** relative to its isomer is obviously due to the multi-coordination of the Cu(I) ion, represented by HOMO-4, HOMO-5 and HOMO-9, involving the proximal triazole ring as the donor ligand in cooperation with the carbonyl oxygen atom. In the context of hydrolytic stability, Parr’s electrophilicity index ω [57] was also calculated for both 7*/**Cu** and 9*/**Cu**, where μ is the electronic chemical potential [58] and η is the chemical hardness [59]. In the formulation of μ and η, ionization energy and electron affinity were approximated by HOMO and LUMO energy values, respectively, as presented in Figure 3. The enhanced tendency of chalcones of type **9** to undergo copper-catalyzed hydrolysis relative to chalcones of type **7** is in good accord with the electrophilicity indices 8.81 eV and 13.12 eV calculated for 7*/**Cu** and 9*/**Cu**, respectively. In this context, it is also worth underlining that the LUMO of 9*/**Cu** is significantly lower in energy than the LUMO of 7*/**Cu**.

### 2.2. Structural Elucidation of the Novel Hybrid Compounds

**Figure 4:**
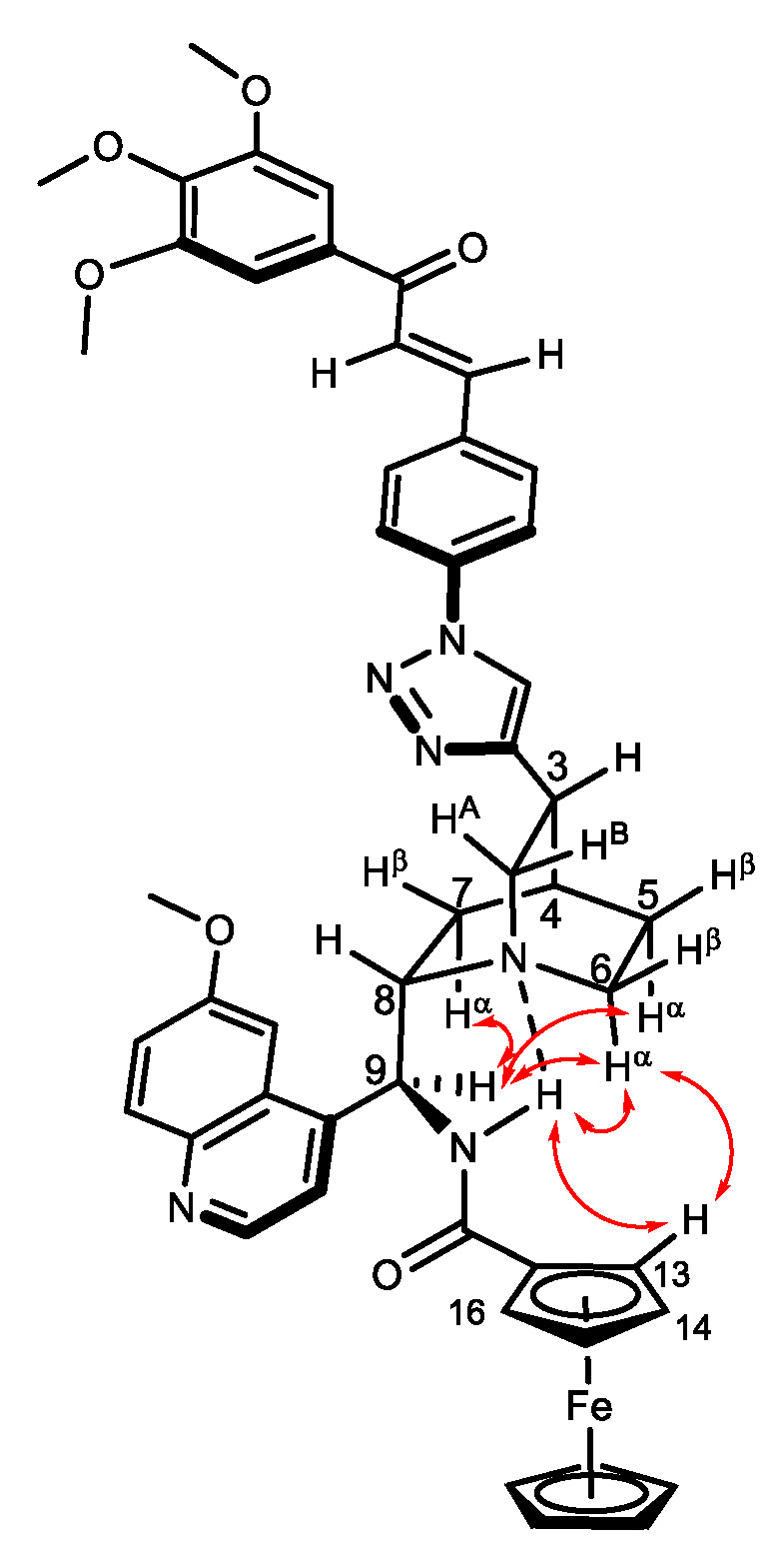
Diagnostics of NOE interactions detected in hybrid compound **9d**/**A**.

The measured ^1^H- and ^13^C-NMR data of the novel hybrid compounds listed in Materials and Methods are consistent with their structure; only the following remarks about the identification of the configuration of the C9 stereogenic center and the amide moiety are necessary to make. Indicating its endo orientation in the quinine scaffold in the representative ferrocenoylamino-substituted hybrid **9d**/**B**, proton H9 is situated in the proximity of H6α- and H7α protons, as evidenced by the NOESY correlations indicated by red arrows in Figure 4 (spectrum: cf. S18 in the Supplementary Materials). Referring to the chelate-stabilized orientation and the E-configuration of the amide moiety, further NOESY interactions were detected between the NH proton and the adjacent H6α- and H13 protons on the quinine scaffold and the ferrocenyl group, respectively. Due to the rapid rotation of the ferrocenyl group around the C11-C12 bond, NOE was also detected involving amide NH and H16 on the organometallic moiety (Figure 4). Finally, it is important to note that the highly similar ^1^H-and ^13^C chemical shifts measured for the skeletal CH- and CH_2_ groups in narrow regions of the spectra of all of the novel hybrids point to their closely related stereostructure with identical relative configuration.

### 2.3. In Vitro Antiproliferative Activities of the Novel Hybrid Compounds

The cancer cell growing inhibitory activities of some selected hybrids were determined in vitro via MTT assay against a panel of human cancer cells of gynecological origin containing HeLa (cervical cancer), MDA-MB-231 (triple negative breast cancer) and A2780 (ovarian cancer). Generally, the tested compounds exhibited lower IC_50_ values against ovarian cancer cells than cells representing breast and cervical malignancies (Table 2). The determined IC_50_ values were lower than or comparable to those of the reference agent cisplatin. Since there is no substantial difference in the cell growth inhibiting activities of **7a/A**, **7b/A**, **7c/A** and **7e/A**, the substituents on the chalcone component seem to possess limited impact on the effects of the hybrids. This finding can be confirmed by the similar properties of **9b/A** and **9d/A**, though the latter was slightly more active, especially against breast cancer cells. On the other hand, the chalcone fragment is critical for triggering the antiproliferative effect as the hybrids were eight- to ten-fold more active than aldehydes **8**/**A** and **8**/**B**. The organometallic model **9d**/**A** was identified as the most potent antiproliferative lead compound characterized by submicromolar IC_50_ values on the MDA-MB-231 and A2780 cell lines. The proliferation of the most sensitive cell line, A2780, was more efficiently inhibited by ferrocenoylamino-containing molecules **7e/A** and **9d/A** than by their benzoylamino-substituted counterparts **7e/B** and **9d**/**B**, respectively. This tendency indicates the relevance of the organometallic unit in the anticancer potency of the hybrid, which the increased lipophilicity may explain. Based on the in vitro results, the most potent agent, **9d/A**, can be considered a lead compound suitable for further development.

## 3. Materials and Methods

All fine chemicals were obtained from commercially available sources (Merck, Budapest, Hungary; Fluorochem, Graphite Way, Headfield, SK13 1QH, UK; Molar Chemicals, Halásztelek, Árpád street 1., 2314 Hungary; VWR, Debrecen, Simon László street 4., 4034 Hungary) and used without further purification. Dioxane was distilled from sodium benzophenone. Merck Kieselgel (230–400 mesh, 60 Å) was used for flash column chromatography. Melting points (uncorrected) were determined with a Büchi M-560. Elemental analyses were performed on a Vario EL-III CHN analyzer. The ^1^H- and ^13^C NMR spectra of all compounds featuring low solubility were recorded at 50 °C in DMSO-*d*_6_ solution in 5 mm tubes on a Bruker DRX-500 spectrometer at 500 (^1^H) and 125 (^13^C) MHz, with the deuterium signal of the solvent as the lock and TMS as the internal standard. The measured samples without any precipitate were prepared by heating the hardly soluble solid compounds in DMSO-*d*_6_ to reach a level of concentration allowing for the registration of spectra with a sufficient signal-to-noise ratio, which was particularly critical for the appearance of the slowly relaxing, thus significantly broadened ^1^H- and ^13^C NMR signals from the quinuclidine residue that features slow interconversion of its twisted conformations [61,62] taking place with frequency comparable to NMR time scale. It must be noted that because of the heating applied during sample preparation and the measurements, some extra signals, probably originating from the partial thermal decomposition of the measured compounds, are also discernible in the ^1^H- and ^13^C-NMR spectra collected in the Supplementary Materials. On the other hand, in addition to the sensitivity problems associated with quantitative NMR analysis of dilute solutions [63], the decreased accuracy of the integrals can also be due to a complete or partial overlap of broadened signals originating from the measured compound and the decomposition products. The 2D-HSQC-, HMBC- and NOESY spectra, which support the exact assignments of ^1^H- and ^13^C NMR signals, were registered by using the standard Bruker pulse programs. For each compound characterized in this session, the numbering of atoms used for the assignment of ^1^H- and ^13^C NMR signals does not correspond to IUPAC rules reflected from the given systematic names. All DFT calculations were carried out using a Gaussian 09 software (Gaussian Incorporation, Pittsburgh, PA, USA) package [64]. The optimized structures are available from the authors.

Azidochalcones **1a**–**f** and **2a**–**f** were synthesized by our previously reported procedures [32,39]. Didehydroquinine-derived amine **3** was accessed as trihydrochloride salt according to the reaction sequence starting for quinine [41]. Ferrocenoylimidazolide **4** was prepared using ferrocenecarboxylic acid and CDI as reactants [42].

### 3.1. Synthesis of N-((S)-((1S,2S,4S,5S)-5-Ethynylquinuclidin-2-yl)(6-methoxy-quinolin-4-yl)methyl)ferrocenecarboxamide ***6/A***

Trihydrochloride salt of **3** (2.16 g; 5 mmol, 1 eq.) and NaOH (0.64 g, 16 mmol, 3.2 eq.) were dissolved in water (15 mL). The mixture was stirred for 2 min then extracted with DCM (5 × 20 mL). The combined organic layers were dried over anhydrous Na_2_SO_4_ and evaporated to dryness on a rotary evaporator. The light yellow residue obtained by evaporation, ferrocenoylimidazolide (1.704 g; 6 mmol, 1.2 eq.) and DMAP (0.184 g; 6 mmol, 1.2 eq.) were dissolved in freshly distilled pyridine (15 mL). This reaction mixture was purged with argon and stirred for 12 h at room temperature, then poured onto crushed ice. The resulting suspension was extracted with DCM (5 × 20 mL). The combined organic layers were washed with brine solution, dried over anhydrous Na_2_SO_4_ and evaporated to dryness on a rotary evaporator. The dark solid residue was purified by column chromatography on silica using solvent mixture DCM:MeOH (20:1) as eluent, followed by sequential crystallization with water and Et_2_O to obtain the pure product as a light orange solid. Yield: 2.00 g (75%).


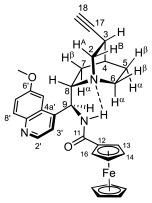


Mp: 147.9–150.0 °C (dec.); ^1^H-NMR (DMSO-d_6_): 8.72 (d, J = 4.5 Hz, 1H, H2′), 7.90 (d, J = 9.1 Hz, 1H, H8′), 7.87 (d, J = 2.5 Hz, 1H, H5′), 7.76 (d, J = 7.8 Hz, 1H, NH), 7.56 (d, J = 4.5 Hz, 1H, H3′), 7.38 (dd, J = 7 9.1 Hz and 2.5 Hz, 1H, H7′), 4.77 and 4,75 (two partly overlapping br s’s, 2H, H13 and H16), 4.28 (br s, 2H, H14 and H15), 4.04 (s, 5H, η^5^-C_5_H_5_), 5.72 (t, J = 7.8 Hz, 1H, H9), 3.95 (s, 3H, OCH_3_ on C6′), 3.60 (br s, 1H, H8), 3.28 (dd, J = 12.8 Hz and 9.9 Hz, 1H, H2A), 3.24 (br~s, 1H, H6α), 2.93 (d, J = 2.4 Hz, 1H, H18), 2.91 (br~d, J~13 Hz, 1H, H2B), 2.58 (br s, 1H, H6*β*), 2.52 (br~s, 1H, H3), 1.72 (s, 1H, H4), 1.69 (br s, 1H, H7*β*), 1.50 (br s, 1H, H5*α*), 1.40 (br s, 1H, H5*β*), and 0.78 (br s, 1H, H7*α*); ^13^C-NMR (DMSO-d_6_): 168.9 (C11), 157.8 (C6′), 148.0 (C2′), 145.7 (C4′), 144.8 (C8a’), 131.7 (C8′), 128.7 (C4a’), 121.7 (C7′), 120.8 (C3′), 103.8 (C5′), 89.8 (C17), 71.3 (C18), 76.3 (C12), 70.2 (C14/C15), 69.5 (η^5^-C_5_H_5_), 68.5 (C13 and C16), 57.9 (C8), 57.4 (OCH_3_ on C6′), 57.0 (C2), 50.4 (C9), 41.0 (C6), 27.3 (two coalesced lines, C3 and C4), 27.4 (C7), and 26.2 (C5). Anal. calcd. for C_31_H_31_FeN_3_O_2_: C, 69.80%; H, 5.86%; N, 7.88%. Found: C, 69.61%; H, 5.65%; N, 7.97%.

### 3.2. Synthesis of N-((S)-((1S,2S,4S,5S)-5-Ethynylquinuclidin-2-yl)(6-methoxyquinolin-4-yl)methyl)benzamide ***6/B***

Trihydrochloride salt of **3** (2.16 g; 5 mmol, 1 eq.) and NaOH (0.64 g, 16 mmol, 3.2 eq.) were dissolved in water (15 mL). The obtained mixture was stirred for 2 min then extracted with DCM (5 × 20 mL). The combined organic layers were dried over anhydrous Na_2_SO_4_ and evaporated to dryness on a rotary evaporator. The obtained light yellow residue was dissolved in freshly distilled THF. At 0 °C, benzoylchloride [0.7 mL (0.85 g), ~6 mmol, 1.2 eq.] and triethylamine [1.39 mL (1.012 g), 10 mmol, 2 eq.] were sequentially added to the solution of **3** in THF. The obtained reaction mixture was then stirred for 1 h and poured onto crushed ice. The resulting suspension was extracted with DCM (5 × 20 mL). The combined organic layers were washed with brine solution, dried over anhydrous Na_2_SO_4_ and evaporated to dryness on a rotary evaporator. The dark residue was purified by column chromatography on silica using solvent mixture DCM:MeOH (20:1) as eluent, followed by sequential crystallization with MeOH and Et_2_O to obtain the pure product as a pale yellow solid. Yield: 1.87 g (88%).


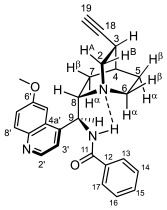


Mp: 178.6–178.9 °C; ^1^H-NMR (DMSO-d_6_): 8.78 (d, J = 4.5 Hz, 1H, H2′), 8.76 (d, J = 7.7 Hz, 1H, NH), 7.97 (d, J = 9.1 Hz, 1H, H8′), 7.89 (d, J = 2.5 Hz, 1H, H5′), 7.83 (dd, J = 7.6 Hz and 1.8 Hz, 2H, H13/H17), 7.63 (d, J = 4.5 Hz, 1H, H3′), 7.50, (tt, J = 7.6 Hz and 1.8 Hz, 1H, H15), 7.45–7.41 (overlapping m’s, 3H, H7′ and H14/H15), 5.85 (br s, 1H, H9), 3.97 (s, 3H, OCH_3_ on C6′), 3.65 (br qa, J = 9Hz, 1H, H8), 3.26 (dd, J = 12.8 Hz and 9.9 Hz, 1H, H2A), 3.24 (br ~s, 1H, H6α), 2.94 (d, J = 2.4 Hz, 1H, H19), 2.90 (br d, J = 13 Hz, 1H, H2B), 2.59 (br s, 1H, H6*β*), 2.53 (br s, 1H, H3), 1.73 (s, 1H, H4), 1.66 (br s, 1H, H7*β*), 1.54 (br s, 1H, H5*α*), 1.40 (br s, 1H, H5*β*), and 0.80 (dd, J = 12.8 Hz and 7.9 Hz, 1H, H7*α*); ^13^C-NMR (DMSO-d_6_): 166.2 (C11), 158.0 (C6′), 148.2 (C2′), 145.6 (C4′), 144.8 (C8a’), 134.7 (C15), 131.7 (C8′), 129.7 (C12), 129.0 (C4a’), 128.7 (C14/C16), 127.8 (C13/C17), 121.8 (C7′), 120.9 (C3′), 103.1 (C5′), 89.2 (C18), 71.8 (C19), 57.8 (C8), 57.4 (OCH_3_ on C6′), 57.1 (C2), 50.2 (C9), 40.7 (C6), 27.2 (two coalesced lines, C3 and C4), 27.0 (C7), and 26.2 (C5). Anal. calcd. for C_27_H_27_N_3_O_2_: C, 76.21%; H, 6.40%; N, 9.87%. Found: C, 76.05%; H, 6.23%; N, 9.76%.

### 3.3. General Procedures for the Synthesis of Quinine–Chalcone Hybrids with 1,4-Disubstituted Triazole Linkers

#### 3.3.1. Method C

N-(((2R,5R)-5-Ethynylquinuclidin-2-yl)-(6-methoxyquinolin-4-yl)methyl)ferrocenecarboxamide (**6/A**) (533 mg, 1.0 mmol, 1.0 eq.), or N-(((2R,5R)-5-ethynyl-quinuclidin-2-yl)(6-methoxy-quinolin-4-yl)methyl)benzamide (**6/B**) (425 mg, 1.0 mmol, 1.0 eq.), the corresponding azidochalchone (**1a**–**f, 2a**–**f**) (1.0 mmol, 1.0 eq.), CuSO_4_·5H_2_O (75 mg, 0.3 mmol, 0.3 eq.), NaOH (40 mg, 1.0 mmol, 1.0 eq.) and L-ascorbic acid (176 mg, 1.0 mmol, 1.0 eq.) were suspended in a mixture of water (2 mL) and n-buthanol (2 mL) under argon atmosphere. This mixture was stirred at room temperature for 12 h, then diluted with water (15 mL). The resulting suspension was extracted with DCM (5 × 25 mL). The combined organic layers were washed with brine, dried on Na_2_SO_4_ and evaporated to dryness on a rotary evaporator. The residue was subjected to flash chromatography on silica gel using mixtures of solvents DCM and MeOH (the ratio of DCM and MeOH was varied between 60:1 and 10:1). The analytical samples of the separated products were sequentially crystallized by EtOH–water and Et_2_O. The yields of the products are listed in Table 1.

#### 3.3.2. Method D

N-(((2R,5R)-5-Ethynylquinuclidin-2-yl)(6-methoxyquinolin-4-yl)methyl)ferrocenecarboxamide (**6/A**) (533 mg, 1.0 mmol, 1.0 eq.), or N-(((2R,5R)-5-ethynyl-quinuclidin-2-yl)(6-methoxyquinolin-4-yl)-methyl)benzamide (**6/B**) (425 mg, 1.0 mmol, 1.0 eq.), the corresponding azidochalchone (**1a**–**f, 2a**–**f**) (1.0 mmol, 1.0 eq.), CuI (57.2 mg, 0.3 mmol, 0.3 eq.) and DIPEA (0.35 mL. 40 mg, 2.0 mmol, 2.0 eq.) were added to THF (5 mL) under argon. The resulting mixture was stirred at room temperature for 12 h, diluted with water (25 mL) and extracted with DCM (5 × 25 mL). The combined organic layers were washed with brine, dried on Na_2_SO_4_ and evaporated to dryness on a rotary evaporator. The subsequent part of the separation and purification process was performed as given under the description of method C. The yields of the products are listed in Table 1. 

#### 3.3.3. Method E

N-(((2R,5R)-5-Ethynylquinuclidin-2-yl)(6-methoxyquinolin-4-yl)methyl)ferrocenecarboxamide (**6/A**) (533 mg, 1.0 mmol, 1.0 eq.), or N-(((2R,5R)-5-ethynyl-quinuclidin-2-yl)(6-methoxyquinolin-4-yl)-methyl)benzamide (**6/B**) (425 mg, 1.0 mmol, 1.0 eq.), the corresponding azidochalchone (**1a**–**f, 2a**–**f**) (1.0 mmol, 1.0 eq.) and CuI (57.2 mg, 0.3 mmol, 0.3 eq.) were added to DMSO (5 mL) under argon. The resulting mixture was stirred at room temperature for 12 h, diluted with a water (60 mL) and extracted with DCM (8 × 25 mL). The combined organic layers were washed with brine, dried on Na_2_SO_4_ and evaporated to dryness on a rotary evaporator. The subsequent part of the separation and purification process was performed as given under the description of method C. The yields of the products are listed in Table 1.

### 3.4. Characterization of the Products

#### 3.4.1. N-((6-Methoxyquinolin-4-yl)((2R,5S)-5-(1-(2-((E)-3-oxo-3-phenylprop-1-en-1-yl)phenyl)-1H-1,2,3-triazol-4-yl)quinuclidin-2-yl)methyl)ferrocenecarboxamide (**7a/A**)


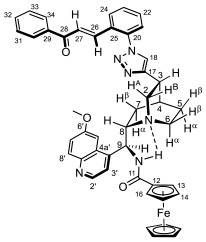


Orange solid; Mp: 134.3–137.0 °C (dec.); ^1^H-NMR (DMSO-d_6_): 8.68 (d, J = 4.5 Hz, 1H, H2′), 8.31 (s, 1H, H18), 8.25 (dd, *J* = 7.8 Hz and 2.0 Hz, 1H, H24), 7.99 (d, J = 7.5 Hz, 2H, H30/H34), 7.92 (br s, 1H, H5′), 7.90 (d, J = 9.2 Hz, 1H, H8′), 7.86 (d, J = 6.8 Hz, 1H, NH), 7.72 (d, J = 15.6 Hz, 1H, H26), 7.68–7.65 (overlapping m’s, 2H, H22 and H23), 7.55–7.59 (overlapping m’s, 3H, H21, H32 and H3′), 7.48 (t, J = 7.5 Hz, 2H, H31/H33), 7.39 (dd, J = 9.1 Hz and 2.2 Hz, 1H, H7′), 7.30 (d, J = 15.6 Hz, 1H, H27), 5.80 (br t, J = 7 Hz, 1H, H9), 4.79 and 4,78 (two partly overlapping br s’s, 2H, H13 and H16), 4.28 (br s, 2H, H14 and H15), 4.05 (s, 5H, η^5^-C_5_H_5_), 3.97 (s, 3H, OCH_3_ on C7′), 3.77 (br~s, 1H, H8), 3.46 (br s, 1H, H6α), 3.49 (br s, 2H, H2A and H2B), 3.15 (~s, overlapped by the HDO signal of the solvent, H3), 2.80 (br s, 1H, H6*β*), 1.96 (~s, 1H, H4), 1.70 (br s, 2H, H5*α* and H5*β*), 1.54 (br s, 1H, H7*β*), and 0.77 (br s, 1H, H7*α*); ^13^C-NMR (DMSO-d_6_): 189.9 (C28), 169.0 (C11), 157.8 (two coalesced lines, C4′ and C6′), 151.1 (C17), 148.0 (C2′), 144.7 (C8a’), 137.8 (two coalesced lines, C20 and C29), 137.5 (C26), 136.7 (C20), 133.6 (C32), 131.8 (C22), 131.6 (C8′), 130.6 (C23), 129.2 (C31/C33), 129.0 (C24), 128.9 (C30/C34), 128.8 (C4a’), 128.5 (C27), 127.1 (C3′), 125.8 (C25), 124.8 (C18), 121.5 (C7′), 120.8 (C21), 103.8 (C5′), 76.8 (C12), 70.3 C14/C15), 69.7 (η^5^-C_5_H_5_), 68.8 and 68.7 (C13 and C16), 58.2 (C8), 56.1 (OCH_3_ on C6′), 55.5 (C2), 50.1 (C9), 41.6 (C6), 32.9 (C3), 28.1 (C4), 27.6 (C5), and 27.1 (C7). Anal. calcd. for C_46_H_42_FeN_6_O_3_: C, 70.59%; H, 5.41%; N, 10.74%. Found: C, 70.37%; H, 5.50%; N, 10.61%.

#### 3.4.2. N-(((2S)-5-(1-(2-((E)-3-(2-Methoxyphenyl)-3-oxoprop-1-en-1-yl)phenyl)-1H-1,2,3-triazol-4-yl)-quinuclidin-2-yl)(6-methoxyquinolin-4-yl)methyl)ferrocenecarboxamide (**7b/A**)


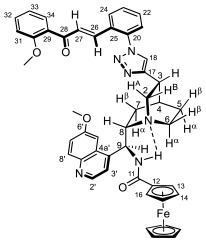


Orange solid; Mp: 147.7–150.0 °C (dec.); ^1^H-NMR (DMSO-d_6_): 8.68 (d, J = 4.5 Hz, 1H, H2′), 8.21 (s, 1H, H18), 8.06 (dd, J = 7.8 Hz and 2.0 Hz, 1H, H24), 7.93 (br s, 1H, H5′), 7.91 (d, J = 9.2 Hz, 1H, H8′), 7.84 (d, J = 6.8 Hz, 1H, NH), 7.73 (d, J = 15.6 Hz, 1H, H26), 7.65–7.62 (overlapping m’s, 2H, H22 and H23), 7.58 (d, J = 4.5 Hz, 1H, H3′), 7.51 (br d, J = 7 Hz, 1H, H21), 7.40 (dd, J = 9.2 Hz and 2.2 Hz, 1H, H7′), 7.34–7.30 (overlapping m’s, 2H, H32 and H34), 7.25 (d, J = 15.6 Hz, 1H, H27), 6.97 (d, J = 8.5 Hz, 1H, H31), 6.87 (t, J = 7.7 Hz, 1H, H33), 5.78 (br s, 1H, H9), 4.79 and 4,78 (two partly overlapping br s’s, 2H, H13 and H16), 4.23 (br s, 2H, H14 and H15), 4.06 (s, 5H, η^5^-C_5_H_5_), 3.97 (s, 3H, OCH_3_ on C7′), 3.77 (s, 3H, OCH_3_ on C30), 3.73 (br~s, 1H, H8), 3.48 (br s, 1H, H6α), 3.43 (br s, 2H, H2A and H2B), 3.08 (br s, 1H H3), 2.78 (br s, 1H, H6*β*), 1.89 (~s, 1H, H4), 1.68 (br s, 2H, H5*α* and H5*β*), 1.49 (br s, 1H, H7*β*), and 0.75 (br s, 1H, H7*α*); ^13^C-NMR (DMSO-d_6_): 192.4 (C28), 169.0 (C11), 158.2 (C30), 157.8 (two coalesced lines, C4′ and C6′), 152.0 (C17), 148.1 (C2′), 144.7 (C8a’), 137.1 (C26), 136.9 (C20), 133.5 (C34), 131.7 (C8′), 131.5 (C22), 131.2 (C25), 130.8 (C23), 129.8 (two coalesced lines C27 and C32), 128.9 (C29), 128.8 (C4a’), 128.3 (C24), 127.2 (C3′), 125.8 (C25), 124.4 (C18), 121.5 (C7′), 120.9 (C21), 103.6 (C5′), 76.8 (C12), 70.3 (C13/C14), 69.7 (η^5^-C_5_H_5_), 68.8 and 68.7 (C13 and C16), 58.4 (C8), 56.3 (OCH_3_ on C30), 56.1 (OCH_3_ on C6′), 55.8 (C2), 50.1 (C9), 41.6 (C6), 32.9 (C3), 28.2 (C4), 28.1 (C5), and 27.1 (C7). Anal. calcd. for C_47_H_44_FeN_6_O_4_: C, 69.46%; H, 5.46%; N, 10.34%. Found: C, 69.61%; H, 5.54%; N, 10.19%.

#### 3.4.3. N-(((2S)-5-(1-(2-((E)-3-(4-Methoxyphenyl)-3-oxoprop-1-en-1-yl)phenyl)-1H-1,2,3-triazol-4-yl)-quinuclidin-2-yl)(6-methoxyquinolin-4-yl)methyl)ferrocenecarboxamide (**7c/A**)


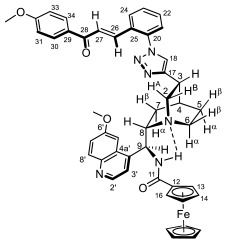


Orange solid; Mp: 155.4–155.8 °C (dec.); ^1^H-NMR (DMSO-d_6_): 8.67 (d, J = 4.5 Hz, 1H, H2′), 8.29 (s, 1H, H18), 8.25 (br ~d, J~8 Hz, 1H, H24), 8.02 (d, J = 8.4 Hz, 2H, H30/H34), 7.93 (br s, 1H, H5′), 7.90 (d, J = 9.0 Hz, 1H, H8′), 7.83 (d, J = 6.9 Hz, 1H, NH), 7.75 (d, J = 15.6 Hz, 1H, H26), 7.67–7.64 (overlapping m’s, 2H, H22 and H23), 7.56 and 7.53 (partly overlapped d and br ~d, J = 4.5 Hz and J~7Hz, resp., 2H, H3′ and H21), 7.38 (dd, J = 9.2 Hz and 2.2 Hz, 1H, H7′), 7.25 (d, J = 15.6 Hz, 1H, H27), 7.02 (d, J = 8.4 Hz, 2H, H31/H33), 5.80 (br s, 1H, H9), 4.80 and 4,77 (two br s’s, 2H, H13 and H16), 4.28 (br s, 2H, H14 and H15), 4.06 (s, 5H, η^5^-C_5_H_5_), 3.96 (s, 3H, OCH_3_ on C7′), 3.87 (s, 3H, OCH_3_ on C32), 3.74 (br ~s, 1H, H8), 3.47 (br s, 2H, H2A and H2B), 3.44 (br s, 1H, H6α), 3.13 (br s, 1H H3), 2.78 (br s, 1H, H6*β*), 1.96 (~s, 1H, H4), 1.68 (br s, 2H, H5*α* and H5*β*), 1.55 (br s, 1H, H7*β*), and 0.75 (br s, 1H, H7*α*); ^13^C-NMR (DMSO-d_6_): 187.8 (C28), 169.0 (C11), 163.9 (C32), 157.8 (two coalesced lines, C4′ and C6′), 151.2 (C17), 148.0 (C2′), 144.7 (C8a’), 137.3 (two coalesced lines, C20 and C26), 131.7 (C8′), 131.4 (two coalesced lines C22 and C30/C34), 130.5 (C23), 128.8 (two coalesced lines, C4a’ and C29), 128.6 (C24), 127.1 (C3′), 125.7 (two coalesced lines, C25 and C27), 124.8 (C18), 121.4 (C7′), 120.8 (C21), 115.2 (C31/C33), 103.8 (C5′), 76.8 (C12), 70.3 (two coalesced lines, C13 and C14), 69.7 (η^5^-C_5_H_5_), 68.8 (two coalesced lines, C13 and C16), 58.1 (C8), 56.2 (OCH_3_ on C32), 56.0 (OCH_3_ on C6′), 55.8 (C2), 50.1 (C9), 41.6 (C6), 32.9 (C3), 28.1 (C4), and 27.9 (C5), 27.2 (C7). Anal. calcd. for C_47_H_44_FeN_6_O_4_: C, 69.46%; H, 5.46%; N, 10.34%. Found: C, 69.70%; H, 5.52%; N, 10.22%.

#### 3.4.4. N-(((2S)-5-(1-(2-((E)-3-(4-Hydroxy-3,5-dimethylphenyl)-3-oxoprop-1-en-1-yl)phenyl)-1H-1,2,3-triazol-4-yl)quinuclidin-2-yl)(6-methoxyquinolin-4-yl)methyl)ferrocenecarboxamide (**7e/A**)


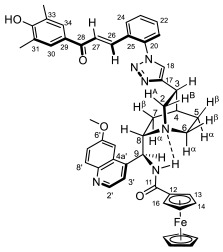


Orange solid; Mp: 172.7–173.0 °C (dec.); ^1^H-NMR (DMSO-d_6_): 9.07 (br s, 1H, OH on C32), 8.64 (d, J = 4.5 Hz, 1H, H2′), 8.30 (s, 1H, H18), 8.25 (br dd, J = 87.5 Hz and 1.5 Hz, Hz, 1H, H24), 7.88 (d, J = 9.1 Hz, 1H, H8′), 7.86 (br ~s, 1H, NH), 7.90 (d, J = 2.0 Hz, 1H, H5′), 7.76 (d, J = 15.6 Hz, 1H, (H27), 7.68 (s, 2H, H30/H34), 7.64 (td, J = 7.5 Hz and 2.0 Hz, 1H, H23), 7.60 (td, J = 7.5 Hz and 2.0 Hz, 1H, H22), 7.52 and 7.50 (partly overlapped d and br ~d, J = 4.5 Hz and J~7Hz, resp., 2H, H3′ and H21), 7.36 (dd, J = 9.2 Hz and 2.2 Hz, 1H, H7′), 7.22 (d, J = 15.6 Hz, 1H, H26), 5.80 (br s, 1H, H9), 4,77 (br s, 2H, H13 and H16), 4.26 (br s, 2H, H14 and H15), 4.01 (s, 5H, η^5^-C_5_H_5_), 3.95 (s, 3H, OCH_3_ on C7′), 3.77 (br s, 1H, H8), 3.49 (br s, 2H, H2A and H2B), 3.44 (br s, 1H, H6α), 3.10 (br s, 1H, H3), 2.79 (br s, 1H, H6*β*), 2.20 (s, 6H, CH_3_ on C31 and C33), 1.90 (~s, 1H, H4), 1.68 (br s, 2H, H5*α* and H5*β*), 1.57 (br s, 1H, H7*β*), and 0.75 (br s, 1H, H7*α*); ^13^C-NMR (DMSO-d_6_): 187.7 (C28), 169.0 (C11), 158.9 (C32), 157.8 (C6′), 150.9 (C17), 148.0 (C2′), 145.5 (C4′), 144.7 (C8a’), 137.0 (two coalesced lines, C20 and C26), 131.7 (C8′), 131.3 (C22), 131.1 (C25), 130.5 (C23), 130.1 (C30/C34), 128.9 (C4a’), 128.6 (C24), 127.1 (C3′), 126.0 (C27), 124.9 (C18), 124.7 (C31/C33), 121.5 (C7′), 120.7 (C21), 103.8 (C5′), 76.8 (C12), 70.4 (two coalesced lines, C13 and C14), 69.7 (η^5^-C_5_H_5_), 68.8 and 68.7 (C13 and C16), 58.2 (C8), 56.1 (OCH_3_ on C7′), 55.7 (C2), 50.7 (C9), 41.7 (C6), 32.8 (C3), 27.9 (C4), 27.1 (C5), 26.9 (C7), and16.9 (CH_3_ on C31 and C33). Anal. calcd. for C_48_H_46_FeN_6_O_4_: C, 69.73%; H, 5.61%; N, 10.16%. Found: C, 70.02%; H, 5.73%; N, 10.05%.

#### 3.4.5. N-((6-Methoxyquinolin-4-yl)((2R,5S)-5-(1-(2-((E)-3-oxo-3-ferrocenylprop-1-en-1-yl)phenyl)-1H-1,2,3-triazol-4-yl)quinuclidin-2-yl)methyl)ferrocenecarboxamide (**7f/A**)


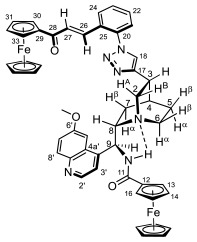


Red solid; Mp: 130.1–130.4 °C (dec.); ^1^H-NMR (DMSO-d_6_): 8.74 (d, J = 4.5 Hz, 1H, H2′), 8.44 (s, 1H, H18), 8.34 (d, J = 7.7 Hz, 1H, H24), 8.05 (d, J = 6.8 Hz, 1H, NH), 7.94 (br s, 1H, H5′), 7.92 (d, J = 9.0 Hz, 1H, H8′), 7.72 (t, J = 7.7 Hz, 1H, H23), 7.68 (t, J = 7.7 Hz, 1H, H22), 7.39 (d, J = 15.6 Hz, 1H, H26), 7.59 and 7.57 (partly overlapped d and br d, J = 4.5 Hz and J~7Hz, resp., 2H, H3′ and H21), 7.42 (dd, J = 9.2 Hz and 2.2 Hz, 1H, H7′), 7.39 (d, J = 15.6 Hz, 1H, H26), 7.25 (d, J = 15.6 Hz, 1H, H27), 5.89 (br s, 1H, H9), 4.99 (br s, 2H, H30 and H33), 4.83 (br s, 2H, H13 and H16), 4.31 (br s, 2H, H14 and H15), 4.21 (s, 5H, η^5^-C_5_H_5_ in the enone moiety), 4.06 (s, 5H, η^5^-C_5_H_5_ in the amide moiety), 3.98 (s, 3H, OCH_3_ on C7′), 3.78 (br ~s, 1H, H8), 3.51 (br s, 2H, H2A and H2B), 3.47 (br s, 1H, H6α), 3.16 (br s, 1H H3), 2.78 (br s, 1H, H6*β*), 1.99 (s, 1H, H4), 1.70 (br s, 2H, H5*α* and H5*β*), 1.65 (br s, 1H, H7*β*), and 0.78 (br s, 1H, H7*α*); ^13^C-NMR (DMSO-d_6_): 191.2 (C28), 168.9 (C11), 157.8 (two coalesced lines, C4′ and C6′), 151.1 (C17), 148.2 (C2′), 144.5 (C8a’), 136.9 (two coalesced lines, C20 and C26), 131.6 (C8′), 131.2 (two coalesced lines C22 and C23), 128.8 (two coalesced lines, C4a’ and C29), 128.6 (C24), 127.2 (C21), 127.0 (C3′), 126.2 (C27), 125.0 (C18), 121.6 (C7′), 120.5 (C25), 103.6 (C5′), 80.7 (C29), 76.8 (C12), 73.4 (two coalesced lines, C31 and C32), 70.4 (two coalesced lines, C13 and C14), 70.3 (η^5^-C_5_H_5_ in the enone moiety), 70.1 (two coalesced lines, C30 and C33), 69.7 (η^5^-C_5_H_5_ in the amide moiety), 68.7 (two coalesced lines, C13 and C16), 58.0 (C8), 55.6 (OCH_3_ on C6′), 55.8 (C2), 49.0 (C9), 41.5 (C6), 32.8 (C3), 27.8 (two coalesced lines, C4 and C5), and 27.3 (C7). Anal. calcd. for C_50_H_46_Fe_2_N_6_O_3_: C, 67.43%; H, 5.21%; N, 9.44%. Found: C, 67.29%; H, 5.33%; N, 9.53%.

#### 3.4.6. N-(((2S)-5-(1-(4-((E)-3-(2-Methoxyphenyl)-3-oxoprop-1-en-1-yl)phenyl)-1H-1,2,3-triazol-4-yl)quinuc-lidin-2-yl)(6-methoxyquinolin-4-yl)methyl)ferrocenecarboxamide (**9b/A**)


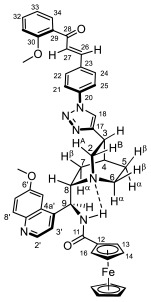


Orange solid; Mp: 172.6–173.0 °C (dec.); ^1^H-NMR (DMSO-d_6_): 8.70 (d, J = 4.5 Hz, 1H, H2′), 8.60 (s, 1H, H18), 7.93 [overlapping d (J = 16.0 Hz) and br s, 5H, H26 and H21/H22/H24/H25, resp.], 7.91 (br s, 1H, H5′), 7.90 (d, J = 9.0 Hz, 1H, H8′), 7.86 (d, J = 6.8 Hz, 1H, NH), 7.76 (d, J = 16.0 Hz, 1H, H27), 7.56–7.53 (overlapping m’s, 3H, H3′, H32, H34), 7.39 (dd, J = 9.0 Hz and 2.2 Hz, 1H, H7′), 7.19 (d, J = 8.4 Hz, 1H, H31), 7.06 (t, J = 7.5 Hz, 1H, H33), 5.87 (br s, 1H, H9), 4.79 and 4.77 (two partly overlapping br s’s, 2H, H13 and H16), 4.29 (br s, 2H, H14 and H15), 4.05 (s, 5H, η^5^-C_5_H_5_), 3.97 (s, 3H, OCH_3_ on C7′), 3.87 (s, 3H, OCH_3_ on C30), 3.71 (br s, 1H, H8), 3.46 (br s, 1H, H6α), 3.46 (br s, 2H, H2A and H2B), 3.10 (br s, 1H H3), 2.80 (br s, 1H, H6*β*), 1.89 (~s, 1H, H4), 1.70 (br s, 2H, H5*α* and H5*β*), 1.54 (br s, 1H, H7*β*), and 0.80 (br s, 1H, H7*α*); ^13^C-NMR (DMSO-d_6_): 192.5 (C28), 169.2 (C11), 158.0 (C4′), 158.4 (C30), 157.8 (C6′), 151.7 (C17), 148.0 (C2′), 144.7 (C8a’), 141.4 (C26), 138.2 (C20), 135.1 (C23), 133.5 (C34), 131.7 (C8′), 129.9 (C32), 129.4 (C29), 128.8 (C4a’), 127.5 (C3′), 121.5 (C7′), 121,1 (C33), 120.6 (two coalesced lines, C18 and C21/C25), 103.8 (C5′), 76.7 (C12), 70.4 (C13/C14), 69.7 (η^5^-C_5_H_5_), 68.8 and 68.7 (C13 and C16), 58.3 (C8), 56.5 (OCH_3_ on C30), 56.2 (OCH_3_ on C6′), 55.7 (C2), 50.9 (C9), 41.6 (C6), 32.9 (C3), 28.1 (C4), 27.7 (C5), and 27.0 (C7). Anal. calcd. for C_47_H_44_FeN_6_O_4_: C, 69.46%; H, 5.46%; N, 10.34%. Found: C, 69.30%; H, 5.31%; N, 10.53%.

#### 3.4.7. N-(((2S)-5-(1-(4-((E)-3-(4-Methoxyphenyl)-3-oxoprop-1-en-1-yl)phenyl)-1H-1,2,3-triazol-4-yl)quinuc-lidin-2-yl)(6-methoxyquinolin-4-yl)methyl)ferrocenecarboxamide (**9c/A**)


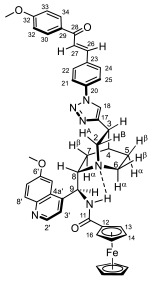


Orange solid; Mp: 185.1–165.3 °C (dec.); ^1^H-NMR (DMSO-d_6_): 8.69 (d, J = 4.5 Hz, 1H, H2′), 8.58 (s, 1H, H18), 8.14 (d, J = 8.7 Hz, 2H, H30/H34), 8.03 (d, J = 8.3 Hz, 2H, H22/H24), 7.92 [overlapping d’s (J = 16.0 Hz, 9.1 Hz and 8.3 Hz), 4H, H26, H8′ and H21/H25], 7.84 (d, J = 6.8 Hz, 1H, NH), 7.72 (d, J = 16.0 Hz, 1H, H27), 7.56 (d, J = 4.5 Hz, 1H, H3′), 7.39 (dd, J = 9.0 Hz and 2.2 Hz, 1H, H7′), 7.07 (d, J = 8.7 Hz, 2H, H32/H33), 5.77 (br ~s, 1H, H9), 4.99 and 4.97 (two partly overlapping br s’s, 2H, H13 and H16), 4.29 (br s, 2H, H14 and H15), 4.06 (s, 5H, η^5^-C_5_H_5_), 3.97 (s, 3H, OCH_3_ on C6′), 3.86 (s, 3H, OCH_3_ on C32), 3.70 (br s, 1H, H8), 3.47 (overlapping br s’s, 3H, H2A and H2B and H6α), 3.10 (br s, 1H, H3), 2.80 (br s, 1H, H6*β*), 1.99 (s, 1H, H4), 1.70 (br s, 2H, H5*α* and H5*β*), 1.54 (br s, 1H, H7*β*), and 0.80 (br s, 1H, H7*α*); ^13^C-NMR (DMSO-d_6_): 188.0 (C28), 169.0 (C11), 163.9 (C32), 157.8 (two coalesced lines, C4′ and C6′), 152.1 (C17), 148.0 (C2′), 144.6 (C8a’), 142.0 (C26), 138.2 (C20), 135.4 (C23), 131.6 (C8′), 131.0 (C29), 130.6 (C22/C24), 128.9 (C4a’), 127.2 (C3′), 123.8 (C27), 121.5 (C7′), 120.5 (two coalesced lines, C18 and C21/C25), 103.8 (C5′), 76.7 (C12), 70.5 (C13/C14), 69.7 (η^5^-C_5_H_5_), 68.8 and 68.7 (C13 and C16), 58.4 (C8), 56.5 (OCH_3_ on C30), 56.2 (OCH_3_ on C6′), 55.8 (C2), 50.3 (C9), 41.6 (C6), 33.0 (C3), 28.0 (C5), 27.8 (C4), and 27.1 (C7). Anal. calcd. for C_47_H_44_FeN_6_O_4_: C, 69.46%; H, 5.46%; N, 10.34%. Found: C, 69.25%; H, 5.36%; N, 10.50%.

#### 3.4.8. N-(((2S)-5-(1-(4-((E)-3-(3.4,5-Trimethoxyphenyl)-3-oxoprop-1-en-1-yl)phenyl)-1H-1,2,3-triazol-4-yl)-quinuclidin-2-yl)(6-methoxyquinolin-4-yl)methyl)ferrocenecarboxamide (**9d/A**)


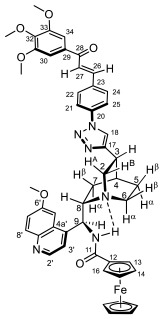


Orange solid; Mp: 205.2–205.6 °C (dec.); ^1^H-NMR (DMSO-d_6_): 8.69 (d, J = 4.5 Hz, 1H, H2′), 8.61 (s, 1H, H18), 8.07 (d, J = 8.3 Hz, 2H, H22/H24), 7.94 (d, J = 8.3 Hz, 2H, H21/H25), 7.92 (d, J = 15.6 Hz, 1H, H26), 7.91 and 7.90 (br s and d, J = 9.0 Hz, 2H, H5′ and H8′), 7.85 (d, J = 6.9 Hz, 1H, NH), 7.76 (d, J = 15.6 Hz, 1H, H27), 7.58 (d, J = 4.5 Hz, 1H, H3′), 7.46 (dd, J = 9.0 Hz and 2.0 Hz, H7′), 7.43 (s, 2H, H30/H34), 5.79 (br s, 1H, H9), 4.79 and 4.78 (two partly overlapping br s’s, 2H, H13 and H16), 4.28 (br s, 2H, H14 and H15), 4.05 (s, 5H, η^5^-C_5_H_5_), 3.96 (s, 3H, OCH_3_ on C6′), 3.90 (s, 6H, two OCH_3_ on C31 and C33), 3.78 (s, 3H, OCH_3_ on C32), 3.72 (br s, 1H, H8), 3.49 (br s, 2H, H2A and H2B), 3.46 (br s, 1H, H6α), 3.15 (br s, 1H H3), 2.80 (br s, 1H, H6*β*), 1.99 (s, 1H, H4), 1.70 (br s, 2H, H5*α* and H5*β*), 1.55 (br s, 1H, H7*β*), and 0.80 (br s, 1H, H7*α*); ^13^C-NMR (DMSO-d_6_): 188.4 (C28), 169.1 (C11), 157.8 (C6′), 153.4 (C31/C33), 151.8 (C17), 148.0 (C2′), 145.3 (C4′), 144.7 (C8a’), 142.7 (C26), 142.5 (C32), 138.2 (C20), 135.2 (C23), 131.7 (C8′), 133.4 (C29), 130.8 (C22/C24), 128.8 (C4a’), 127.6 (C3′), 123.7 (C27), 121.5 (C7′), 120.5 (two coalesced lines, C18 and C21/C25), 103.8 (C5′), 76.8 (C12), 70.4 (C13/C14), 69.7 (η^5^-C_5_H_5_), 68.8 and 68.7 (C13 and C16), 60.7 (OCH_3_ on C32), 58.4 (two coalesced lines, OCH_3_ on 6′ and C8), 57.0 (two OCH_3_ on C31 and C33), 55.9 (C2), 50.2 (C9), 41.7 (C6), 32.9 (C3), 28.0 (C5), 27.8 (C4), and 27.1 (C7). Anal. calcd. for C_49_H_48_FeN_6_O_6_: C, 67.43%; H, 5.54%; N, 9.63%. Found: C, 69.57%; H, 5.61%; N, 9.71%.

#### 3.4.9. N-(((2S)-5-(1-(2-((E)-3-(4-Hydroxy-3,5-dimethylphenyl)-3-oxoprop-1-en-1-yl)phenyl)-1H-1,2,3-triazol-4-yl)quinuclidin-2-yl)(6-methoxyquinolin-4-yl)methyl)benzamide (**7e/B**)


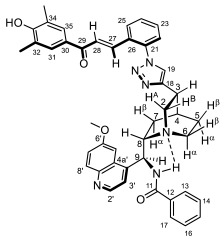


Light yellow solid; Mp: 185.2–185.4 °C (dec.); (DMSO-d_6_): 8.63 (d, J = 4.5 Hz, 1H, H2′), 8.53 (d, J = 7.3 Hz, 1H, NH), 8.22 (s, 1H, H19), 8.21 (br dd, J = 87.5 Hz and 1.5 Hz, Hz, 1H, H25), 7.90 (d, J = 2.0 Hz, 1H, H5′), 7.89 (d, J = 9.1 Hz, 1H, H8′), 7.79 (dd, J = 7.6 Hz and 1.8 Hz, 2H, H13/H17), 7.69 (d, J = 15.6 Hz, 1H, H28), 7.64 (s, 2H, H31/H35), 7.63 (td, J = 7.5 Hz and 2.0 Hz, 1H, H24), 7.59 (td, J = 7.5 Hz and 2.0 Hz, 1H, H23), 7.55 and 7.53 [partly overlapping d (J = 4.5 Hz) and tt (J = 7.6 Hz and 1.8 Hz), 2H, H3′ and H15], 7.49 (dd, J = 7.5 Hz and 2.0 Hz, 1H, H22), 7.38 (t, J = 7.6 Hz, 2H, H14/H16), 7.36 (dd, J = 9.2 Hz and 2.2 Hz, 1H, H7′), 7.20 (d, J = 15.6 Hz, 1H, H27), 5.82 (dd, J = 11.0 Hz and 7.3 Hz, 1H, H9), 3.93 (s, 3H, OCH_3_ on C7′), 3.72 (dd, J = 11.0 Hz, and 7.3 Hz, 1H, H8), 3.44 (br s, 2H, H2A and H2B), 3.38 (br s, 1H, H6α), 3.10 (br s, 1H, H3), 2.71 (m, 1H, H6*β*), 2.20 (s, 6H, CH_3_ on C32 and C34), 1.91 (~s, 1H, H4), 1.65 (br s, 2H, H5*α* and H5*β*), 1.47 (br s, 1H, H7*β*), and 0.75 (dd, J = 13.2 Hz and 7.8 Hz, 1H, H7*α*); ^13^C-NMR (DMSO-d_6_): 187.8 (C29), 166.4 (C11), 158.9 (C33), 157.9 (C6′), 151.1 (C18), 148.1 (C2′), 145.5 (C4′), 144.6 (C8a’), 137.0 (two coalesced lines, C21 and C27), 135.0 (C12), 133.0 (C15), 131.7 (C8′), 131.2 (C23), 131.1 (C26), 130.4 (C24), 130.0 (C31/C35), 128.9 (C4a’), 128.6 (two coalesced lines, C14/C16 and C25), 127.8 (C13/C17), 127.3 (C30), 127.1 (C22), 126.0 (C28), 124.8 (C32/C34), 124.7 (C19), 121.5 (C7′), 120.7 (C3′), 103.8 (C5′), 58.5 (C8), 56.1 (OCH_3_ on C7′), 55.8 (C2), 50.9 (C9), 41.4 (C6), 32.8 (C3), 27.1 (two coalesced lines, C4 and C5), 26.9 (C7), and 16.9 (CH_3_ on C32 and C34). Anal. calcd. for C_44_H_42_N_6_O_4_: C, 73.52%; H, 5.89%; N, 11.69%. Found: C, 73.62%; H, 5.75%; N, 11.56%.

#### 3.4.10. N-(((2S)-5-(1-(4-((E)-3-(3.4,5-Trimethoxyphenyl)-3-oxoprop-1-en-1-yl)phenyl)-1H-1,2,3-triazol-4-yl)-quinuclidin-2-yl)(6-methoxyquinolin-4-yl)methyl)benzamide (**9d/B**)


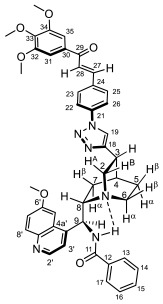


Pale yellow solid; Mp: 217.7–219.1 °C (dec.); ^1^H-NMR (DMSO-d_6_): 8.83 (~d, J~6 Hz, 1H, NH), 8.73 (s, 1H, H19), 8.72 (d, J = 4.5 Hz, 1H, H2′), 8.14 (d, J = 8.3 Hz, 2H, H23/H25), 8.04 (d, J = 15.6 Hz, 1H, H28), 7.99 (d J = 8.3 Hz, 2H, H22/H26), 7.81 (d, J = 15.6 Hz, 1H, H27), 7.94 (d, J = 9.1 Hz, 1H, H8′), 7.90 (br s 1H, H5′), 7.86 (dd, J = 7.6 Hz and 1.8 Hz, 2H, H13/H17), 7.61 (d, J = 4.5 Hz, 1H, H3′), 7.51, (tt, J = 7.6 Hz and 1.8 Hz, 1H, H15), 7.47 (s, 2H, H31/H35), 7.44 and 7.42 [partly overlapping dd (J = 9.0 Hz and 2.0 Hz) and td (J = 7.6 Hz and 1.8 Hz), 3H, H7′ and H14/H16], 5.90 (br ~s, 1H, H9), 3.97 (s, 3H, OCH_3_ on C6′), 3.92 (s, 6H, two OCH_3_ on C32 and C34), 3.78 (s, 3H, OCH_3_ on C33), 3.75 (br ~s, 1H, H8), 3.50 (br s, 1H, H2A), 3.42 (overlapping br s’s, 2H, H2B and H6α), 3.11 (br s, 1H H3), 2.79 (br s, 1H, H6*β*), 1.99 (s, 1H, H4), 1.70 (br s, 2H, H5*α* and H5*β*), 1.54 (br s, 1H, H7*β*), and 0.81 (br s, 1H, H7*α*); ^13^C-NMR (DMSO-d_6_): 188.5 (C29), 166.5 (C11), 157.9 (C6′), 153.4 (C32/C34), 151.9 (C18), 148.1 (C2′), 145.0 (C4′), 144.7 (C8a’), 142.9 (C27), 142.6 (C33), 138.2 (C21), 135.1 (C24), 131.7 (C8′), 133.2 (C30), 130.7 (C23/C25), 129.0 (C4a’), 127.6 (C3′), 123.2 (C28), 121.8 (C7′), 120.4 (two coalesced lines, C19 and C22/C26), 103.0 (C5′), 60.7 (OCH_3_ on C33), 58.4 (C8), 56.1 (OCH_3_ on 6′), 56.7 (two OCH_3_ on C32 and C34), 55.6 (C2), 50.0 (C9), 41.3 (C6), 32.7 (C3), 27.7 (C5), 27.5 (C4), and 26.7 (C7). Anal. calcd. for C_45_H_44_N_6_O_6_: C, 70.66%; H, 5.80%; N, 10.99%. Found: C, 70.52%; H, 5.71%; N, 10.81%.

#### 3.4.11. N-(((2R,5S)-5-(1-(4-Formylphenyl)-1H-1,2,3-triazol-4-yl)quinuclidin-2-yl)(6-methoxyquinolin-4-yl)-methyl)ferrocenecarboxamide (**8/A**)


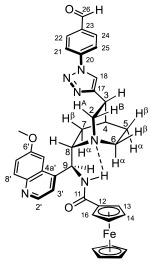


Deep orange solid; Mp: 174.6–175.0 °C (dec.); ^1^H-NMR (DMSO-d_6_): 10.08 (H26), 8.84 (s, 1H, H18), 8.72 (d, J = 4.5 Hz, 1H, H2′), 8.17 (d, J = 8.8 Hz, 2H, H22/H24), 8.05 (br s, 1H, NH), 8.03 (d J = 8.8 Hz, 2H, H21/H25), 7.93 and 7.92 (partly overlapping d’s, J = 2.0 Hz and 9.0 Hz, respectively, 2H, H5′ and H8′), 7.56 (d, J = 4.5 Hz, 1H, H3′), 7.41 (dd, J = 9.0 Hz and 2.0 Hz, H7′), 5.87 (br s, 1h, H9), 4.83 (br s, 2H, H13 and H16), 4.32 (t, J = 2 Hz, 2H, H14 and H15), 4.05 (s, 5H, η^5^-C_5_H_5_), 5.84 (br s, 1H, H9), 3.98 (s, 3H, OCH_3_ on C6′), 3.74 (br s, 1H, H8), 3.53–3.40 (overlapping br s’s, 3H, H6α, H2A and H2B), 3.13 (br s, 1H H3), 2.80 (br s, 1H, H6*β*), 2.00 (s, 1H, H4), 1.71 (br s, 2H, H5*α* and H5*β*), 1.60 (br s, 1H, H7*β*), and 0.79 (br s, 1H, H7*α*); ^13^C-NMR (DMSO-d_6_): 192.6 (C26), 168.9 (C11), 157.7 (C6′), 152.0 (C17), 148.1 (C2′), 145.5 (C4′), 144.6 (C8a’), 141.1 (C20), 135.9 (C23), 131.9 (C22/C24), 131.7 (C8′), 128.7 (C4a’), 127.6 (C3′), 121.7 (C7′), 120.9 (C18), 120.5 (C21/C25), 103.5 (C5′), 76.6 (C12), 70.4 (C13/C14), 69.7 (η^5^-C_5_H_5_), 68.9 and 68.7 (C13 and C16), 58.2 (C8), 56.1 (OCH_3_ on 6′), 55.4 (C2), 49.3 (C9), 41.6 (C6), 32.7 (C3), 27.9 (C4), 27.6 (C5), and 27.1 (C7). Anal. calcd. for C_38_H_36_FeN_6_O_3_: C, 67.06%; H, 5.33%; N, 12.35%. Found: C, 66.98%; H, 5.41%; N, 12.22%.

#### 3.4.12. N-(((2R,5S)-5-(1-(4-Formylphenyl)-1H-1,2,3-triazol-4-yl)quinuclidin-2-yl)(6-methoxyquinolin-4-yl)-methyl)benzamide (**8/B**)


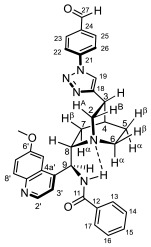


Yellow solid; Mp: 191.3–191.7 °C; ^1^H-NMR (DMSO-d_6_): 10.03 (H27), 8.68 (s, 1H, H19), 8.66 (d, J = 4.5 Hz, 1H, H2′), 8.08 and 8.06 (A and B part of an AA’BB’ spin system, J_AB_ = 9.0 Hz, 4H, H22/H26 and H23/H25), 8.00 (br s, 1H, NH), 7.89 (d, J = 2.0 Hz, 1H, H5′), 7.80 (dd, J = 7.6 Hz and 1.8 Hz, 2H, H13/H17), 7.57(d, J = 4.5 Hz, 1H, H3′), 7.46 (tt, J = 7.6 Hz and 1.8 Hz, 1H, H15), 7.39 (t, J = 7.6 Hz, 2H, H14/H16), 7.37 (dd, J = 9.0 Hz and 2.0 Hz, H7′), 5.66 (br s, 1H, H9), 3.94 (s, 3H, OCH_3_ on C6′), 3.74 (br ~s, 1H, H8), 3.53–3.40 (overlapping br s’s, 3H, H6α, H2A and H2B), 3.10 (br s, 1H H3), 2.80 (br s, 1H, H6*β*), 1.96 (s, 1H, H4), 1.69 (br s, 2H, H5*α* and H5*β*), 1.49 (br s, 1H, H7*β*), and 0.80 (br s, 1H, H7*α*); ^13^C-NMR (DMSO-d_6_): 192.9 (C27), 166.4 (C11), 157.9 (C6′), 151.9 (C18), 148.0 (C2′), 145.5 (C4′), 144.6 (C8a’), 141.3 (C21), 136.1 (C24), 134.9 (12), 131.7 (two coalesced lines, C8′ and C23/C25), 128.7 (C4a’), 127.5 (C3′), 121.7 (C7′), 120.9 (C19), 120.6 (C22/C26), 103.4 (C5′), 58.7 (C8), 56.2 (OCH_3_ on 6′), 55.6 (C2), 50.1 (C9), 41.5 (C6), 32.7 (C3), 27.7 (C5), 27.6 (C4), and 26.7 (C7). Anal. calcd. for C_34_H_32_N_6_O_3_: C, 71.31%; H, 5.63%; N, 14.68%. Found: C, 71.48%; H, 551%; N, 14.52%.

### 3.5. Determination of Antiproliferative Activities

The antiproliferative properties of a selected set of the prepared quinine–chalcone hybrids were determined by the standard MTT [3-(4,5-dimethylthiazol-2-yl)-2,5-diphenyltetrazolium bromide] method against a panel of human cancer cell lines of gynecological origin [65]. HeLa, MDA-MB-231 and A2780 cell lines isolated from cervical, breast and ovarian cancer, respectively, were obtained from the European Collection of Cell Cultures (Salisbury, UK). The cells were maintained in Eagle’s minimal essential medium supplemented with 10% fetal calf serum, 1% non-essential amino acids and 1% antibiotic–antimycotic at 37 °C in a humidified atmosphere with 5% CO_2_. Media and supplements were purchased from Lonza Group Ltd. (Basel, Switzerland). Malignant cells were plated into 96-well plates at 5000 cells/well density. After overnight incubation, the test compounds were added at six different concentrations (0.10–30 μM) and incubated for 72 h under cell-culturing conditions. Finally, the MTT solution was added to each well (20 μL of 5 mg/mL), and the medium was removed after a 4 h incubation period. The precipitated formazan crystals were solubilized in 100 μM dimethylsulfoxide through shaking at 37 °C for 60 min. The absorbance was measured at 545 nm using a microplate reader (BMG Labtech, Ortenberg, Germany). Cisplatin (Ebewe Pharma GmbH, Unterach, Austria), a clinically used anticancer drug, was applied as a reference compound. Two independent experiments were performed with five parallel wells. The IC_50_ values were calculated by fitting sigmoid concentration–response curves using GraphPad Prism 10.0 software (GraphPad Software, San Diego, CA, USA).

## 4. Conclusions

Employing copper-catalyzed azide–alkyne cycloadditions (CuAAC), we prepared a series of novel hybrids comprising triazole-linked ferrocenoylamino-substituted quinine and chalcone moieties. Despite the general robust “click” character of CuAAC processes, the target products could only be obtained by unusually high catalyst loadings referring to coordination modes that trap a substantial portion of the Cu(I) ions, preventing them from entering the ring-forming catalytic cycle, as it was suggested by the DFT modeling studies. The CuAAC reactions leading to hybrids with a para-disubstituted benzene ring in the terminal chalcone residue were accompanied by copper-catalyzed hydrolytic cleavage of the enone C=C bond. The resistance to hydrolysis of the isomers with an ortho-disubstituted benzene ring in the same skeletal region was also rationalized by comparative DFT analysis of the appropriate copper-activated complexes. 

The evaluation of the novel hybrids for their antiproliferative activity on Hela (cervical cancer cells), MDA-MB-231 (triple negative breast cancer cells) and A2780 (ovarian cancer cells) demonstrated their highly promising potency in anticancer therapy. The model carrying a terminal 3,4,5-trimethoxyphenyl-substituted ring on the chalcone subunit with a central para-disubstituted benzene ring and ferrocenoylamino group in position 9 of the quinine fragment (**9d**/**A**) was identified as the lead, displaying substantial submicromolar activity on MDA-MB-231 and A2780 cell lines. The arrangement of building blocks and other structural characteristics of this lead might serve as a good directive for the development of more potent drug candidates in related families of hybrid compounds.

## Figures and Tables

**Table 1:** Yields of the products (%) obtained from CuAAc (methods C/D/E) and copper-catalyzed hydrolysis reactions (method F) (the reactions are presented in Scheme 2).

Product	Method C	Method D	Method E	Method F
**7a/A**	43	52	37	-
**7b/A**	56	52	45	-
**7c/A**	30	52	56	-
**7e/A**	31	36	33	-
**7f/A**	21	30	36	-
**8/A**	71 (from **6**/**A**+**2a**) 28 (from **6**/**A**+**2b**) 31 (from **6**/**A**+**2c**) 34 (from **6**/**A**+**2d**) 63 (from **6**/**A**+**2e**) 57 (from **6**/**A**+**2f**)	68 (from **6**/**A**+**2a**) 22 (from **6**/**A**+**2b**) 19 (from **6**/**A**+**2c**) 32 (from **6**/**A**+**2d**) 52 (from **6**/**A**+**2e**) 42 (from **6**/**A**+**2f**)	59 (from **6**/**A**+**2a**) 20 (from **6**/**A**+**2b**) 24 (from **6**/**A**+**2c**) 20 (from **6**/**A**+**2d**) 57 (from **6**/**A**+**2e**) 45 (from **6**/**A**+**2f**)	80 (from **9b**/**A**) 74 (from **9c**/**A**) 88 (from **6d**/**A**) -
**9b/A**	47	51	63	-
**9c/A**	42	55	60	-
**9d/A**	49	62	67	-
**7e/B**	-	-	56	-
**8b/B**	-	-	77 (from **6**/**B**+**2a**) 23 (from **6**/**B**+**2d**) 69 (from **6**/**B**+**2e**) 47 (from **6**/**B**+**2f**)	84
**9d/B**	-	-	64	-

Reaction conditions: method C: **6**/**A** (1.0 eq.), **1a**-**f** or **2a**-**f** (1.0 eq.), CuSO_4_.5H_2_O (30 mol%.), ascorbic acid (1.0 eq.), NaOH (1.0 eq.), n-BuOH:water 1:1, rt, 12 h, Ar; method D: **6**/**A** (1.0 eq.), **1a**-**f** or **2a**-**f** (1.0 eq.), CuI (30 mol%), DIPEA (2 eq.), THF, 12 h, Ar; method E: **6**/**A** or **6**/**B** (1.0 eq.), **1a**-**f** or **2a**-**f** (1.0 eq.), CuI (30 mol%), DMSO, rt, 12 h, Ar; method F: **9b**-**d**/**A** or **9d**/**B** (1.0 eq.), CuSO_4_.5H_2_O (50 mol%), ascorbic acid (1.5 eq.), NaOH (1.5 eq.), n-BuOH:water 1:1, rt, 24 h, Ar.

**Table 2:** Antiproliferative activities of novel aroylamino-substituted cinchona–chalcone hybrids expressed in IC_50_ values on three human tumor cell lines.

	IC50 (µM) [95% Confidence Interval]		
	HeLa	MDA-MB-231	A2780
**7a**/**A**	2.58 [2.26–2.94]	1.55 [1.39–1.71]	0.944 [0.866–1.03]
**7b**/**A**	2.17 [1.90–2.47]	1.80 [1.58–2.05]	1.19 [1.05–1.34]
**7c**/**A**	4.18 [3.69–4.75]	2.07 [1.58–2.72]	1.51 [1.34–1.71]
**7e**/**A**	4.87 [4.34–5.42]	2.69 [2.46–2.95]	2.56 [2.31–2.84]
**8**/**A**	18.51 [13.70–24.99]	17.80 [12.50–25.37]	11.28 [9.28–13.72]
**9b**/**A**	4.86 [4.20–5.61]	3.44 [2.69–4.39]	1.10 [0.949–1.27]
**9d**/**A**	2.97 [2.56–3.44]	0.854 [0.752–0.950]	0.438 [0.401–0.579]
**7e**/**B**	4.79 [4.18–5.49]	2.22 [1.97–2.50]	1.04 [0.945–1.14]
**8**/**B**	17.60 [15.03–20.61]	16.14 [14.09–18.48]	12.75 [11.09–14.65]
**9d**/**B**	2.12 [1.71–2.62]	1.71 [1.53–1.91]	0.776 [0.652–0.924]
**Cisplatin** *	14.02 [12.65–15.56]	18.65 [16.67–20.85]	5.27 [4.37–6.35]

* Used as reference agents. Data for HeLa and MDA-MB-231 cells are from reference [60].

## Data Availability

The data generated and analyzed during our research are not available in any public database or repository but will be shared by the corresponding author upon reasonable request.

## Supplementary Materials

The following supporting information can be downloaded at: https://www.mdpi.com/article/10.3390/molecules29020375/s1, Copies of the 1D- and 2D-NMR spectra of the novel alkyne precursors S2–S4; Copies of the 1D- and 2D-NMR spectra of the targeted hybrid compounds S5–S25.
