# Supplementary material for: CuAAC-Based Synthesis, Copper-Catalyzed Aldehyde-Forming Hydrolytic Fission and Antiproliferative Evaluation of Novel Ferrocenoylamino-Substituted Triazole-Tethered Quinine–Chalcone Hybrids
Source: Molecules. 2024 Jan 11;29(2):375. doi: 10.3390/molecules29020375 (PMC10820026; doi:10.3390/molecules29020375)

CuAAC-Based Synthesis, Copper-Catalyzed Aldehyde-Forming Hydrolytic Fission and Antiproliferative Evaluation of Novel Ferrocenoylamino-Substituted Triazole-Tethered Quinine–Chalcone Hybrids

António Dembo [1,2], Etelka Ferenczi [1,2], Tamás Jernei [1], Andrea Bor [2], Zsuzsanna Schelz [2], István Zupkó [2], Szilárd Varga [3] and Antal Csámpai [1,*]

[1] Department of Organic Chemistry, Eötvös Loránd University (ELTE), Pázmány P. sétány 1/A, H-1117 Budapest, Hungary; diandjungudembo2@gmail.com (A.D.); etelkaferenczi@gmail.com (E.F.); tamas.jernei@ttk.elte.hu (T.J.)

[2] Hevesy György PhD School of Chemistry, Pázmány P. sétány 1/A, H-1117 Budapest, Hungary; bor.gytk@gmail.com (A.B.); schelz.zsuzsanna@szte.hu (Z.S.); zupko.istvan@szte.hu (I.Z.)

[3] Institute of Pharmacodynamics and Biopharmacy, University of Szeged, Eötvös u. 6., H-6720 Szeged, Hungary; varga.szilard@ttk.hu

[4] HUN-REN Research Centre for Natural Sciences, Institute of Organic Chemistry, Magyar Tudósok Krt 2., H-1117 Budapest, Hungary

* Correspondence: antal.csampai@ttk.elte.hu; Tel.: +36-01-372-2500 (ext. 6591)

Supporting Information

Table of content

1 Copies of NMR spectra 2

1.1 NMR spectra of novel alkyne precursors 2

1.2 NMR spectra of the novel hybrid compounds 5

1 Copies of NMR spectra

1.1 NMR spectra of the novel alkyne precursors

^{1}H-NMR of **6**/**A** (500 MHz, DMSO-d$_6$)

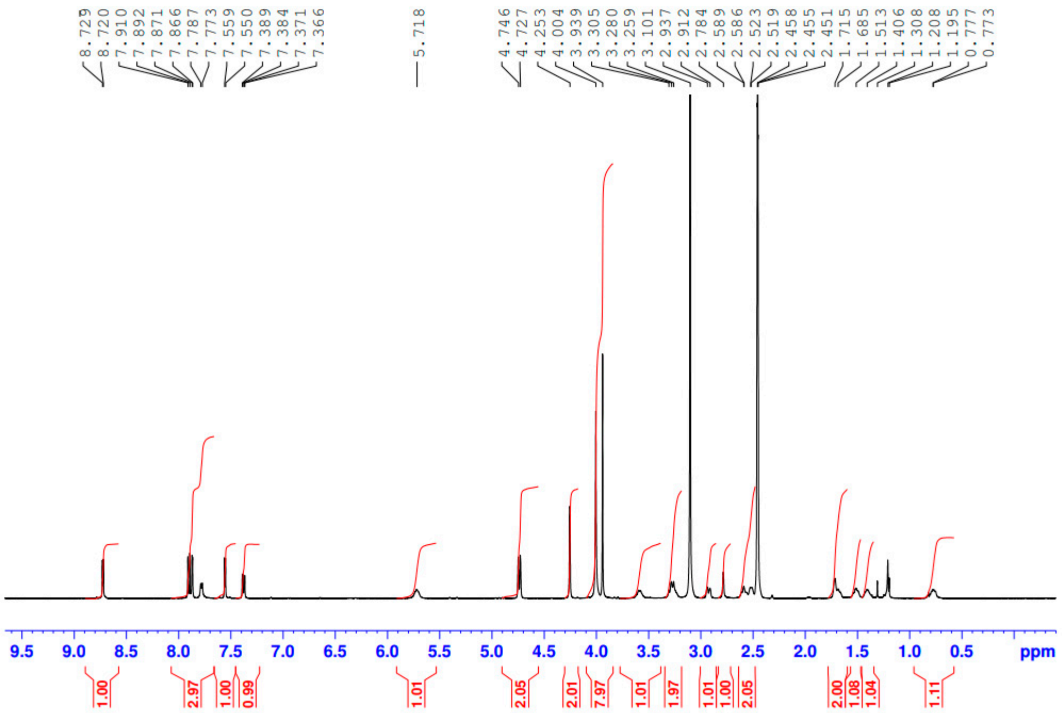

^{13}C-NMR of **6**/**A** (125 MHz, DMSO-d$_6$)

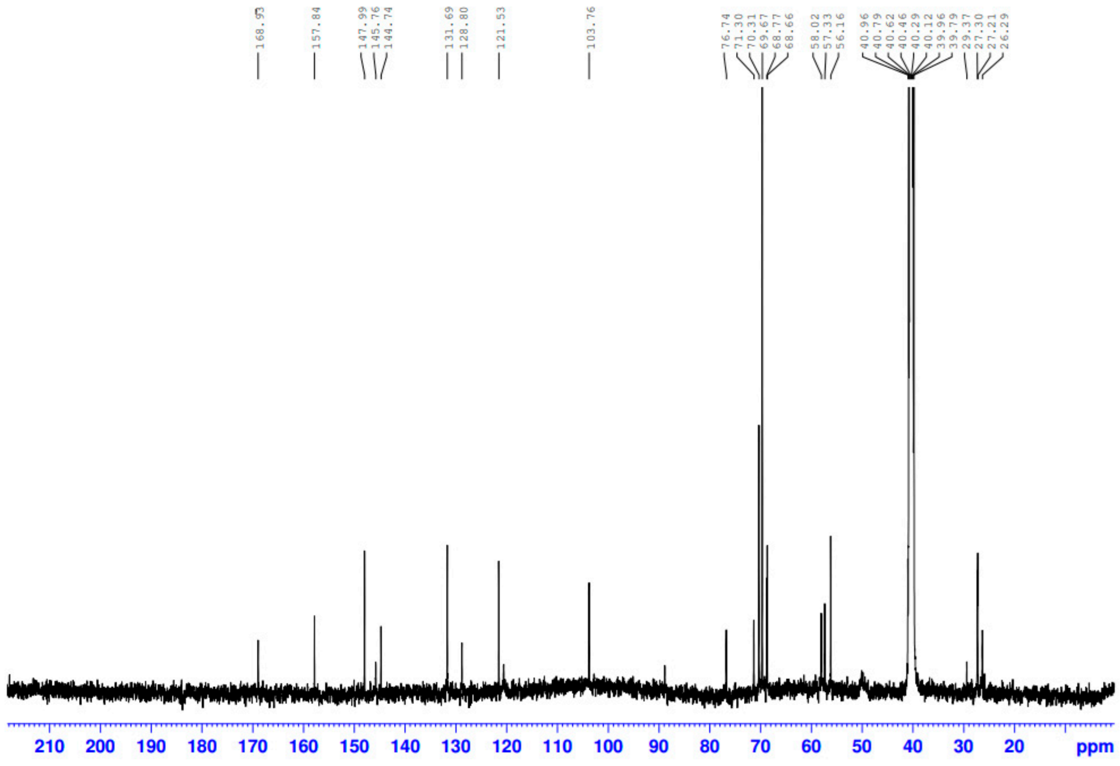

^{1}H-^{13}C-HSQC of **6**/**A**

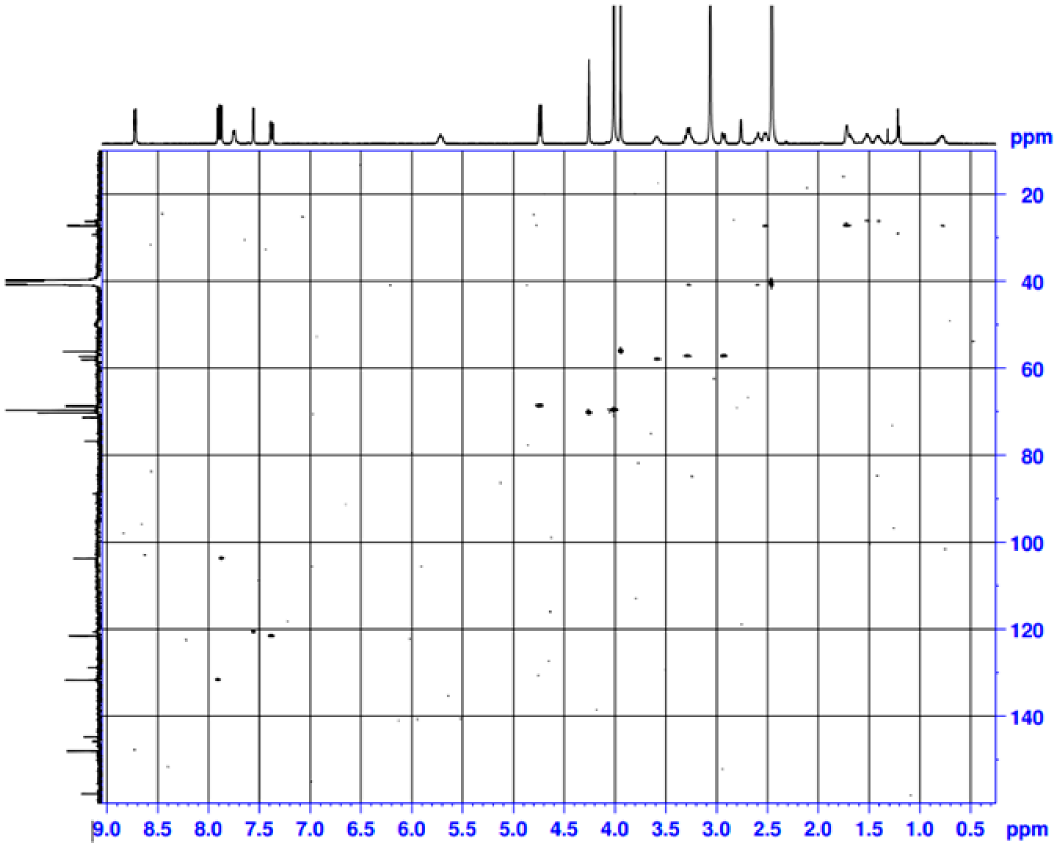

^{1}H-NMR of **6**/**B** (500 MHz, DMSO-d_6)

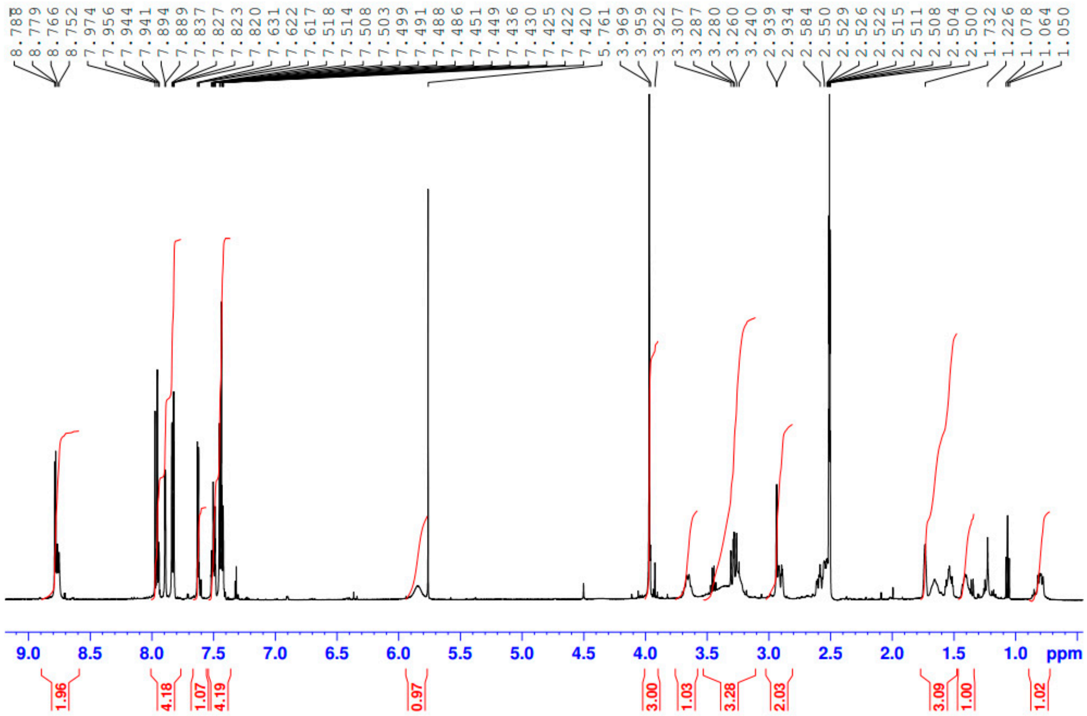

^{13}C-NMR of **6**/**B** (125 MHz, DMSO-d$_6$)

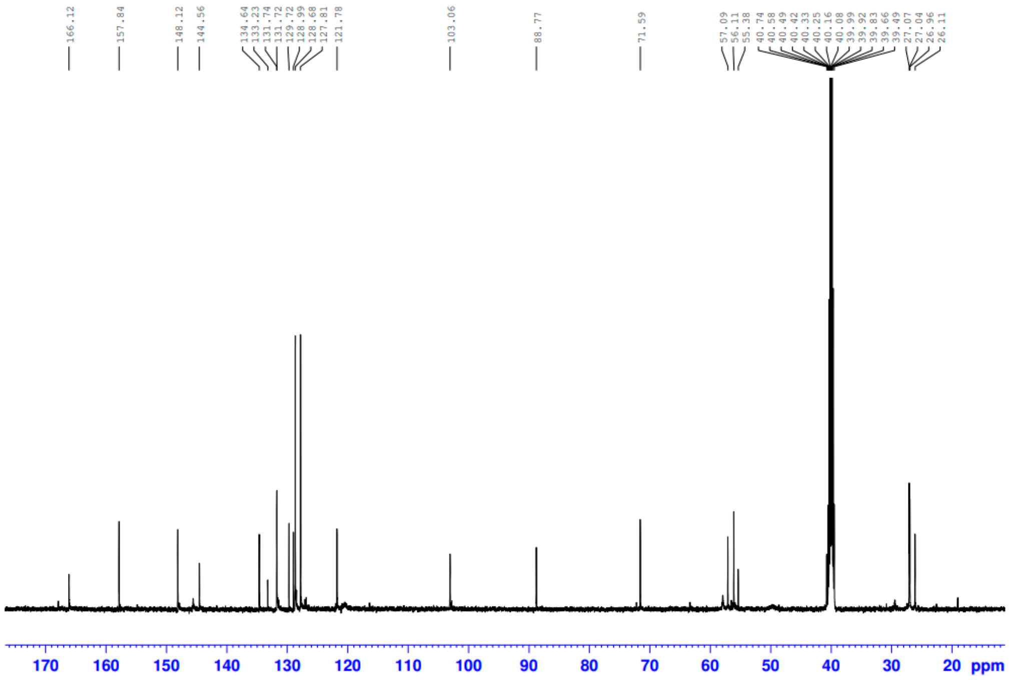

^{1}H-^{13}C-HSQC of **6**/**B**

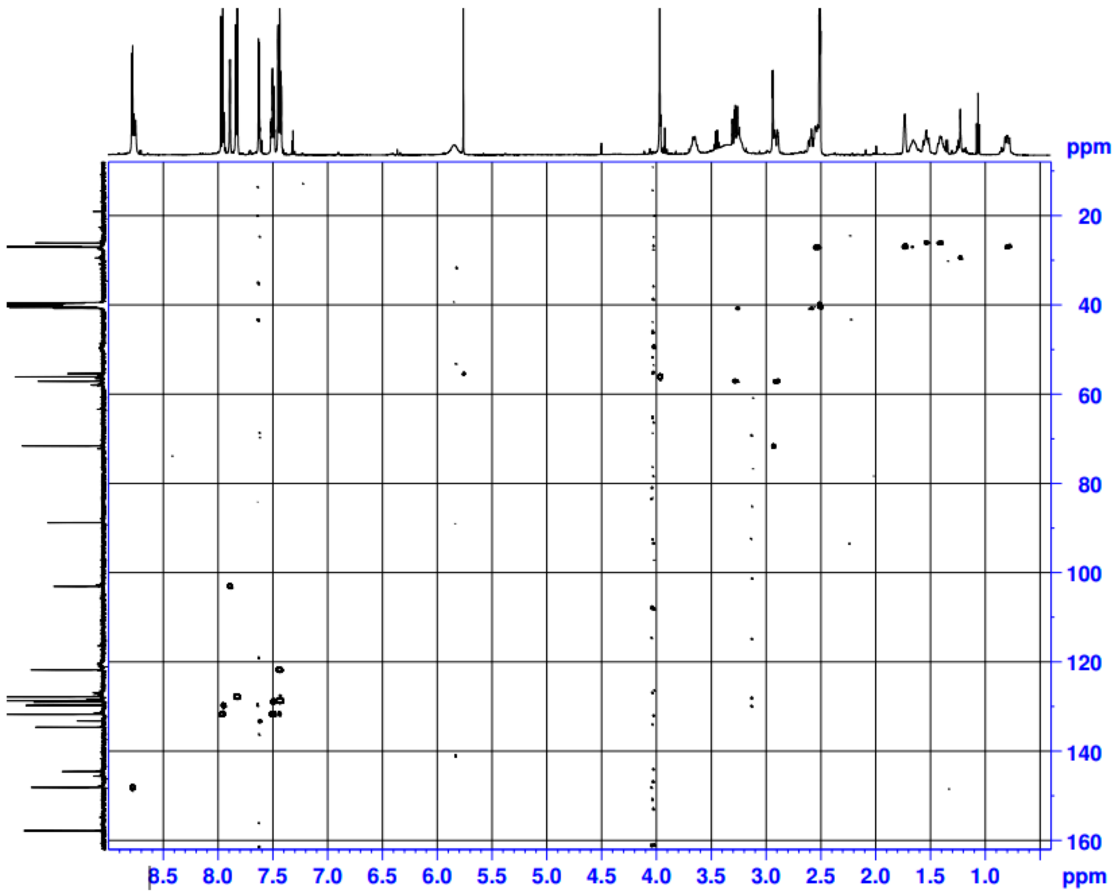

1.2 NMR spectra of the targeted hybrid compounds

^{1}H-NMR of **7a/A** (500 MHz, DMSO-d6)

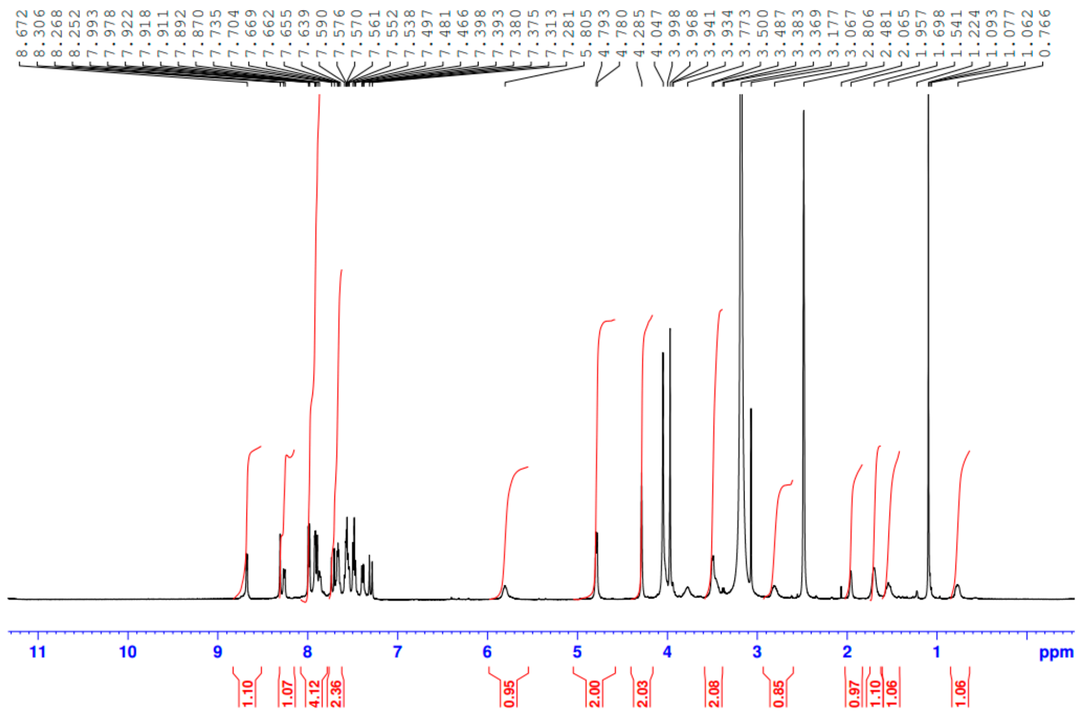

^{13}C-NMR of **7a/A** (125 MHz, DMSO-d6)

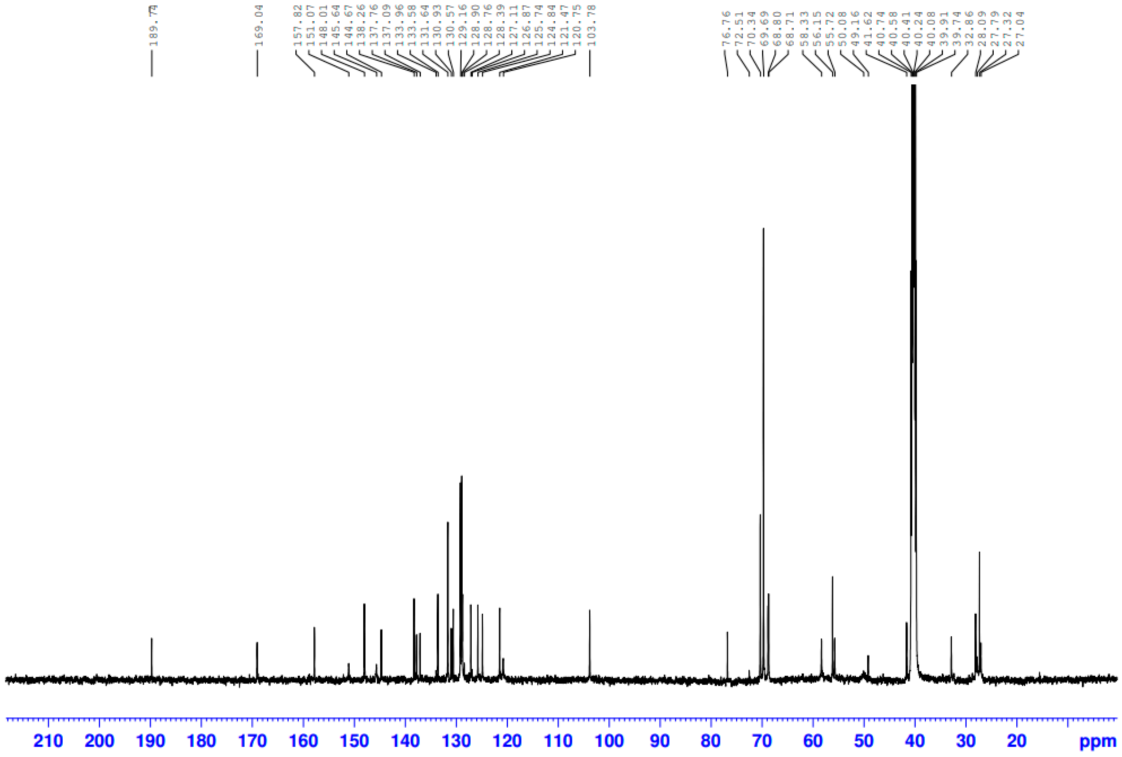

^{1}H-^{13}C-HSQC of **7a**/**A**

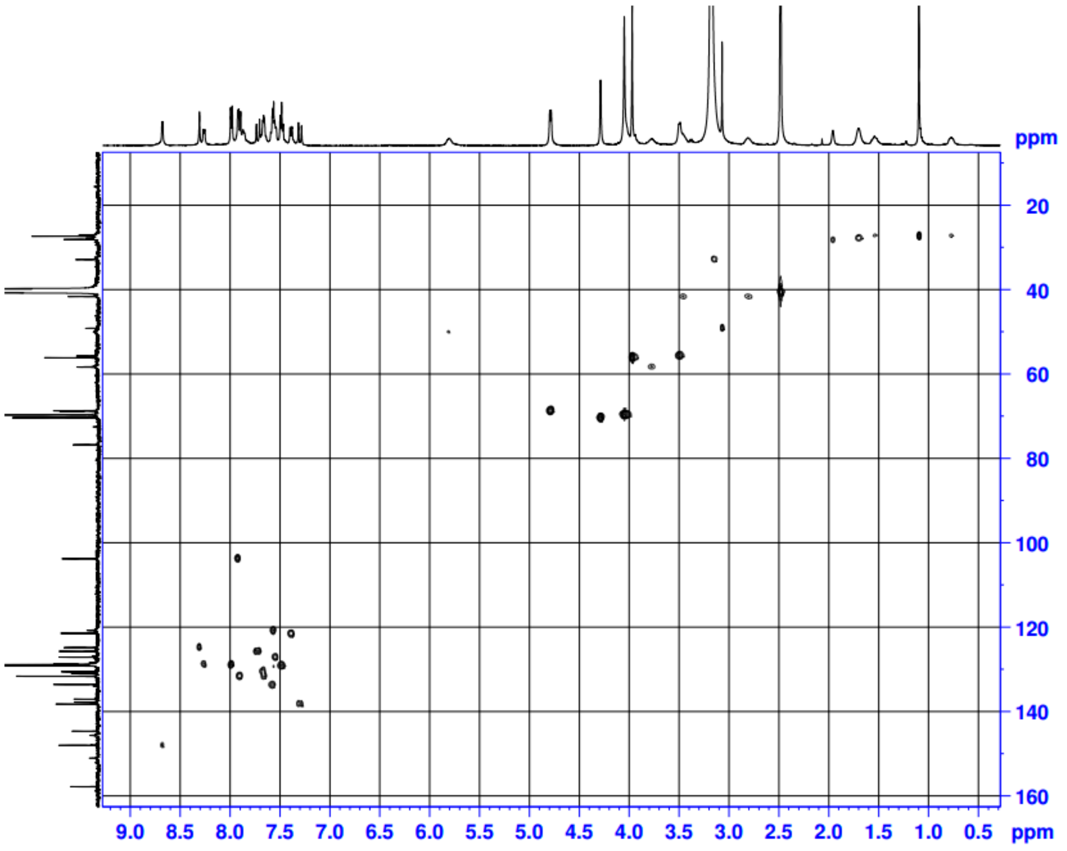

^{1}H-NMR of **7b**/**A** (500 MHz, DMSO-d$_6$)

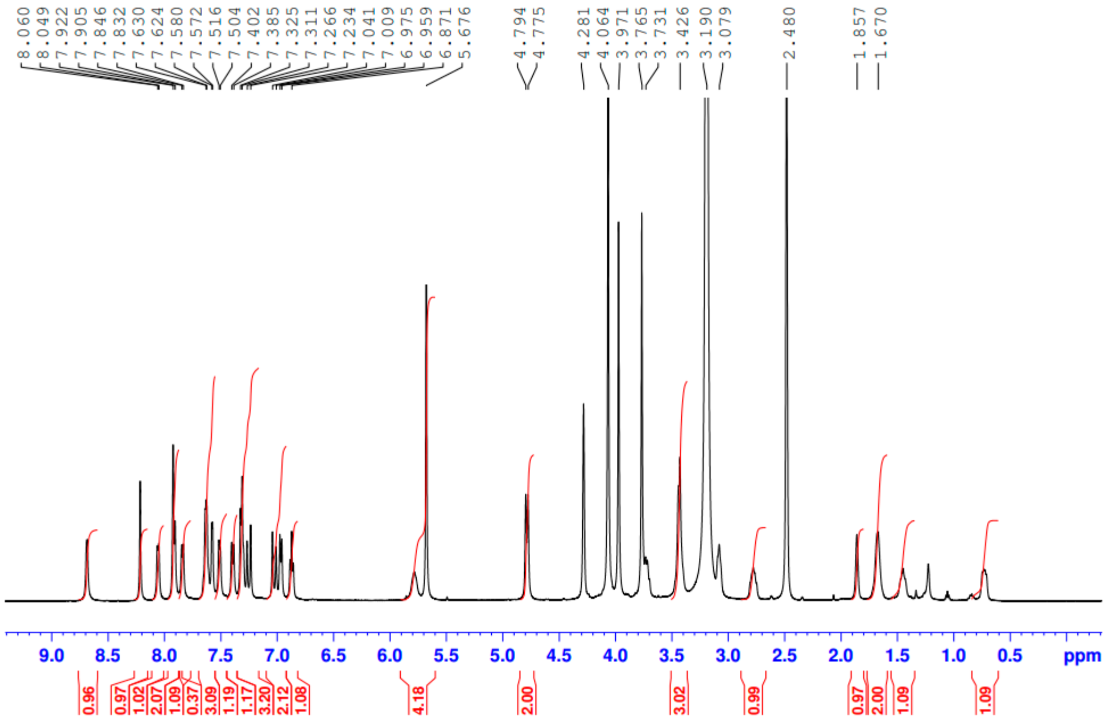

^{13}C-NMR of **7b/A** (125 MHz, DMSO-d$_6$)

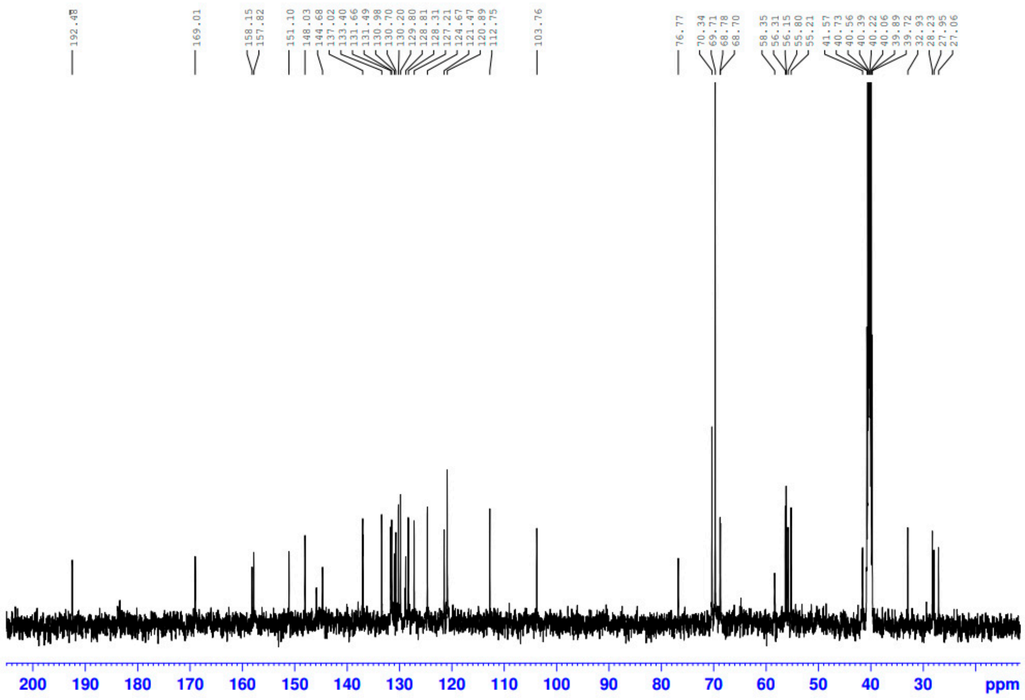

^{1}H-^{13}C-HSQC of **7b/A**

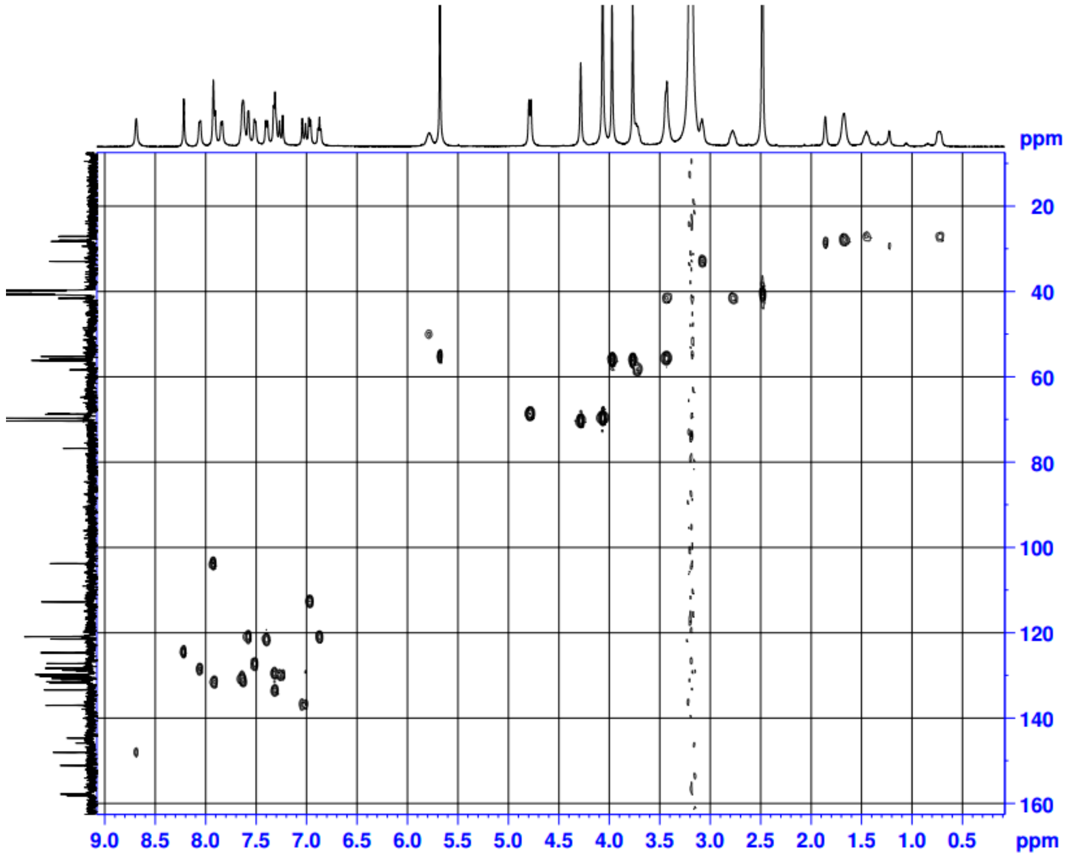

^{1}H-^{13}C-HMBC of **7b**/**A**

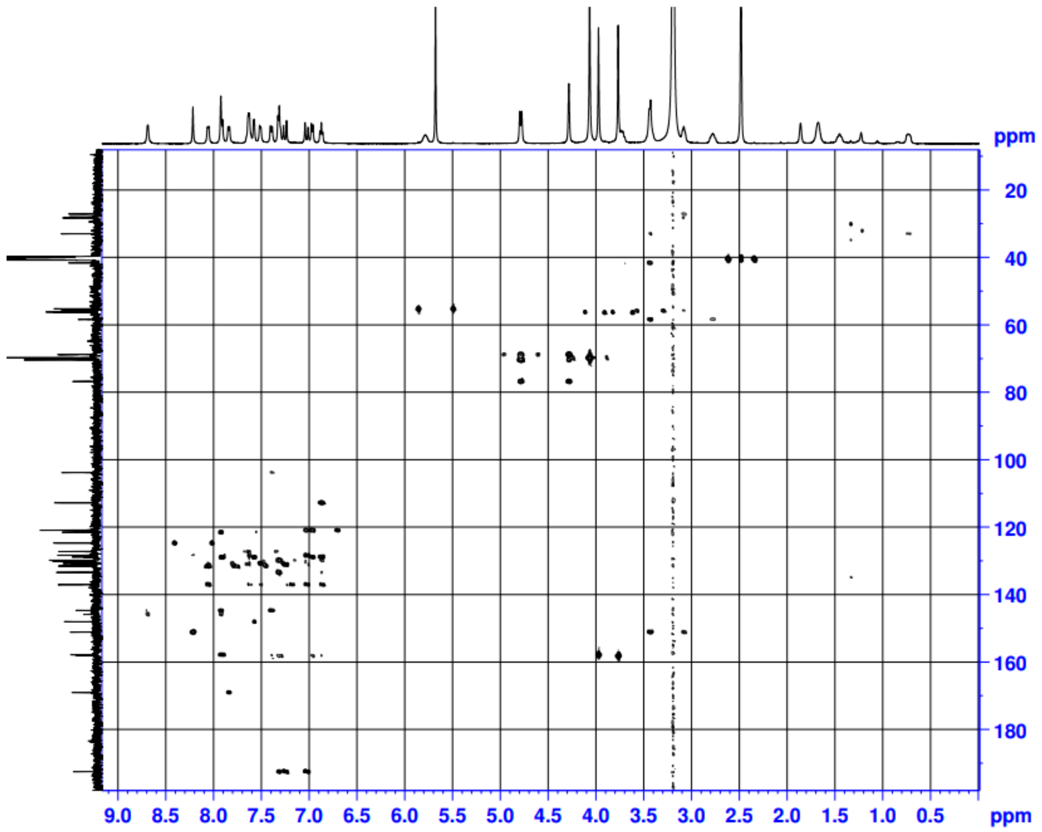

^{1}H-NMR of **7c**/**A** (500 MHz, DMSO-d_6)

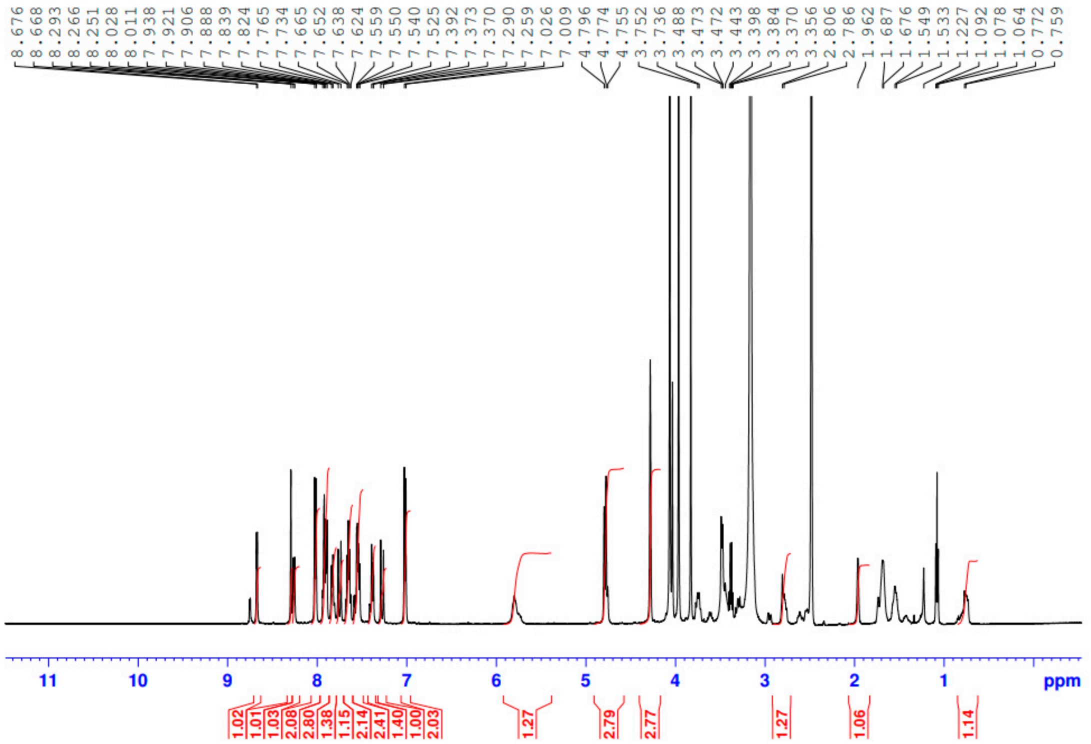

^{13}C-NMR of **7c/A** (125 MHz, DMSO-d6)

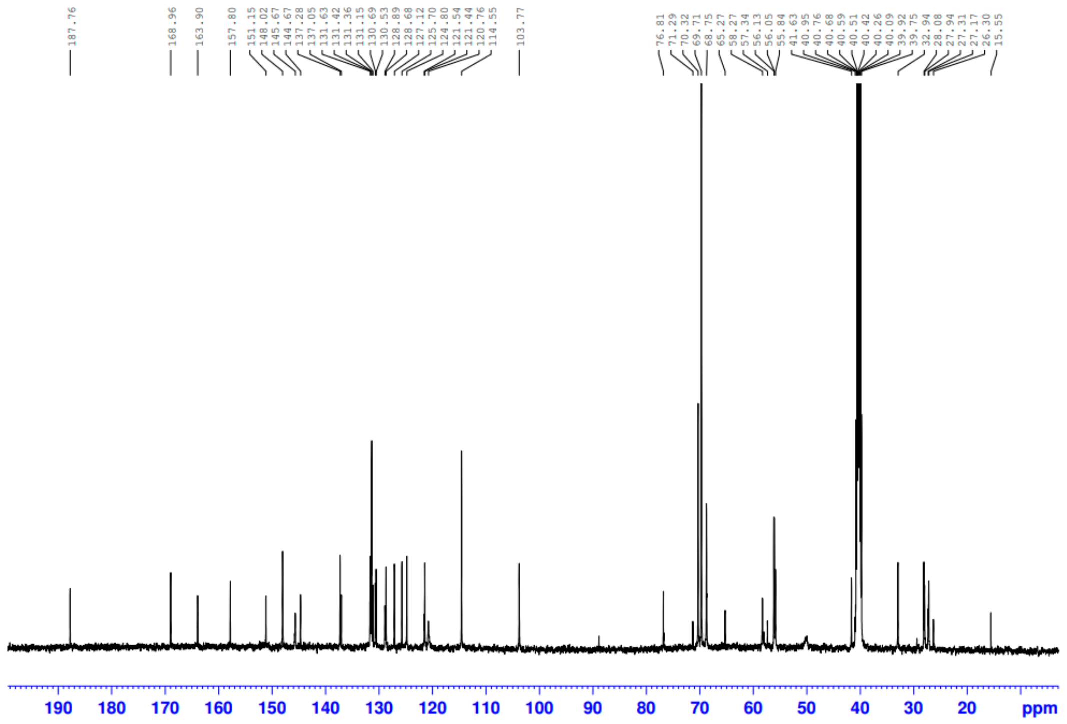

^{1}H-^{13}C-HSQC of **7c/A**

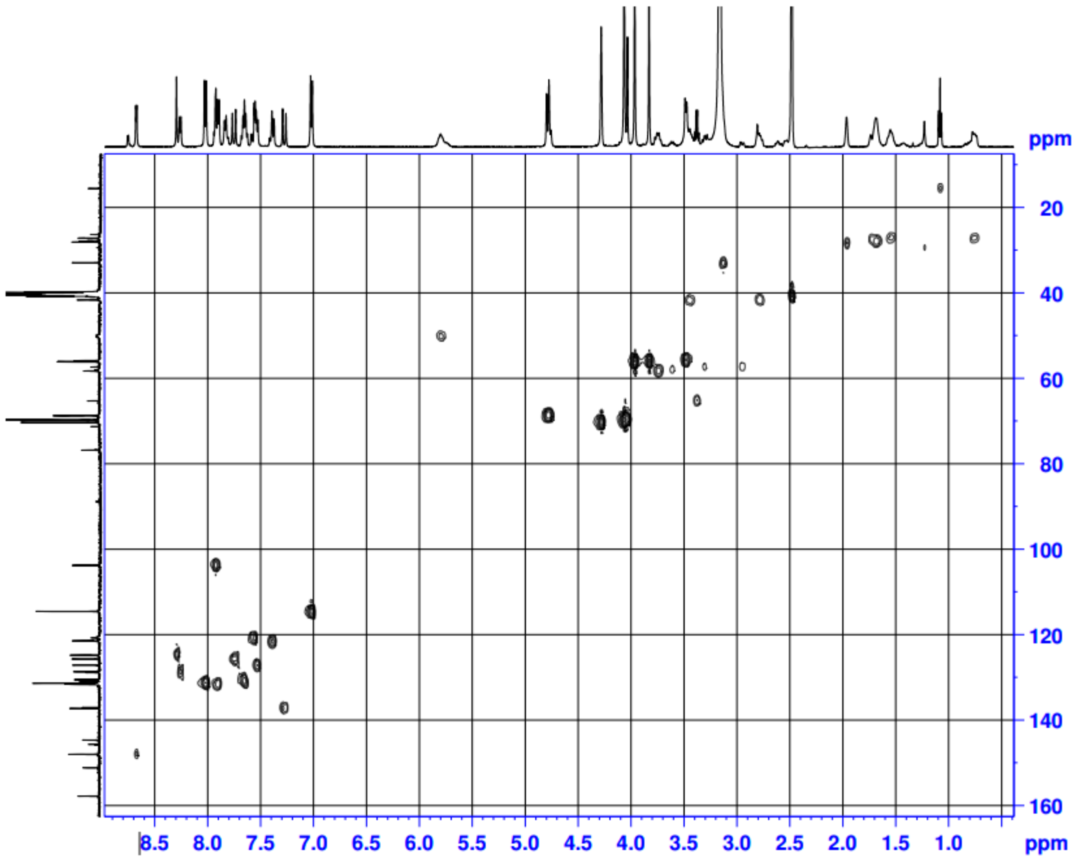

^{1}H-^{13}C-HMBC of **7c**/**A**

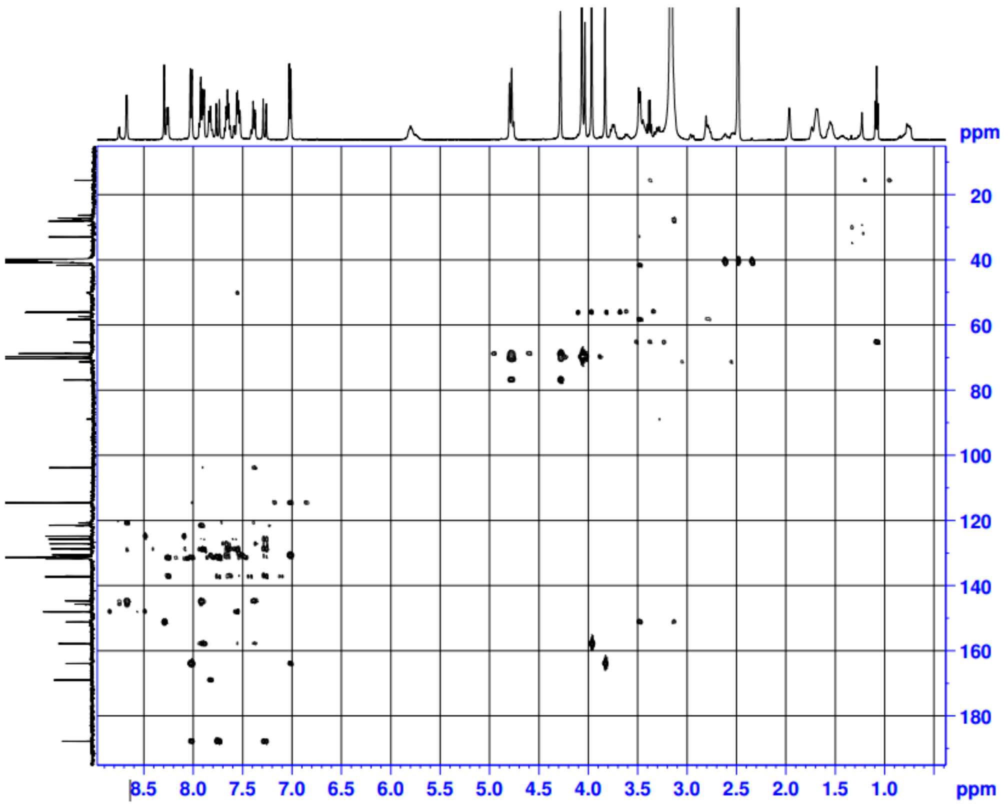

^{1}H-NMR of **7e**/**A** (500 MHz, DMSO-d_6)

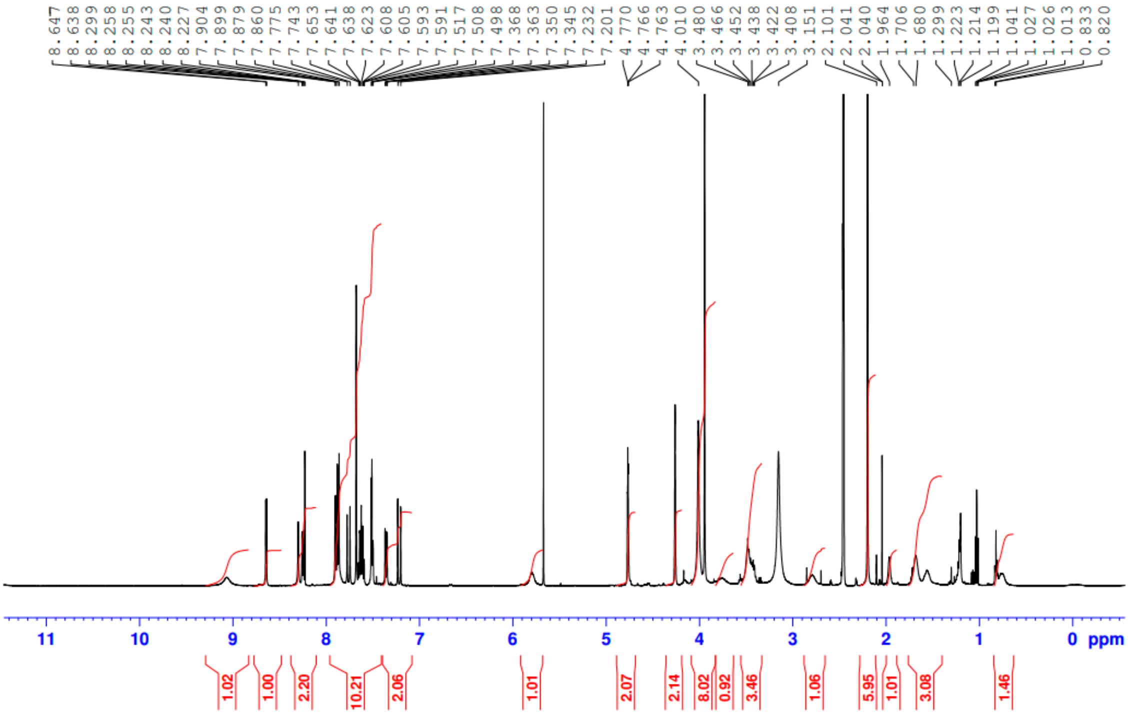

^{13}C-NMR of **7e/A** (125 MHz, DMSO-d$_6$)

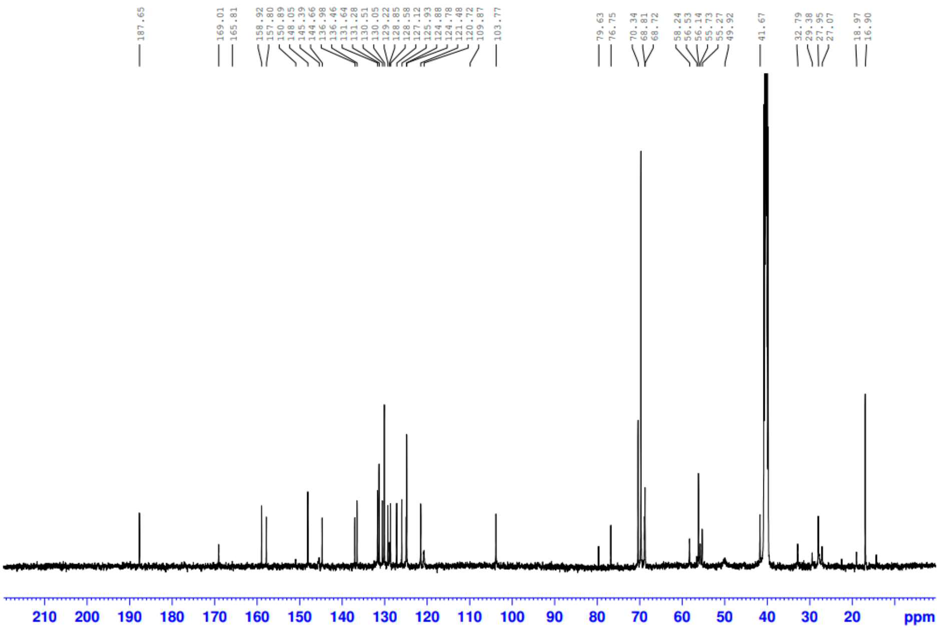

^{1}H-^{13}C-HSQC of **7e/A**

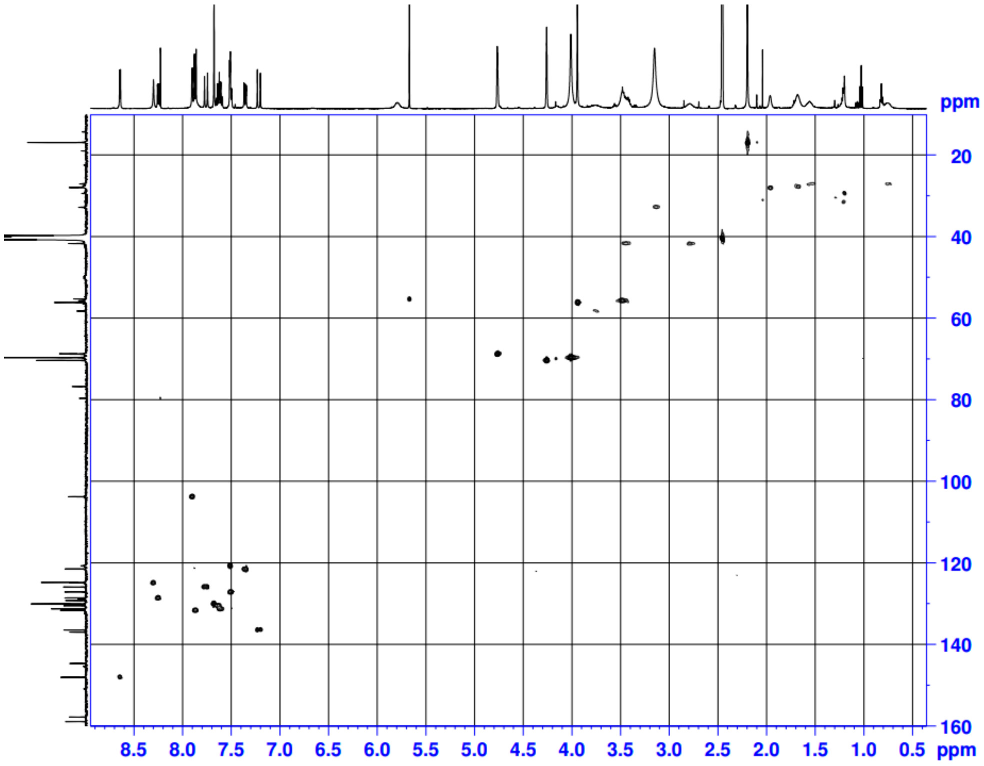

^{1}H-^{13}C-HMBC of **7e/A**

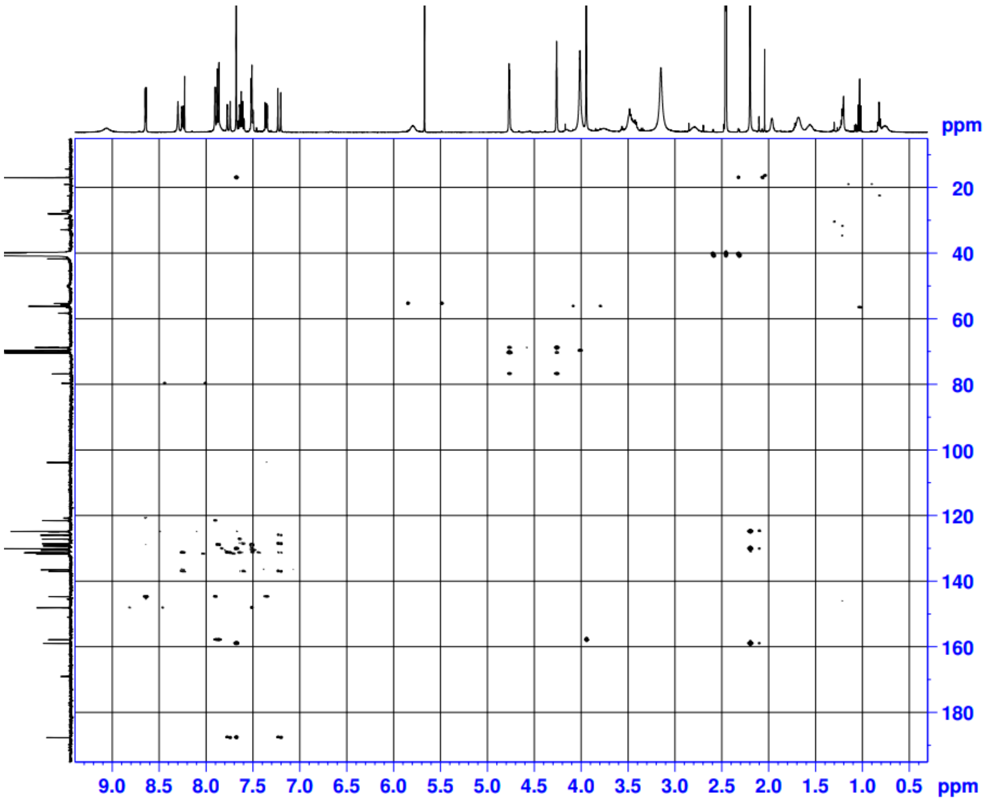

^{1}H-NMR of **9b/A** (500 MHz, DMSO-d$_6$)

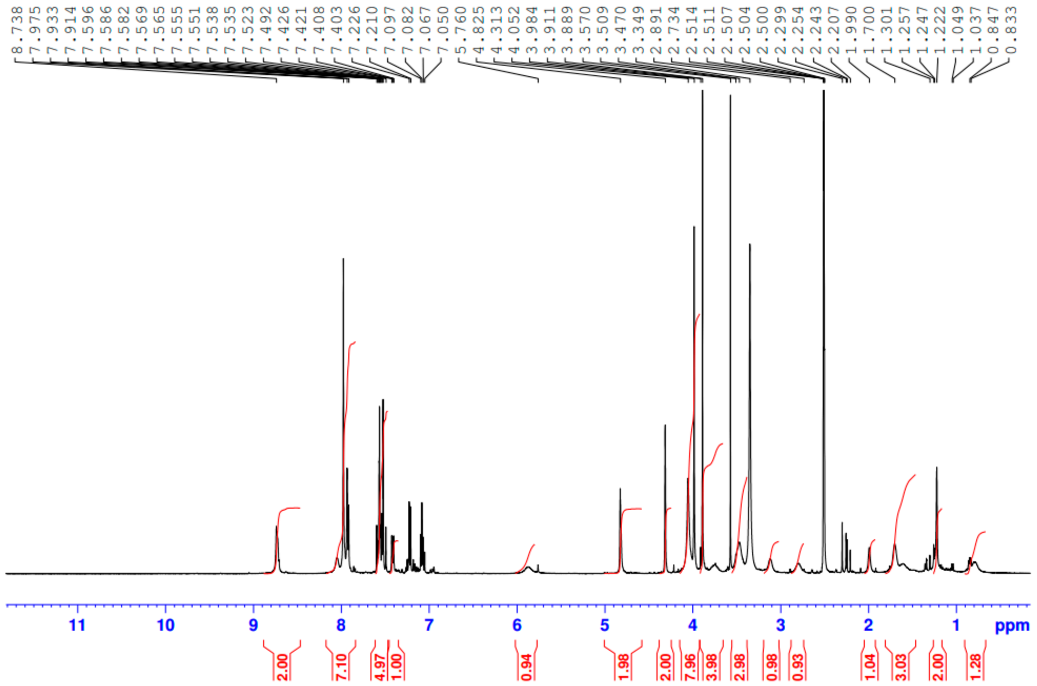

^{13}C-NMR of **9b/A** (125 MHz, DMSO-d$_6$)

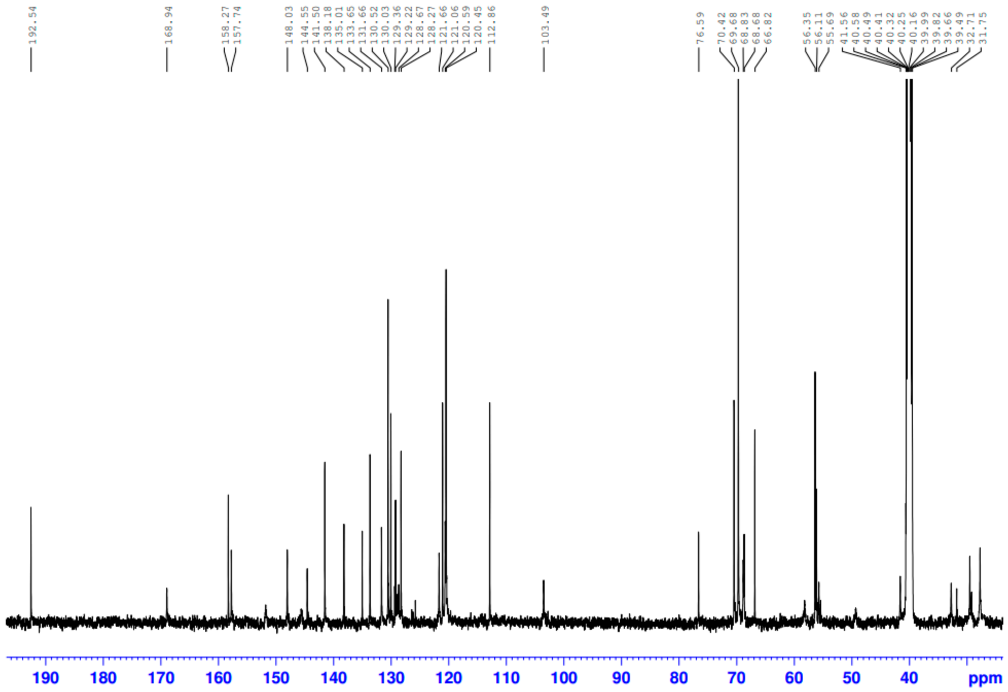

^{1}H-^{13}C-HMBC of **9b/A**

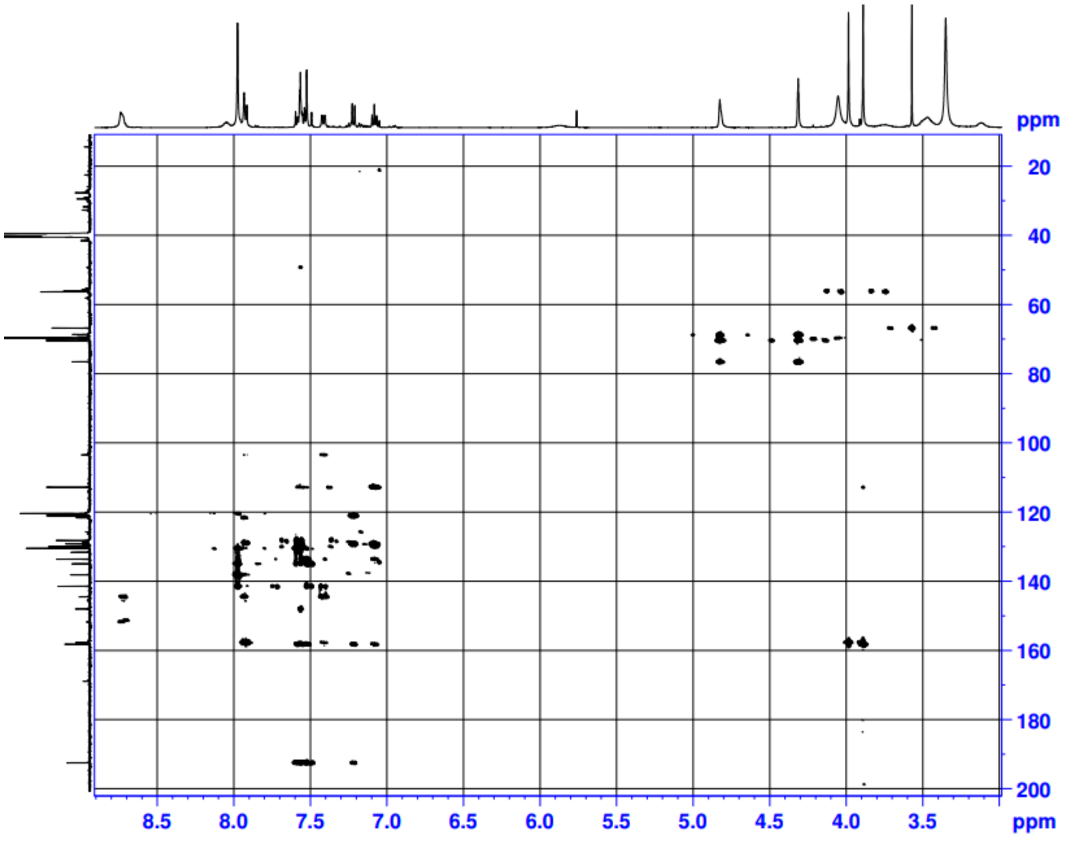

^{1}H-NMR of **9c/A** (500 MHz, DMSO-d6)

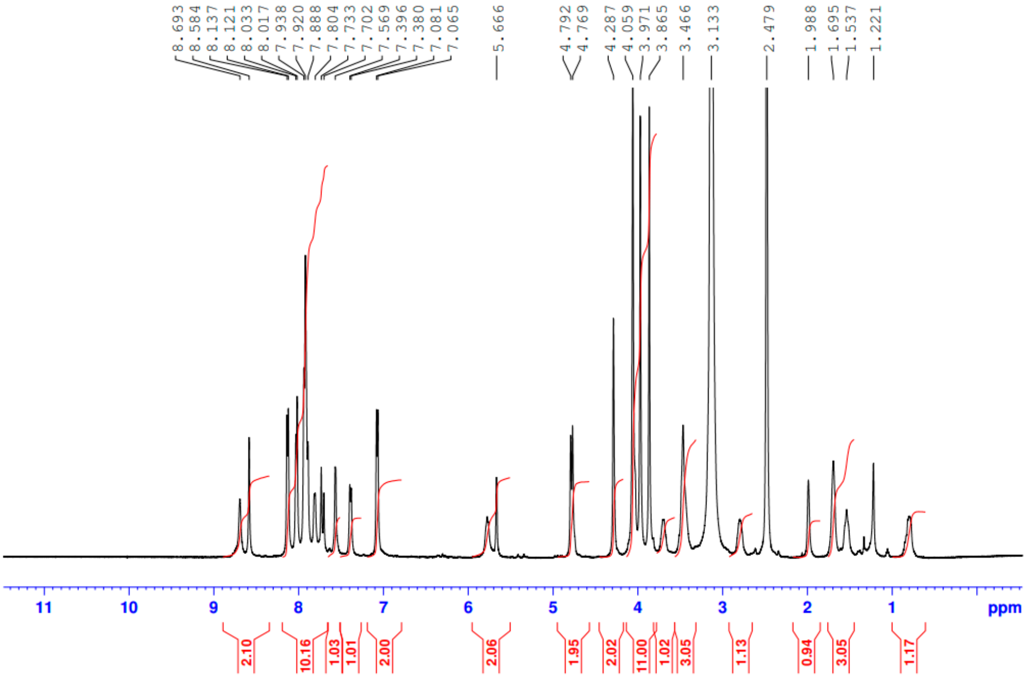

^{13}C-NMR of **9c/A** (125 MHz, DMSO-d6)

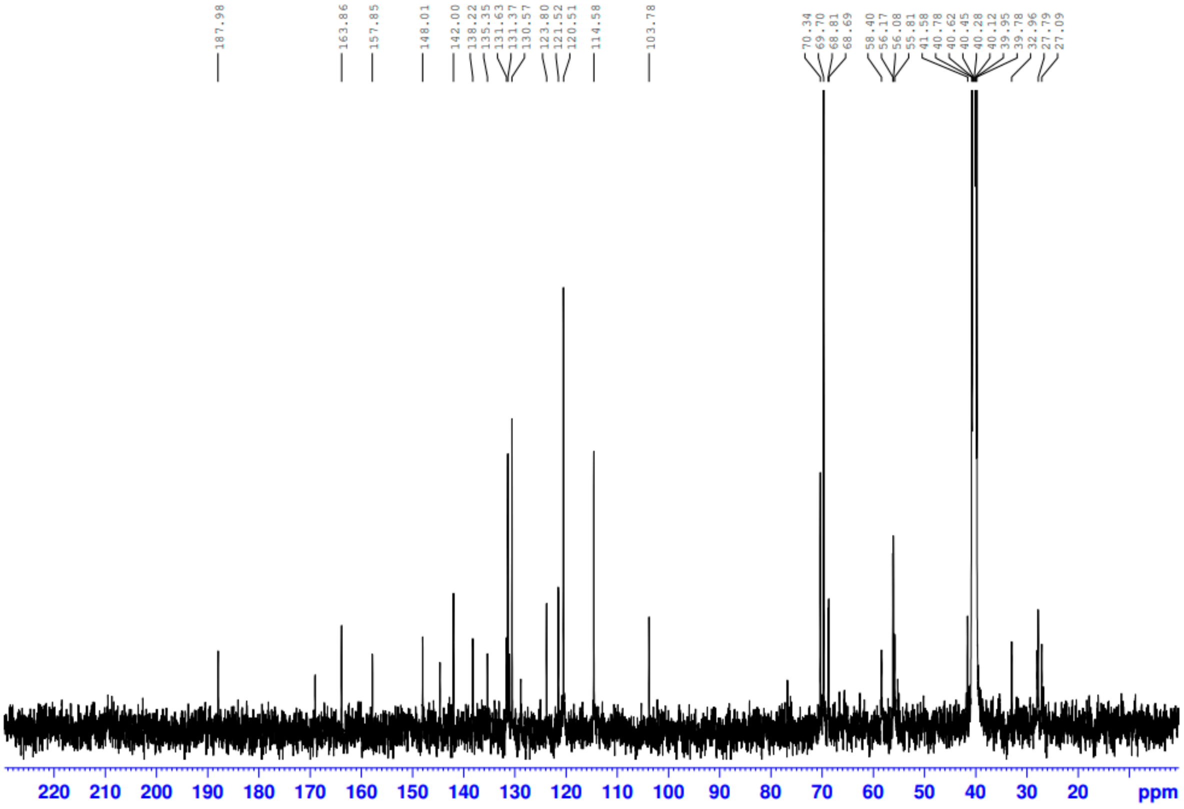

^{1}H-^{13}C-HSQC of **9c/A**

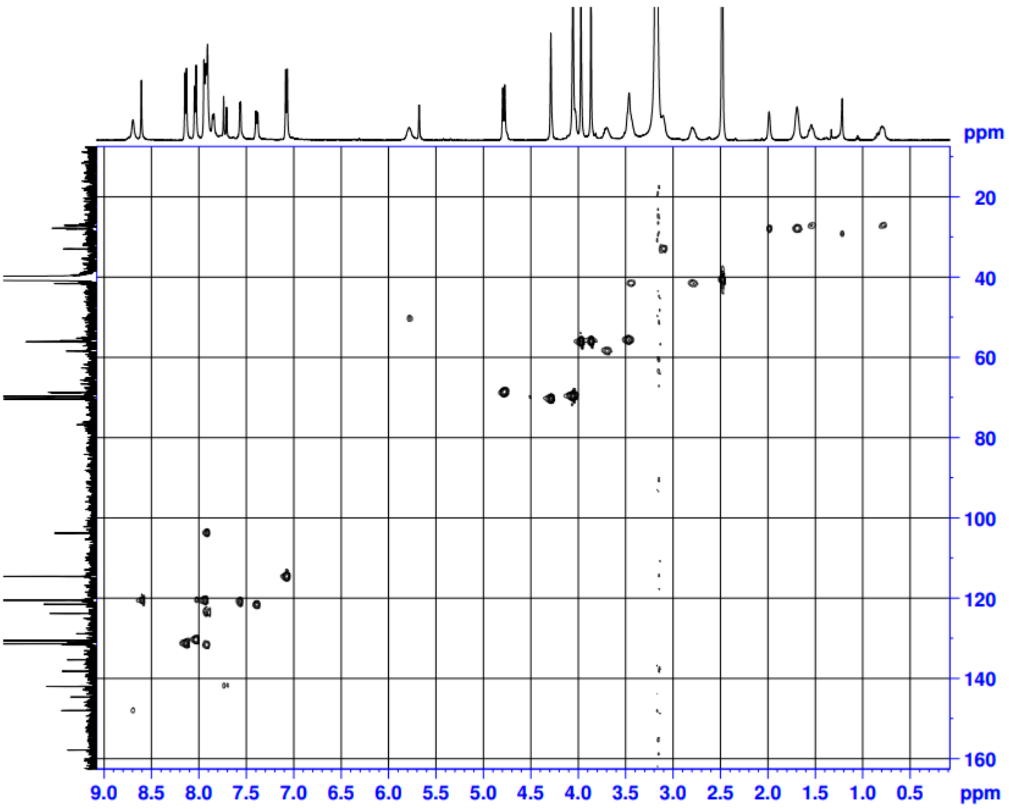

^{1}H-^{13}C-HMBC of **9c/A**

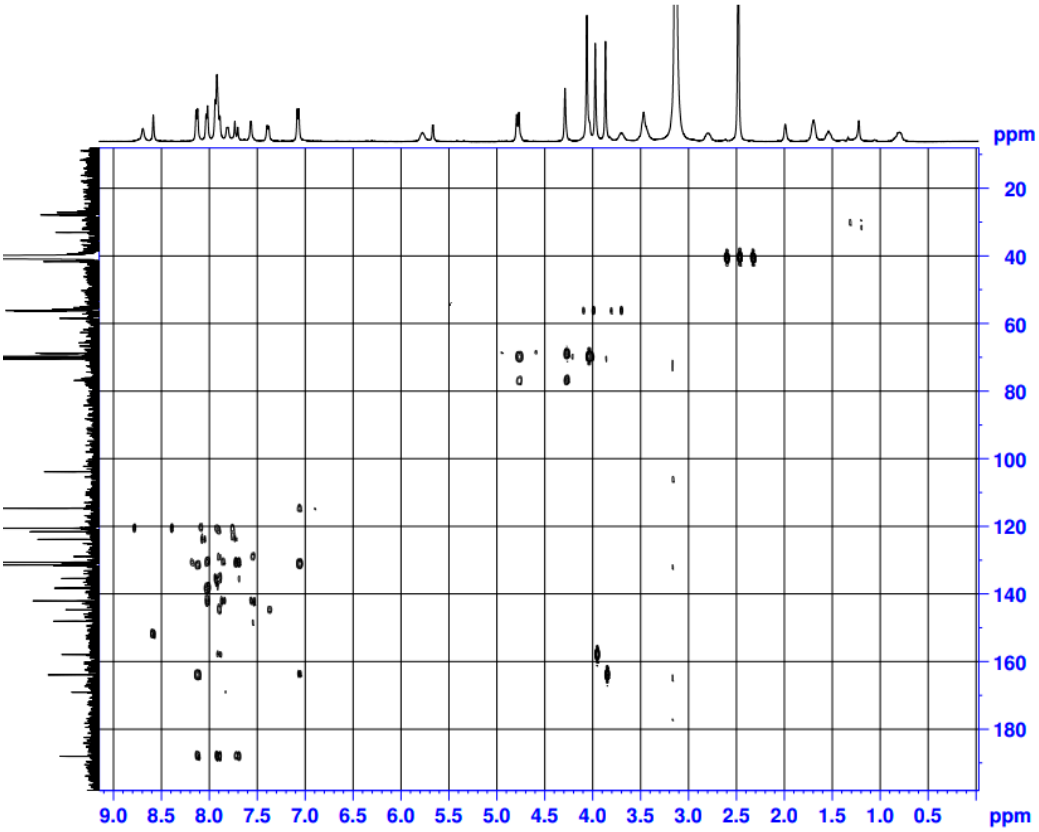

^{1}H-NMR of **9d/A** (500 MHz, DMSO-d$_6$)

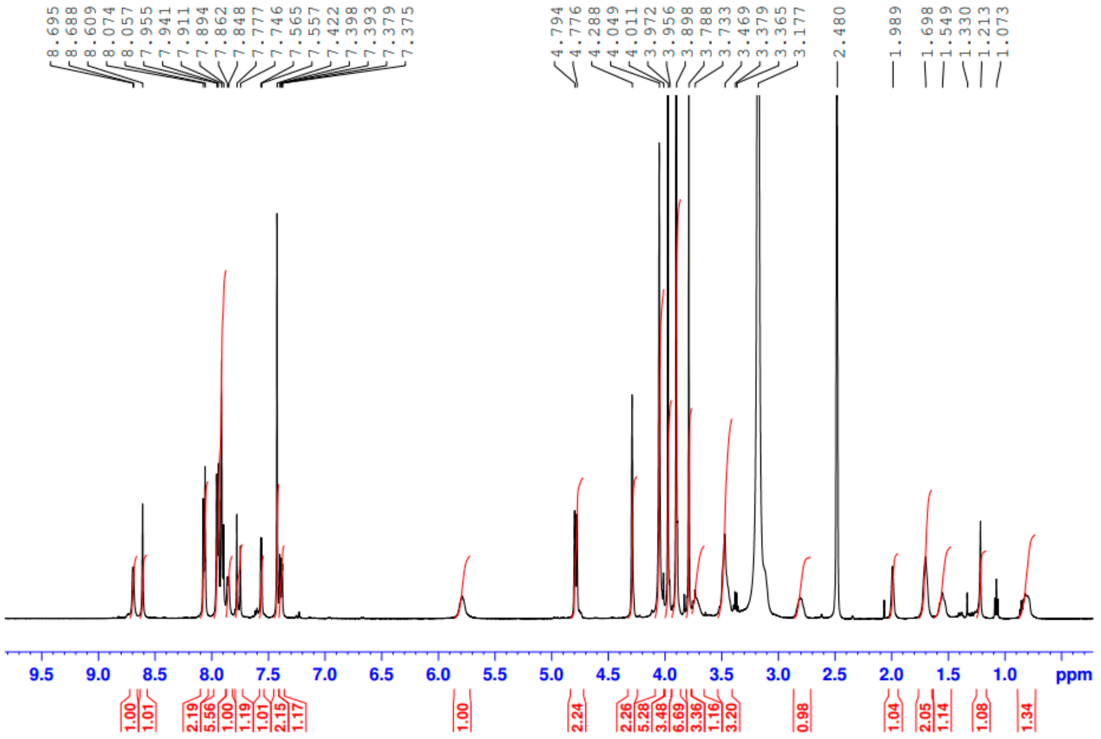

^{13}C-NMR of **9d/A** (125 MHz, DMSO-d$_6$)

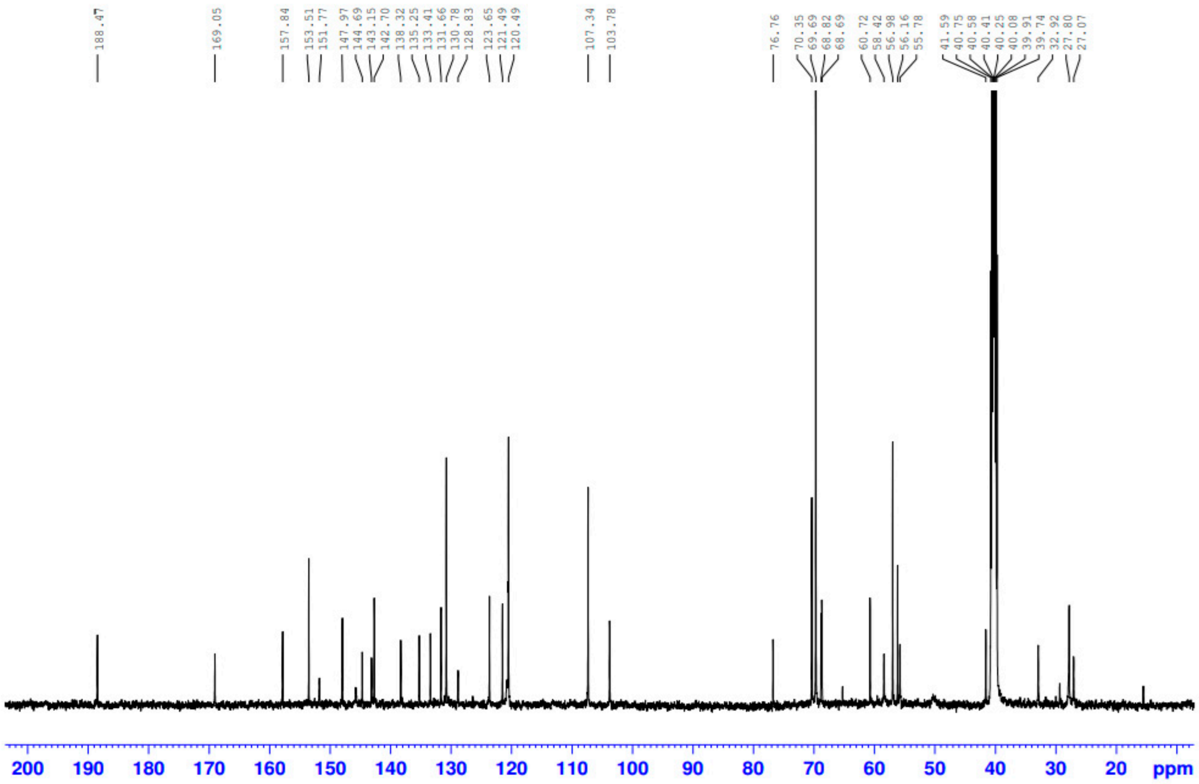

^{1}H-^{13}C-HSQC of **9d/A**

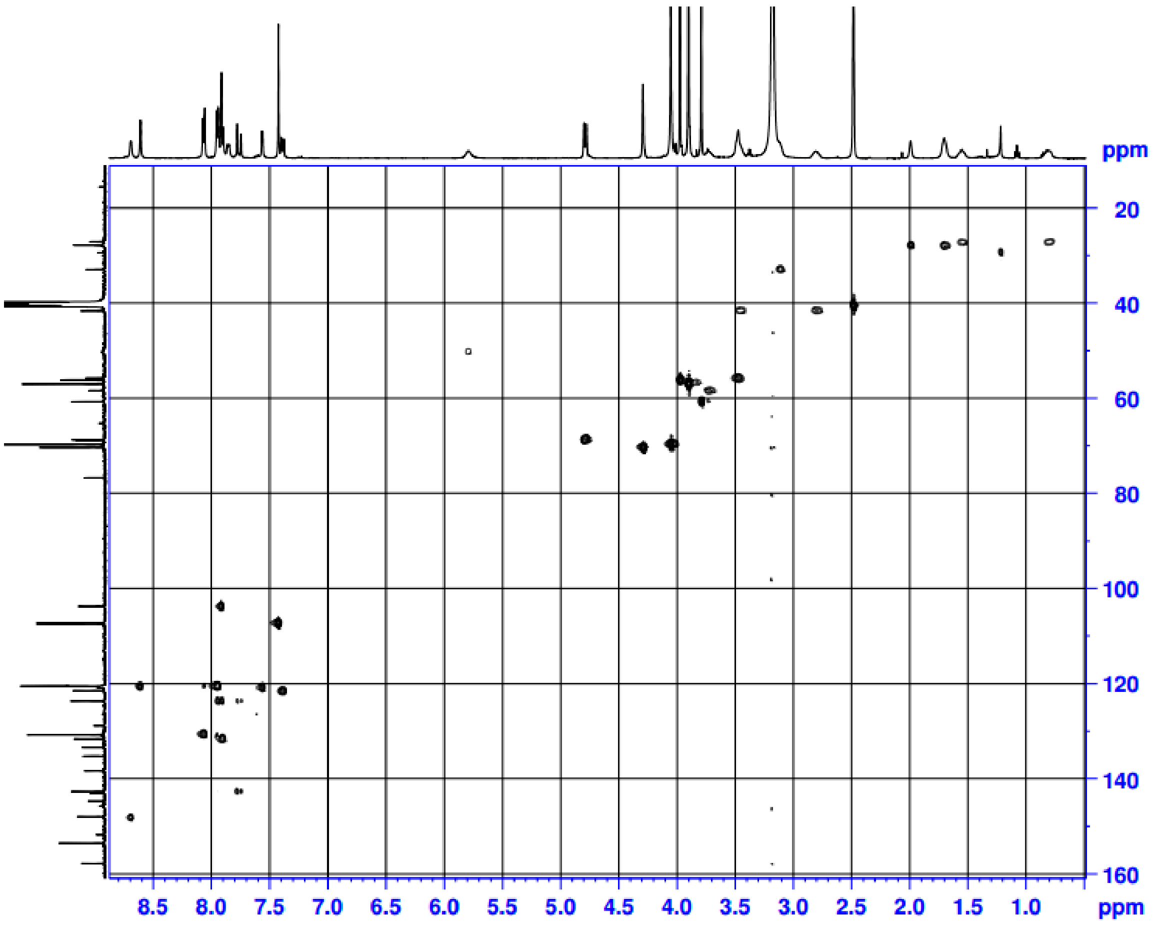

^{1}H-^{13}C-HMBC of **9d/A**

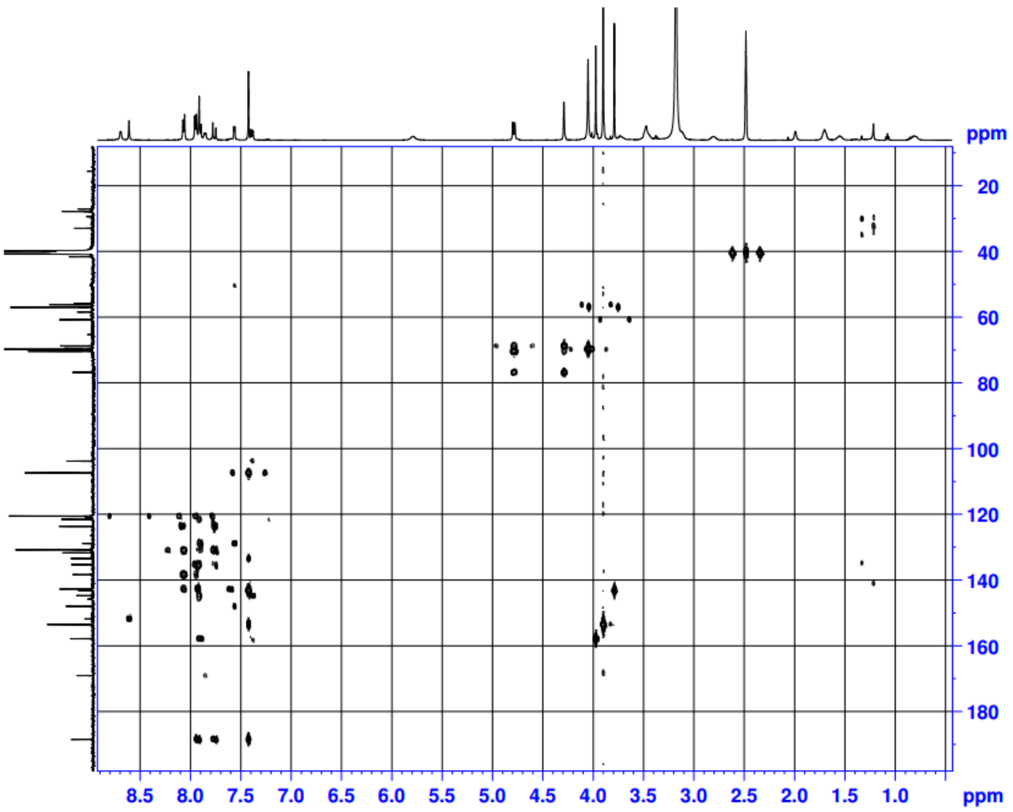

NOESY of **9d/A** (500 MHz, DMSO-d_6)

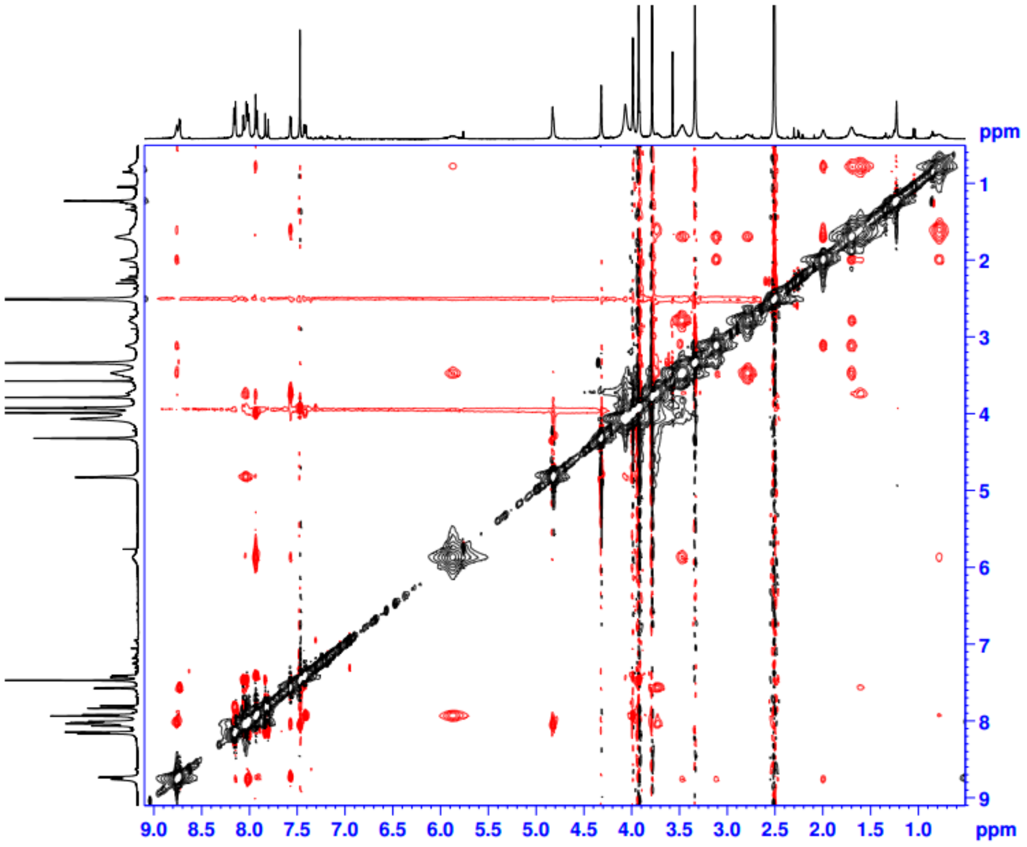

^{1}H-NMR of **7e**/**B** (500 MHz, DMSO-d$_6$)

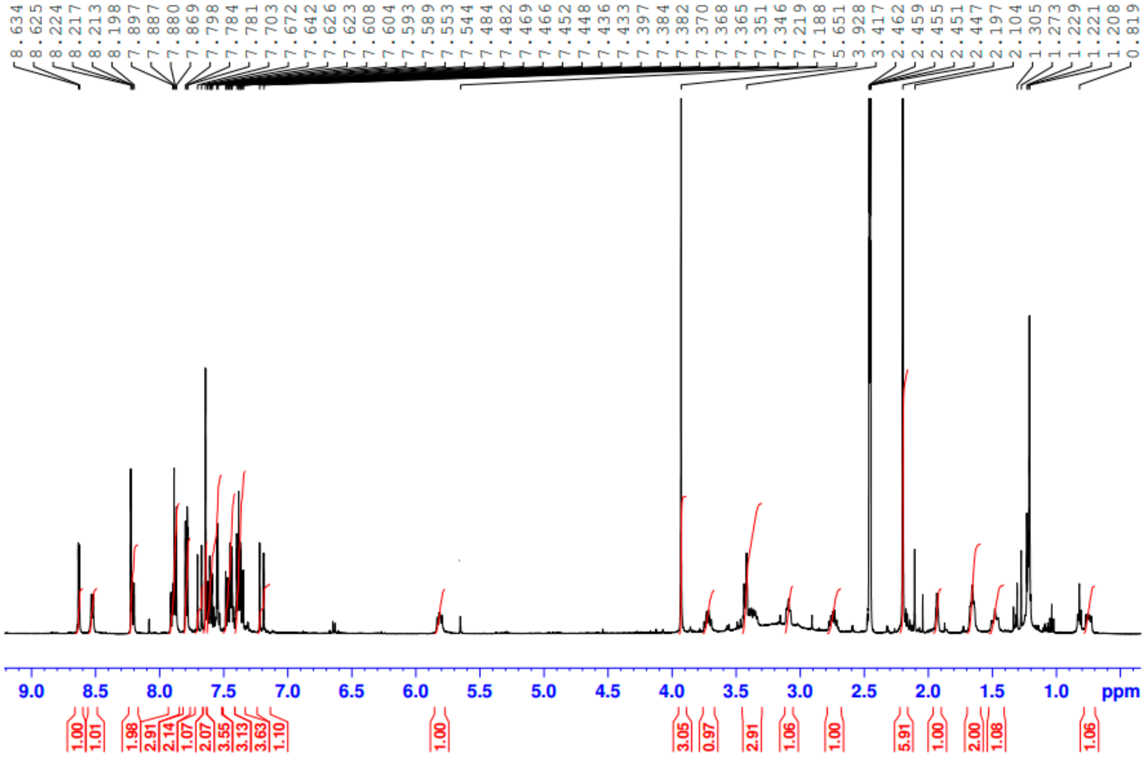

^{13}C-NMR of **7e**/**B** (125 MHz, DMSO-d$_6$)

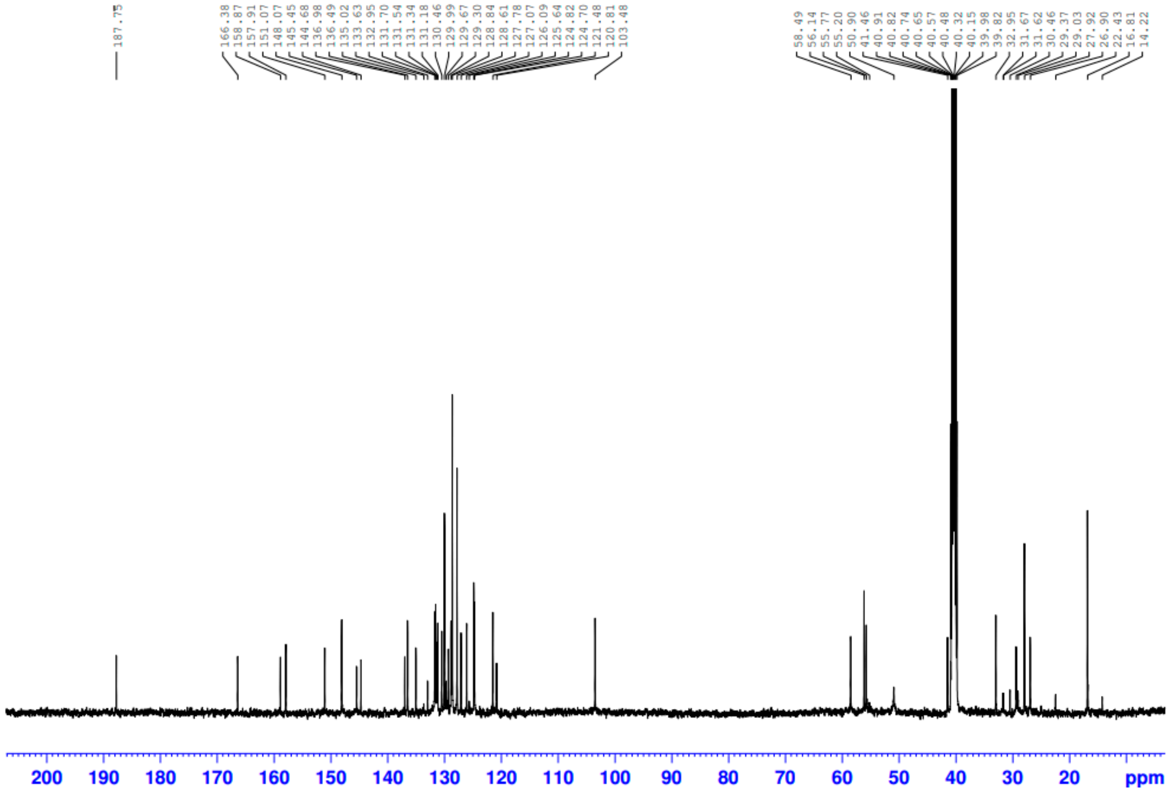

^{1}H-^{13}C-HSQC of **7e/B**

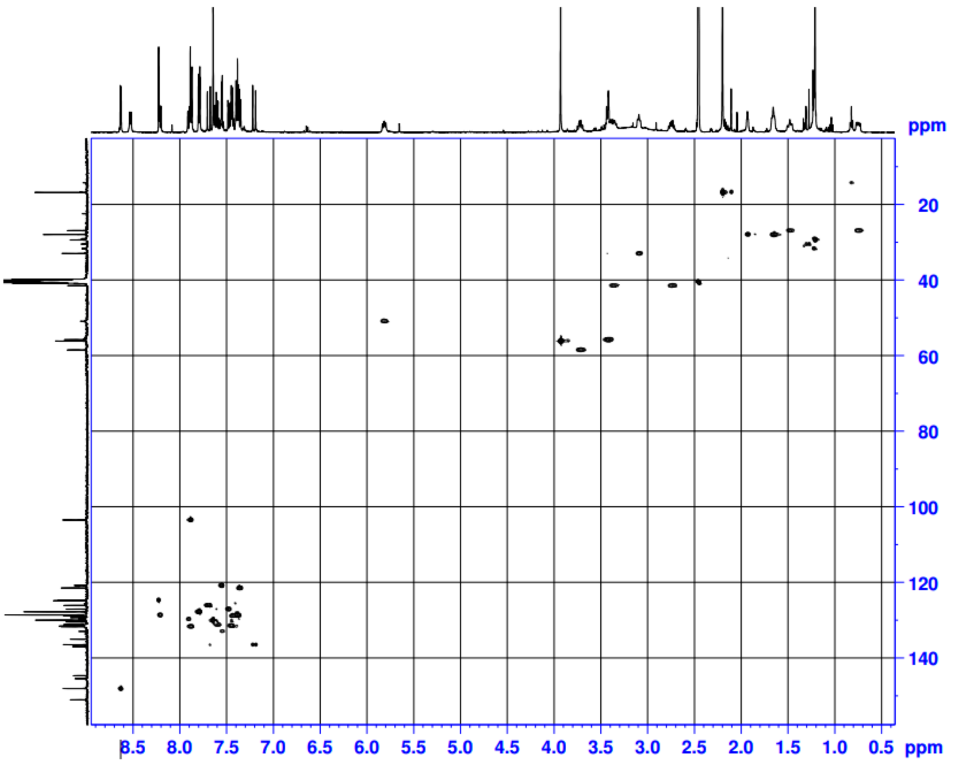

^{1}H-^{13}C-HMBC of **7e/B**

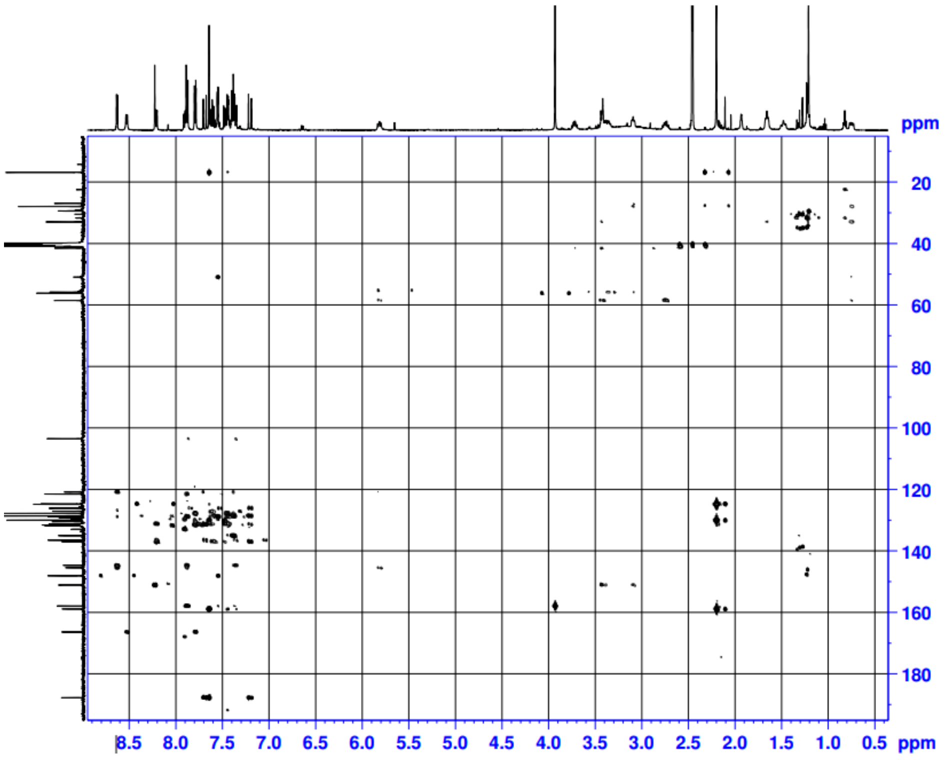

^{1}H-NMR of **9d/B** (500 MHz, DMSO-d$_6$)

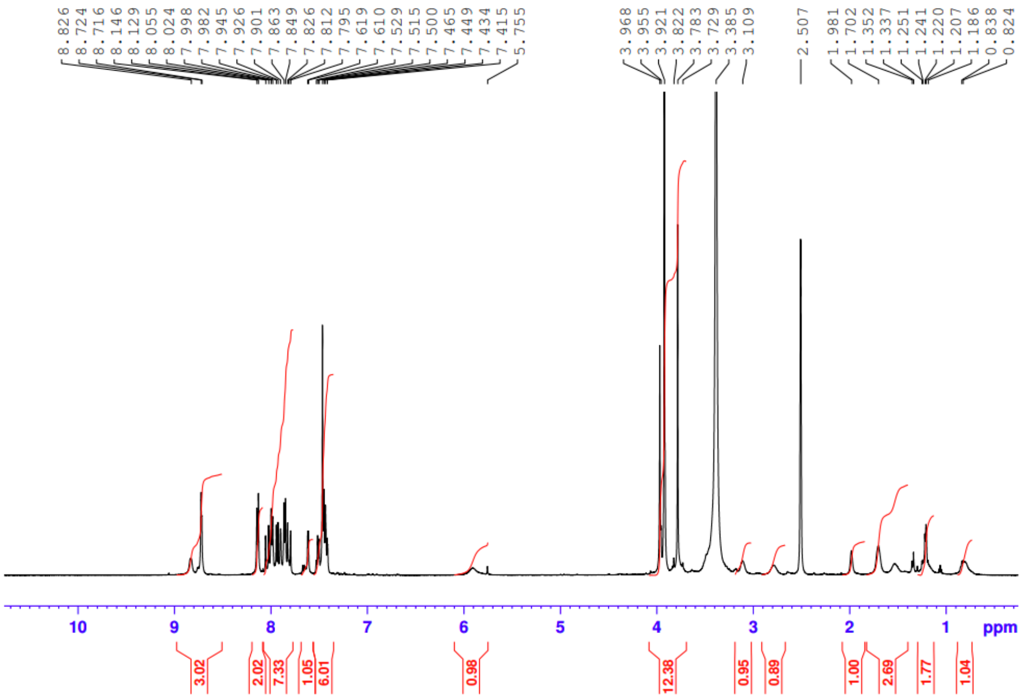

^{13}C-NMR of **9d/B** (125 MHz, DMSO-d$_6$)

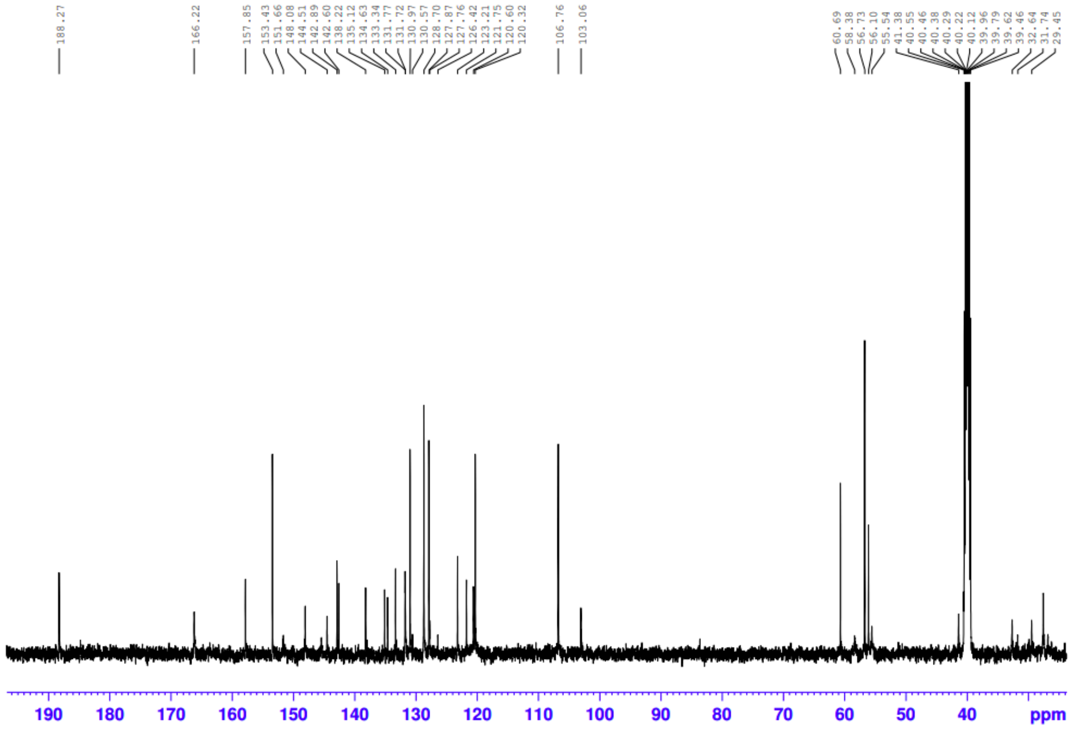

^{1}H-^{13}C-HSQC of **9d/B**

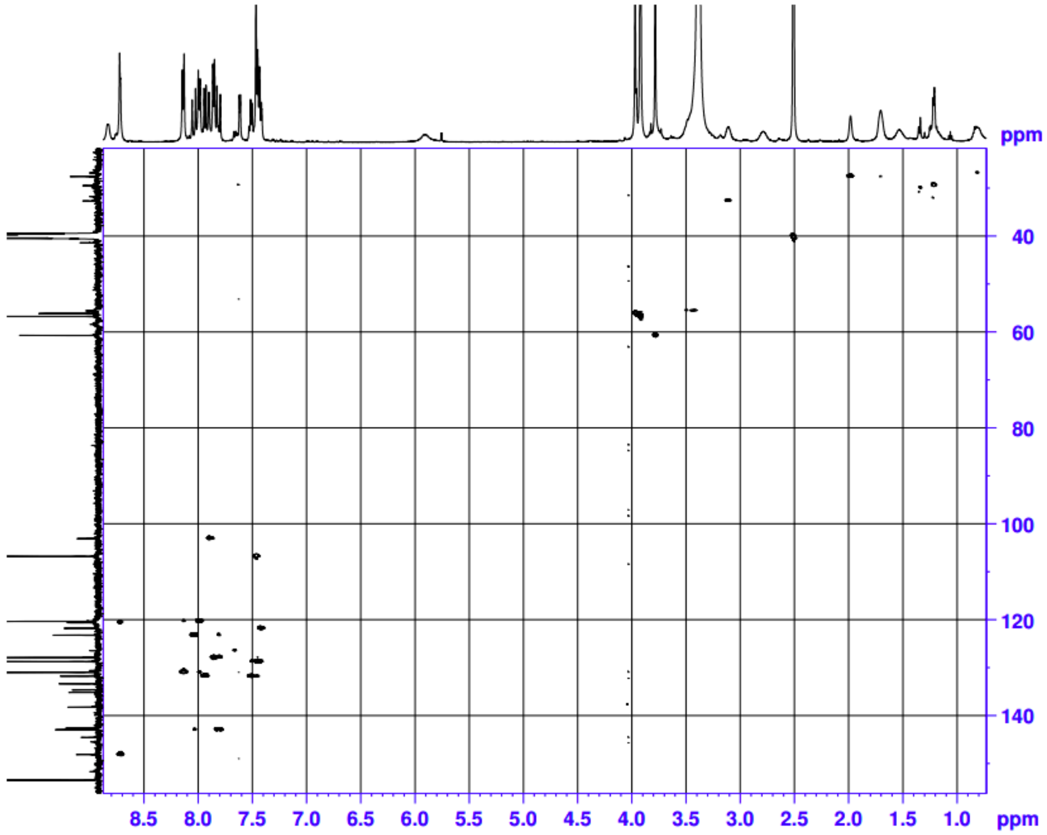

^{1}H-^{13}C-HMBC of **9d/B**

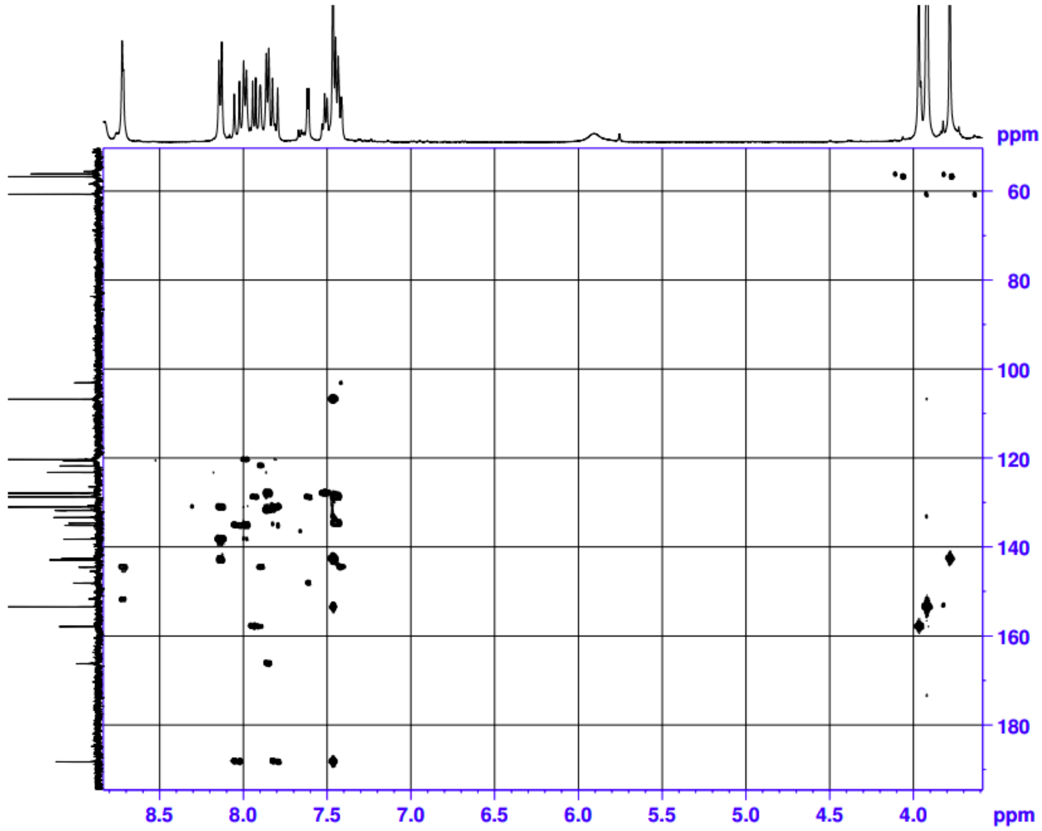

^{1}H-NMR of **8**/**A** (500 MHz, DMSO-d6)

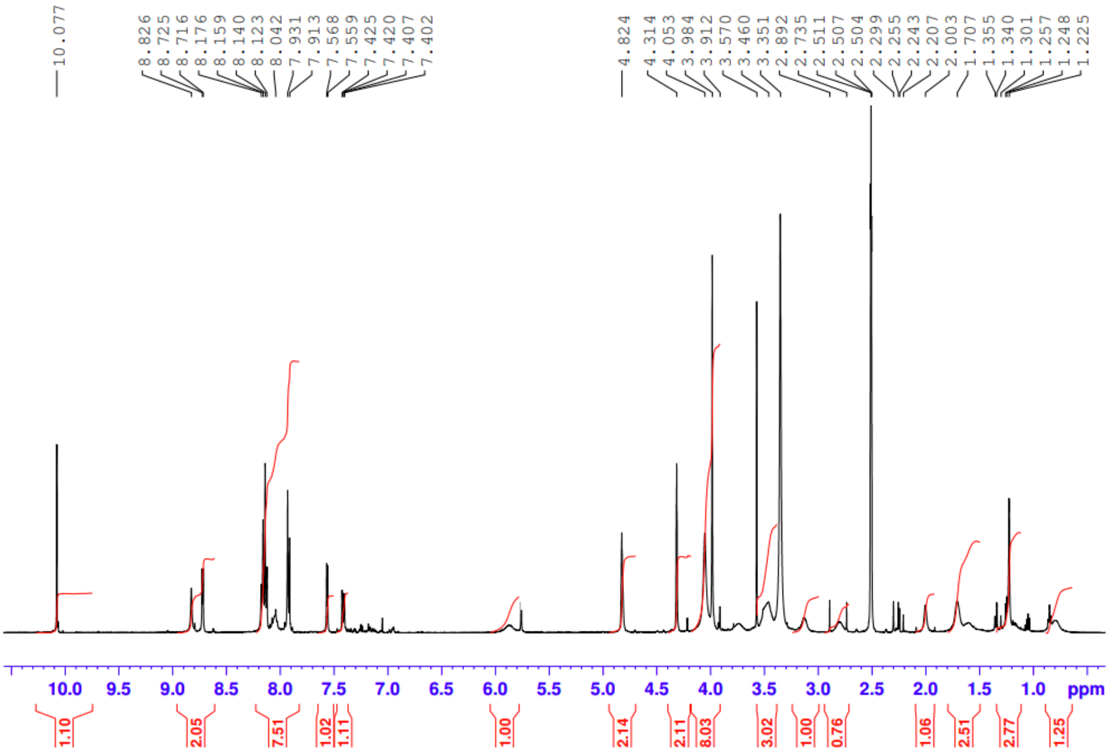

^{13}C-NMR of **8**/A (125 MHz, DMSO-d6)

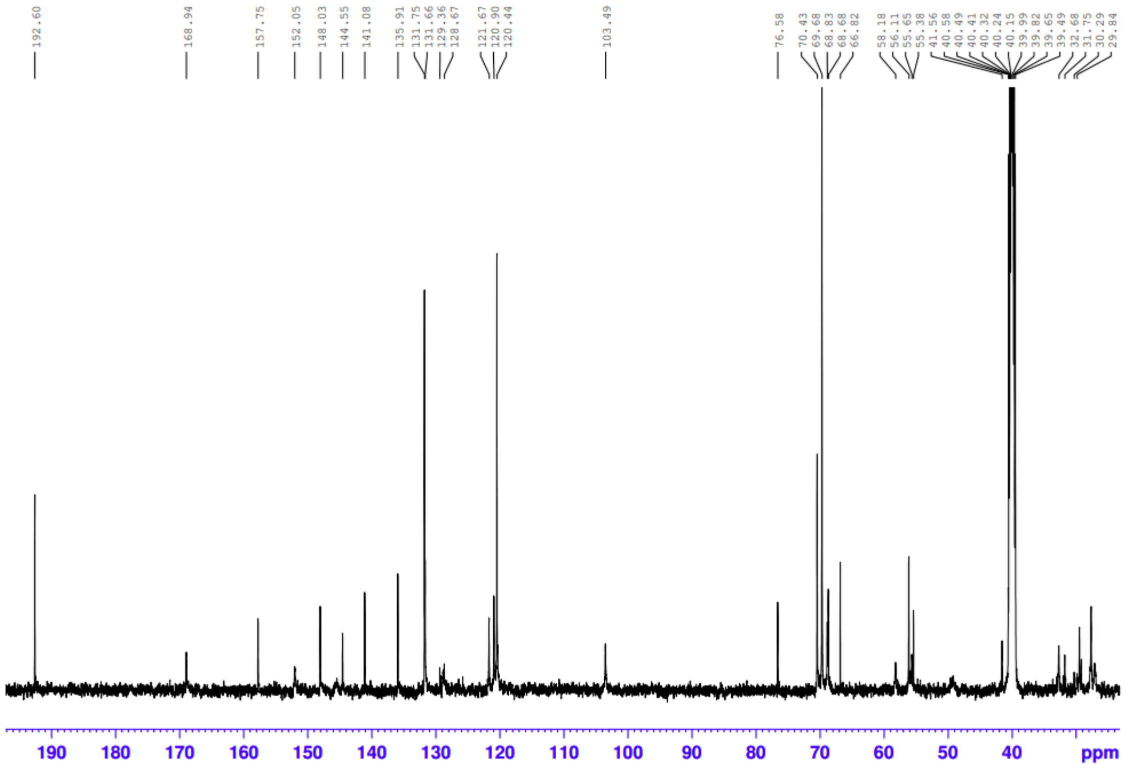

^{1}H-NMR of **8**/**B** (500 MHz, DMSO-d6)

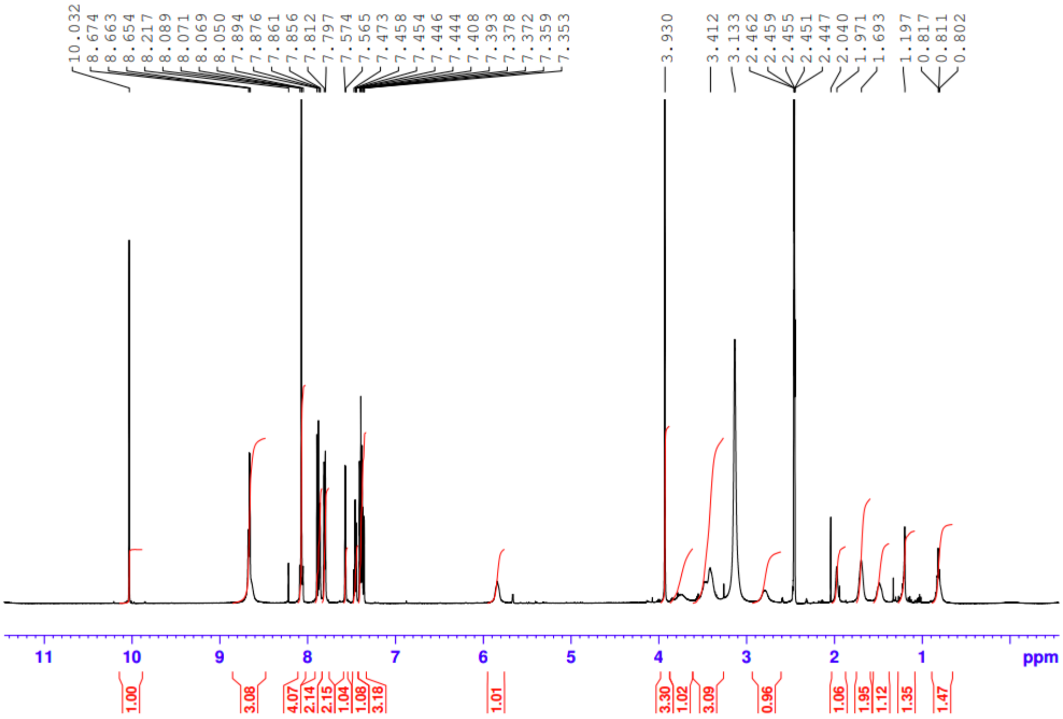

^{13}C-NMR of **8**/B (125 MHz, DMSO-d6)

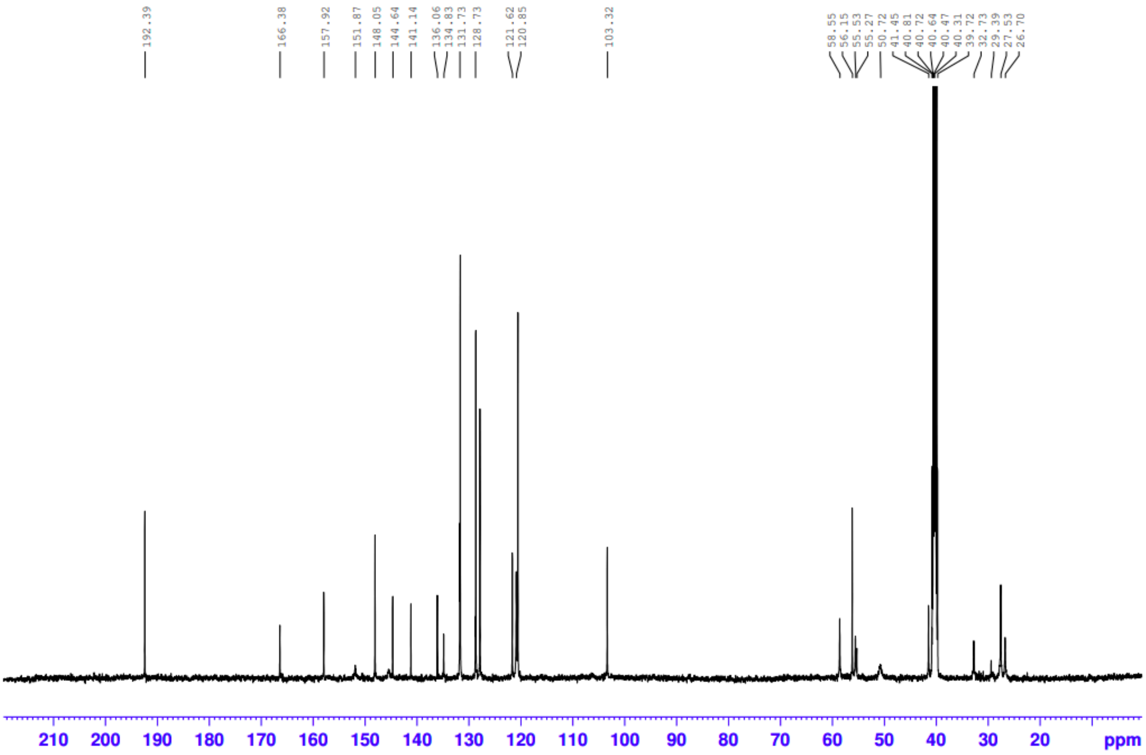

^{1}H-^{13}C-HSQC of **8**/**B**

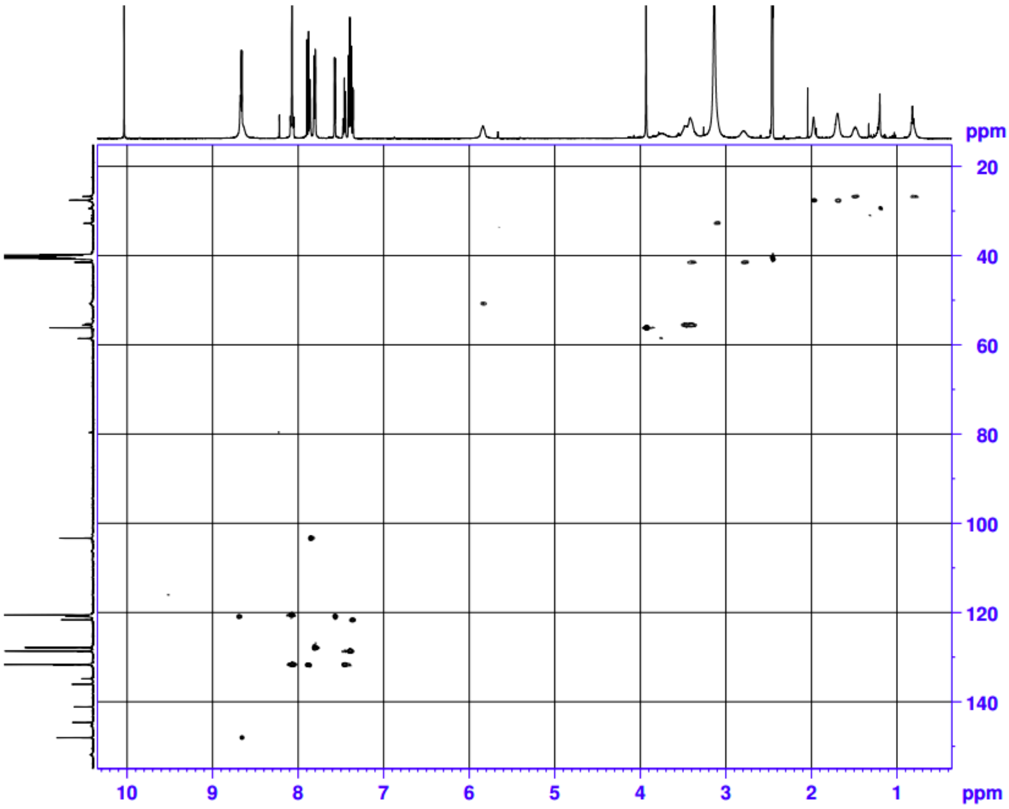

^{1}H-^{13}C-HMBC of **8**/**B**

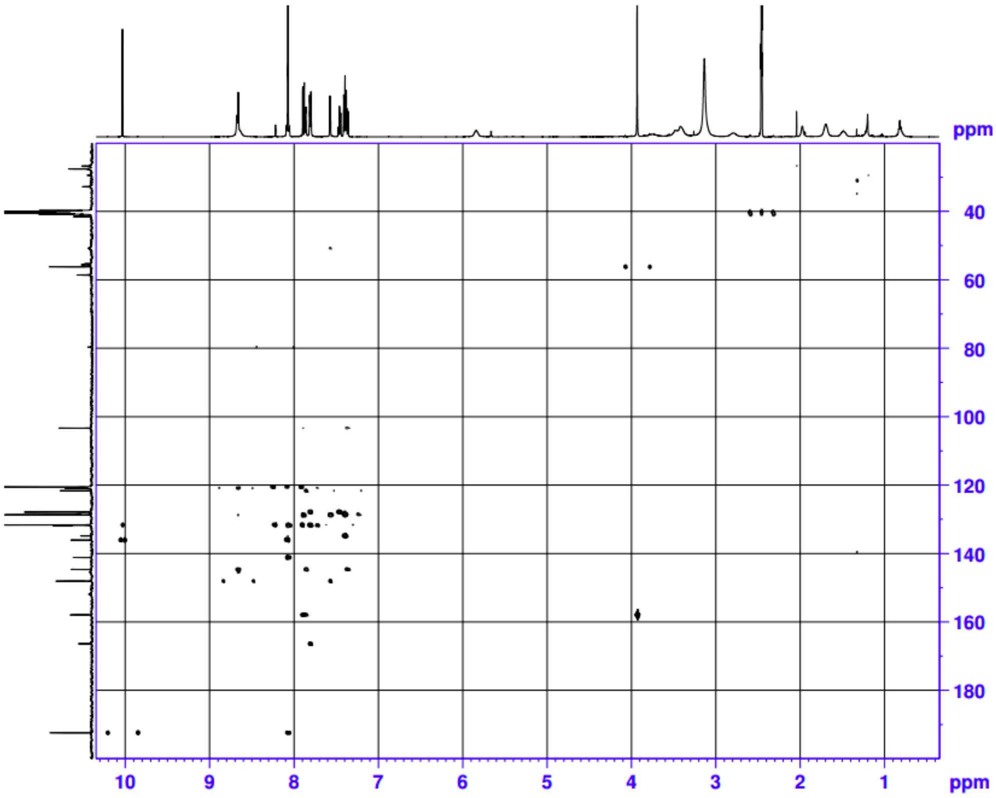

Supplement: Supplementary file 1 [file molecules-29-00375-s001.zip › molecules-2789828-supplementary.pdf]
